# Conformational analysis of difluoromethylornithine: factors influencing its gas-phase and bioactive conformations

**DOI:** 10.3762/bjoc.22.17

**Published:** 2026-02-05

**Authors:** Matheus P Freitas

**Affiliations:** 1 Department of Chemistry, Institute of Natural Sciences, Federal University of Lavras, 37200-900, Lavras, MG, Brazil

**Keywords:** conformational analysis, difluoromethylornithine, gauche effect, hyperconjugation

## Abstract

Difluoromethylornithine (DFMO, eflornithine) is a fluorinated analogue of ornithine that serves both as an inhibitor of ornithine decarboxylase and as a therapeutic agent against African trypanosomiasis. Beyond its pharmacological importance, DFMO provides a valuable model for examining how fluorine substitution governs molecular conformation. A comprehensive quantum-chemical study was performed to elucidate the origins of DFMO’s conformational stability. High-level DLPNO-CCSD(T)/CBS calculations revealed that type-**I** conformers – those maximizing gauche interactions between C–F and C–N bonds – dominate the equilibrium population, confirming the presence of the fluorine gauche effect. natural bond orbital (NBO) analysis showed that this preference arises primarily from hyperconjugative stabilization, particularly the σ_CH_ → σ*_CN_ interaction, while steric effects modulate the relative stability among low-energy conformers. The gauche effect is intensified in the zwitterionic form due to electrostatic interactions. In contrast, the bioconformation observed in crystallographic data corresponds to a type-**II** structure, imposed by strong hydrogen bonding of the amino and carboxyl groups with surrounding residues. Thus, DFMO’s intrinsic conformational preferences are dictated by stereoelectronic effects, but these can be overridden by specific intermolecular interactions in biological environments. This study clarifies the electronic origin of DFMO’s gauche effect and provides insight into how local electronic factors determine the structure of fluorinated amino acid derivatives.

## Introduction

**Figure 1:**
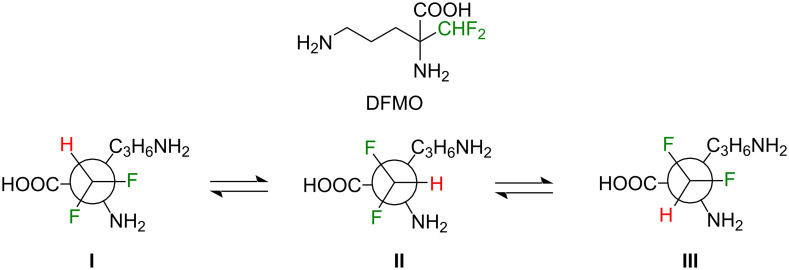
Structure of racemic difluoromethylornithine (DFMO) and conformers **I**–**III** of the (*S*)-enantiomer ((*S*)-DFMO) obtained from rotation around the difluoromethyl group.

Difluoromethylornithine (DFMO, in its racemic form, also known as eflornithine – Figure 1) is a fluorinated analogue of ornithine that has attracted considerable attention due to its dual relevance in medicine and structural chemistry. Clinically, DFMO has been employed in the treatment of African trypanosomiasis (sleeping sickness), particularly the second stage of the disease caused by *Trypanosoma brucei gambiense* [1–3]. It is also used as a topical agent to reduce excessive facial hair growth in women [4–5]. Beyond these applications, DFMO is recognized as a potent inhibitor of ornithine decarboxylase, the key enzyme that catalyzes the first step in polyamine biosynthesis [6]. This inhibition underlies both its therapeutic utility and its importance as a biochemical probe.

From a structural standpoint, DFMO provides an intriguing case study for exploring conformational effects associated with the difluoromethyl (–CHF₂) motif. The presence of vicinal fluorine substituents is known to strongly influence conformational equilibria through stereoelectronic effects, notably the fluorine gauche effect [7]. This effect arises from hyperconjugative interactions (typically σ_CH_ → σ*_CF_) that stabilize conformations in which the C–F bonds adopt a gauche orientation relative to polar bonds, such as C–N or C–O [8–10]. In DFMO, the difluoromethyl group is directly connected to the ornithine backbone, and its conformation is expected to be shaped not only by intrinsic stereoelectronic preferences but also by intermolecular interactions within the ornithine decarboxylase active site.

Therefore, the conformational behavior of DFMO arises from a delicate balance between the fluorine gauche effect and the intermolecular interactions that stabilize the ligand–enzyme complex. Elucidating this interplay is essential for rationalizing DFMO’s inhibitory potency and may also offer broader insight into how fluorinated substituents control molecular conformation in biologically relevant environments. Although the biological milieu is known to induce only minor structural changes in DFMO [11], a systematic evaluation of its zwitterionic and non-zwitterionic forms in the gas phase, aqueous solution, and biological environments is necessary to disentangle the respective contributions of intra- and intermolecular interactions to its conformation and biological activity.

In this work, we present a quantum-chemical study aimed at elucidating the origin of the conformational stability of DFMO. Particular emphasis is placed on natural bond orbital (NBO) analysis [12], which enables a detailed characterization of hyperconjugative interactions and other stereoelectronic effects that contribute to the preferred conformations of the difluoromethyl motif.

## Results and Discussion

**Figure 2:**
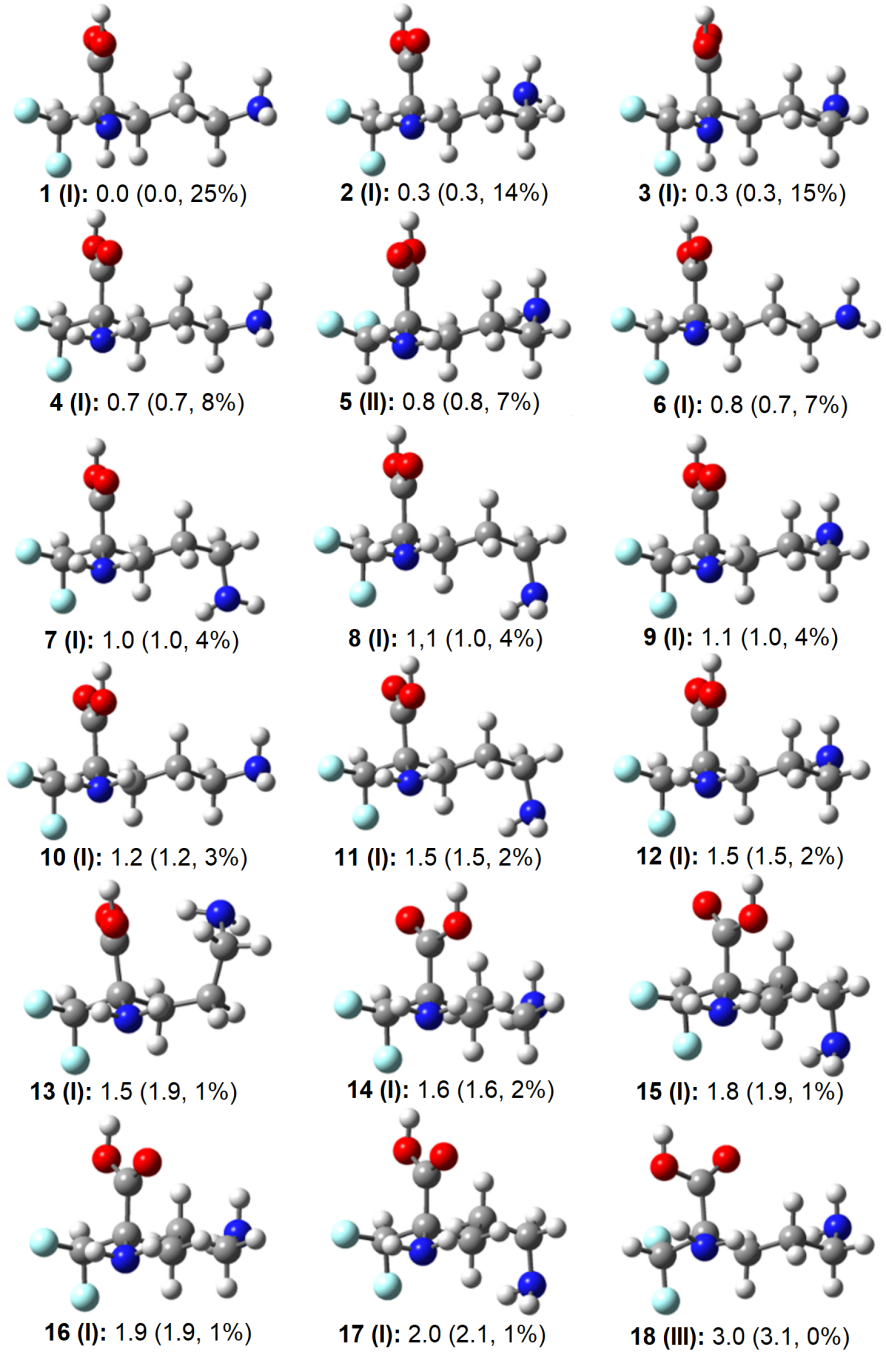
Representative conformations of (*S*)-DFMO with their relative electronic energies (in kcal mol^−1^), standard Gibbs free energies (in parenthesis), and Boltzmann populations calculated at the DLPNO-CCSD(T)/CBS level of theory. Color labels: H = white, C = grey, N = blue, O = red, F = electric blue.

According to Wolfe [7], the gauche effect is defined as “a tendency to adopt that structure which has the maximum number of gauche interactions between adjacent electron pairs and/or polar bonds.” In this framework, DFMO is expected to display the gauche effect when type-**I** structures dominate, because the polar C–N bond forms two gauche interactions with the polar C–F bonds. High-level DLPNO-CCSD(T)/CBS calculations support this expectation: the four lowest-energy structures are type **I** and together account for about 62% of the equilibrium population. By contrast, type-**II** and type-**III** structures contribute only 7% and 0%, respectively (Figure 2). These results demonstrate that DFMO exhibits the gauche effect, unlike 1,1,2-trifluoroethane and 1,1,2,2-tetrafluoroethane, whose preferred geometries minimize gauche C–F interactions [13–18]. It is also noteworthy that the zwitterionic form of DFMO dissociates upon gas-phase optimization, whereas the non-zwitterionic form in implicit water, modeled using the solvation model density (SMD), remains stable in a type-**I** conformation, with conformer **4** being the most stable (14%). Consequently, the gauche effect persists in solution (Figure S1, Supporting Information File 1).

To clarify the origin of this behavior, natural bond orbital (NBO) analyses were carried out to separate steric and electron-delocalization effects. The principal low-energy structures (**1**–**4**) differ mainly by rotations about the C–COOH, C–NH_2_, and C–C(NH_2_) bonds. As a result, their relative energies are governed primarily by the local environments of these groups rather than by the orientation of the difluoromethyl unit. Steric analysis based on exchange energies between natural localized molecular orbitals shows that structure **4** experiences the weakest steric repulsion, whereas structures **1**–**3** are among the most sterically crowded (Table 1). Several interactions contribute to this trend; notably, the *n*_N_/σ_CC_ interaction involving the NH₂ group geminal to the difluoromethyl moiety is significantly less repulsive in structure **4**. In addition, the zigzag conformation of the *N*-alkyl chain reduces σ_CN_/σ_CC_ repulsions.

**Table 1:** DFT electronic energies and their Lewis and non-Lewis components, together with steric energies obtained from natural bond orbital (NBO) analysis (in kcal mol^−^¹).

Conformer	*E*_rel_	*E*_NL_	*E*_L_	*E*_steric_

**1**	0.0	−502.6	502.6	295.4
**2**	0.5	−496.2	496.7	296.3
**3**	0.5	−504.2	504.7	296.3
**4**	0.7	−499.5	500.2	293.2
**5**	1.2	−501.7	502.8	294.0
**6**	0.8	−497.5	498.3	294.3
**7**	1.4	−498.4	499.8	295.0
**8**	1.2	−500.4	501.6	294.0
**9**	1.3	−500.6	501.9	294.0
**10**	1.3	−502.9	504.2	293.8
**11**	1.7	−503.8	505.5	294.8
**12**	1.7	−504.5	506.2	294.9
**13**	3.2	−511.4	514.5	298.5
**14**	1.8	−498.0	499.8	294.1
**15**	2.2	−498.8	500.9	295.1
**16**	1.9	−497.5	499.4	293.9
**17**	2.2	−498.5	500.8	295.1
**18**	3.2	−498.5	501.7	296.3

Structure **5** (type **II**) displays a comparatively low steric penalty (294.0 kcal mol^−1^), much smaller than those of structures **1**–**3**. This reduction arises in part because its C–N bond is not flanked by two C–F bonds. At the other extreme, structure **13** is highly destabilized sterically due to folding of the *N*-alkyl chain, which brings N–H and C–N bonds into close contact with C–H, C–C, and C=O bonds. This geometry generates several *n*/σ and σ/σ interactions of 0.5–1.4 kcal mol^−1^ each (see Supporting Information File 1). Overall, steric effects alone cannot explain the stability ordering, as indicated by the near-zero correlation between relative energy (*E*_rel_) and steric energy (*E*_steric_). Electron delocalization therefore plays a decisive role.

This contribution can be quantified by decomposing *E*_rel_ into Lewis (*E*_L_) and non-Lewis (*E*_NL_) terms. The former includes destabilizing 4-electron/2-orbital interactions, while the latter accounts for stabilizing 2-electron/2-orbital interactions. Although *E*_L_ contains steric effects, its correlation with *E*_steric_ is weak (R^2^ = 0.30). In practice, *E*_rel_ and *E*_NL_ are obtained directly from the NBO analysis, whereas *E*_L_ is determined by difference (Table 1). Structure **13** is the most delocalized but also experiences strong destabilizing interactions. The five least stable structures (**14**–**18**), including the type-**III** geometry, gain less than 500 kcal mol^−1^ from delocalization, whereas the most stable structure (**1**) is stabilized by 502.6 kcal mol^−1^. Notably, structure **5** (type **II**) is also strongly stabilized by delocalization (*E*_NL_ = −501.7 kcal mol^−1^), while structure **2** shows weaker stabilization (−496.2 kcal mol^−1^). These results indicate that the gauche effect in DFMO arises from a balance between strong non-Lewis stabilization and only moderate Lewis-type destabilization.

Further insight was obtained by examining specific hyperconjugative interactions through second-order perturbation analysis. In 1,2-difluoroethane, the classical gauche effect is attributed to σ_CH_ → σ*_CF_ hyperconjugation [8–10,19]. In DFMO, the σ_CH_ orbital of the difluoromethyl group acts as the primary donor, while the vicinal σ*_CN_ orbital is the main acceptor. Additional antiperiplanar interactions within the (F₂H)C–C fragment, which together with Lewis-type effects control the orientation of the difluoromethyl group, are summarized in Table S1 (Supporting Information File 1). As expected, σ_CH_ → σ*_CN_ is the dominant interaction (>3 kcal mol^−1^) and is present in all type-**I** structures. When these interactions are summed for each structure, **12** of the **13** lowest-energy structures show stabilization greater than 11 kcal mol^−1^. Structure **5** is the only exception, yet it remains low in energy due to its reduced steric repulsion. By contrast, the least stable structure (**18**) shows the weakest hyperconjugative stabilization because, as a type-**III** geometry, it exhibits a σ_CH_ → σ*_CC_ interaction (2.4 kcal mol^−1^) instead of the more favorable σ_CH_ → σ*_CN_ interaction.

Overall, the gauche effect in DFMO appears to be driven by hyperconjugation and modulated by steric effects. For instance, conformers **1** and **2** are similarly stabilized by hyperconjugation, but the former experiences weaker steric repulsion. Conversely, conformer **18** is both the least stabilized by hyperconjugation and one of the most sterically hindered.

**Figure 3:**
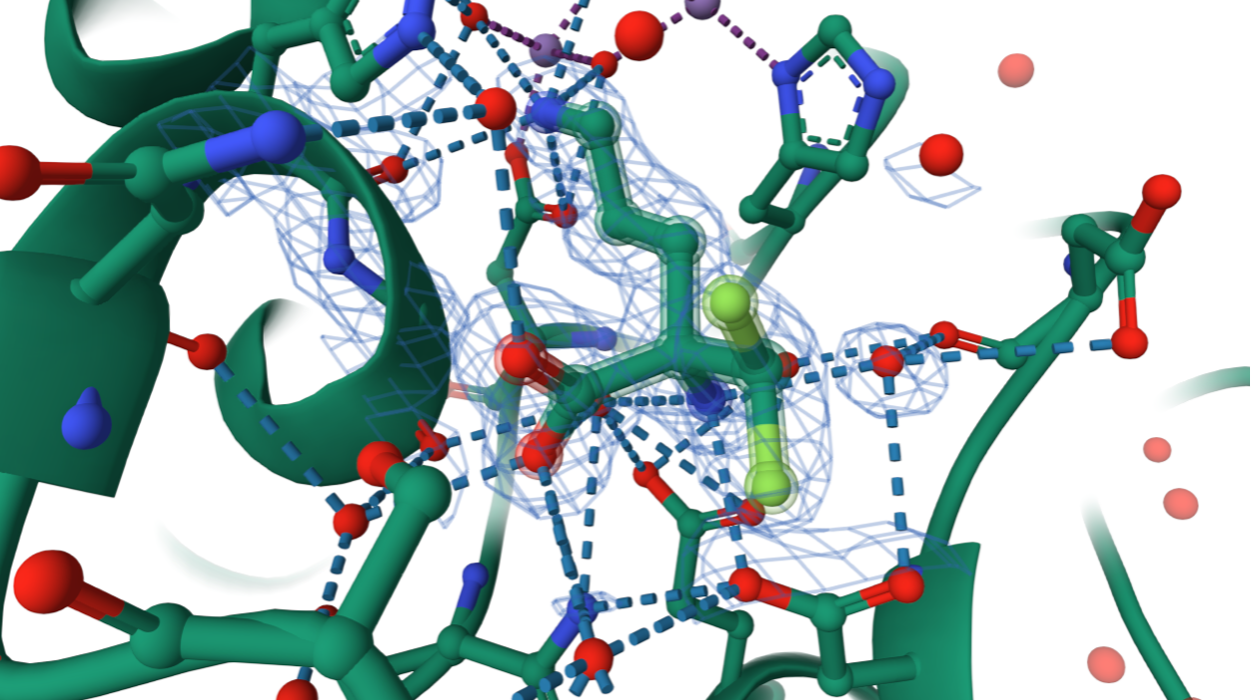
(*S*)-DFMO bound within the active site of human arginase I obtained from the Protein Data Bank (**3GN0**) (https://doi.org/10.2210/pdb3gn0/pdb, [21]). Dotted lines represent hydrogen-bonding interactions with amino acid residues and water molecules, while the ligand is delineated by a contour line to distinguish it from the binding cavity.

The gas-phase preferences can be contrasted with the bioconformation of (*S*)-DFMO. Although PDB entry **9FOS** is reported as ornithine decarboxylase from *Leishmania infantum* complexed with PLP and DFMO, the bound ligand corresponds instead to human arginase I (PDB code **3GN0**). While binding to arginase I is not related to DFMO’s pharmacological activity, both enzymes recognize ornithine-related substrates, allowing DFMO to occupy the arginase active site in crystallographic studies. In this environment, the fluorine atoms do not engage in favorable contacts with residues or water molecules. Instead, the geometry is dictated by interactions involving the NH₂ and COOH groups, yielding a bioconformation similar to arrangement **II**. Specifically, the NH₂ group forms a hydrogen bond with ASP183, and the carboxylate interacts with ASN130 (Figure 3), with additional hydrogen bonds to water molecules. Consequently, intermolecular interactions dominate and override the intramolecular forces responsible for the gauche effect in the isolated molecule. This is consistent with the relative strength of hydrogen bonds (5–40 kcal mol^−1^) compared with typical hyperconjugative interactions [20].

**Figure 4:**
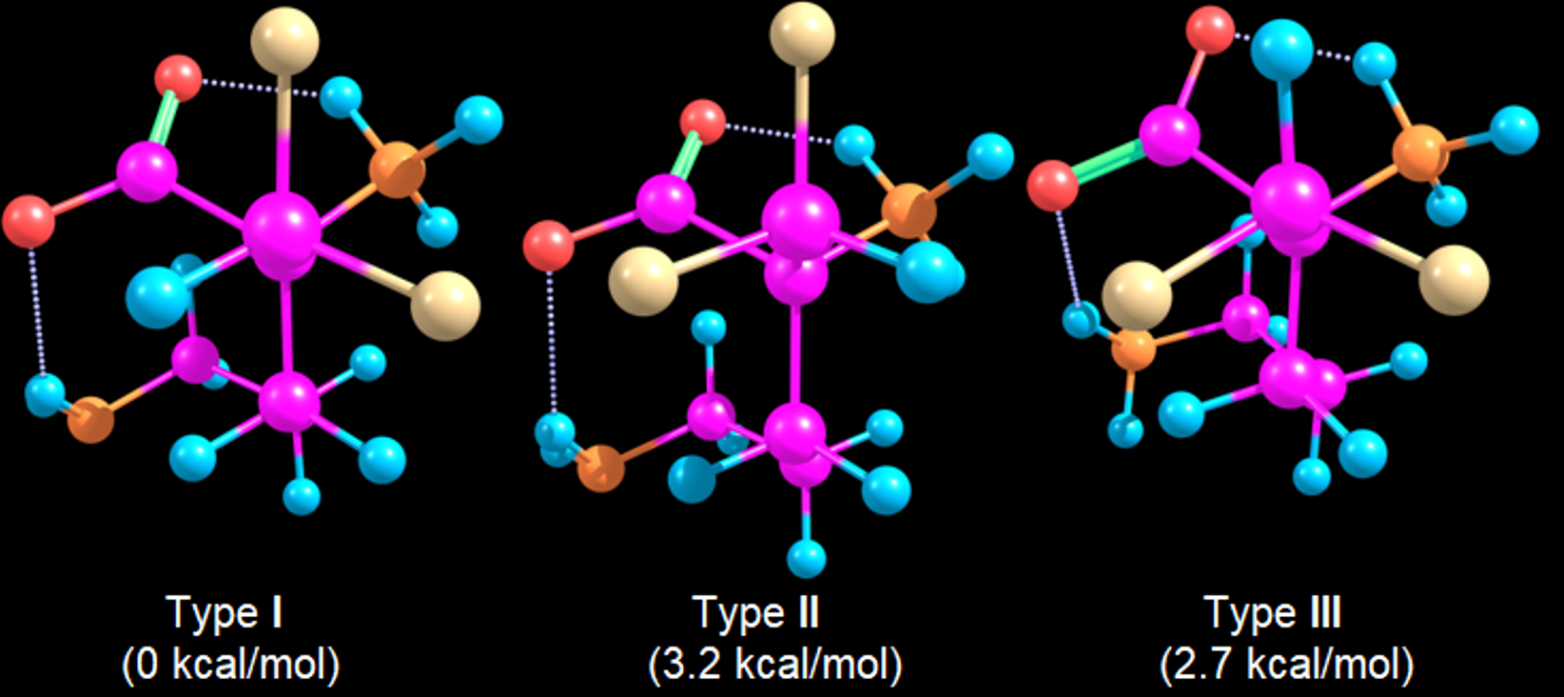
Lowest-energy type-**I**, type-**II**, and type-**III** conformers of the zwitterionic form of (*S*)-DFMO. Color labels: H = blue, C = pink, N = orange, O = red, F = yellow.

Finally, because neutral amino acids dominate in the gas phase whereas zwitterions prevail in solution [21–24], the zwitterionic form of DFMO was examined in implicit water. A conformational search using the global optimizer algorithm at the GFN2-xTB level identified 66 structures within 3 kcal mol^−1^. All but one were type **I** (Figure 4). The first type-**II** and type-**III** geometries lie 3.2 and 2.7 kcal mol^−1^ above the lowest-energy structure, corresponding to negligible Boltzmann populations. Thus, zwitterion formation reinforces the gauche effect, likely through an electrostatic attraction between negatively charged fluorine atoms and the positively charged NH₃^+^ group, as observed in related systems [25]. Even so, this intramolecular stabilization remains insufficient to overcome the stronger intermolecular interactions present in biological environments.

## Conclusion

DFMO exhibits the fluorine gauche effect, as type-**I** conformers – those maximizing gauche interactions between the C–F and C–N bonds – dominate the conformational equilibrium by a large margin. This preference arises primarily from hyperconjugative stabilization, particularly the σ_CH_ → σ*_CN_ interaction, which is absent in type-**II** and type-**III** conformers. This behavior contrasts with that of 1,1,2-trifluoroethane and 1,1,2,2-tetrafluoroethane, which instead favor conformations that minimize gauche interactions between the polar C–F bonds. The relative energies among the type-**I** conformers, however, are further modulated by steric effects. Notably, the type-**II** conformer (**5**) ranks among the most stable structures owing to its minimal steric repulsion. In a biological environment, this balance shifts, since strong intermolecular interactions – especially hydrogen bonding with amino acid residues – favor a type-**II** geometry as the predominant bioconformation. Nevertheless, when different conformers engage in comparable intermolecular interactions, intramolecular effects such as the gauche effect are expected to play a decisive role in determining the bioactive conformation and, consequently, the molecular bioactivity.

## Methods

A stochastic conformational search of (*S*)-DFMO was performed at the semiempirical AM1 level [26] using the Spartan program [27]. The Monte Carlo algorithm employed explores the conformational space by randomly sampling molecular geometries and accepting or rejecting new structures according to their relative energies. Among the 100 generated conformers, 20 exhibited nonzero Boltzmann populations, of which 16 were unique. These 16 conformers (arrangement **I**), along with two additional structures – one with the COOH group (**II**) and another with the C_2_H_4_NH_2_ group (**III**) positioned between the two C–F bonds – were subsequently optimized at the B3LYP-GD3BJ/6-311++G(d,p) level [28–31] of density functional theory. Frequency calculations confirmed that all optimized geometries correspond to true minima. These calculations were performed using the Gaussian 16 program [32]. The optimized structures were then subjected to single-point energy calculations at the DLPNO-CCSD(T)/CBS level [33–34] with the ORCA software package [35] to obtain accurate conformational energies, where DLPNO-CCSD(T) denotes the domain-based local pair natural orbital coupled-cluster method including single, double, and perturbative triple excitations, and the complete basis set (CBS) limit was obtained by extrapolating Hartree–Fock and correlation energies from calculations performed with the cc-pVTZ and cc-pVQZ basis sets. Natural bond orbital (NBO) analyses [12] were also performed at the same DFT level, employing the NBO7DEL, LEWIS, and STERIC keywords to suppress electron-delocalization effects and thereby quantify the contributions of Lewis- and non-Lewis-type interactions to the total electronic energy, as well as to evaluate steric exchange energies from pairwise interactions. Additionally, the zwitterionic structure of DFMO reported by Ilies et al. [21] was investigated through a conformational search using the global optimizer algorithm (GOAT) at the GFN2-xTB(ALPB) semiempirical level [36–37] with an implicit water solvent model, considering that the β-alanine zwitterion predominates in aqueous solution [22], while neutral forms arising from proton transfer are typically observed in the gas phase [23–24].

## Supporting Information

File 1 NBO outcomes, standard orientations and Gibbs free energies for the studied compounds.

## Data Availability

All data that supports the findings of this study is available in the published article and/or the supporting information of this article.
