# Supplementary material for: Conformational analysis of difluoromethylornithine: factors influencing its gas-phase and bioactive conformations
Source: Beilstein J Org Chem. 2026 Feb 5;22:237–43. doi: 10.3762/bjoc.22.17 (PMC12884545; doi:10.3762/bjoc.22.17)
Supplement: File 1 — NBO outcomes, standard orientations and Gibbs free energies for the studied compounds. [file Beilstein_J_Org_Chem-22-237-s001.pdf]

BEILSTEIN JOURNAL OF ORGANIC CHEMISTRY

Supporting Information

for

Conformational analysis of difluoromethylornithine: factors influencing its gas-phase and bioactive conformations

Matheus P. Freitas

Beilstein J. Org. Chem. **2026,** *22,* 237–243. doi:10.3762/bjoc.22.17

NBO outcomes, standard orientations and Gibbs free energies for the studied compounds

License and Terms: This is a supporting information file under the terms of the Creative Commons Attribution License (https://creativecommons.org/licenses/by/4.0). Please note that the reuse, redistribution and reproduction in particular requires that the author(s) and source are credited and that individual graphics may be subject to special legal provisions.
The license is subject to the *Beilstein Journal of Organic Chemistry* terms and conditions: (https://www.beilstein-journals.org/bjoc/terms)

Table of contents

Page S2: **Figure S1.** Representative conformations of (*S*)-DFMO with their relative electronic energies (in kcal mol^{-1}), standard Gibbs free energies (in parenthesis), and Boltzmann populations calculated at the DLPNO-CCSD(T)/CBS level of theory in implicit water (SMD). Color labels: H = white, C = grey, N = blue, O = red, F = electric blue.

Page S3: **Table S1.** Antiperiplanar donor–acceptor interactions obtained from NBO calculations at the B3LYP-GD3BJ/6-311++G(d,p) level (kcal mol^{-1}).

Pages S4–S57: Standard coordinates of the optimized geometries of DFMO conformers obtained at the B3LYP-GD3BJ/6-311++G(d,p) level, absolute energies computed at the DLPNO-CCSD(T)/CBS level (hartrees), and pairwise steric exchange energies dE(*i,j*) (kcal mol^{-1}) with the corresponding pre-NLMO overlaps S(*i,j*) for disjoint (non-overlapping) NLMO pairs *i,j*.

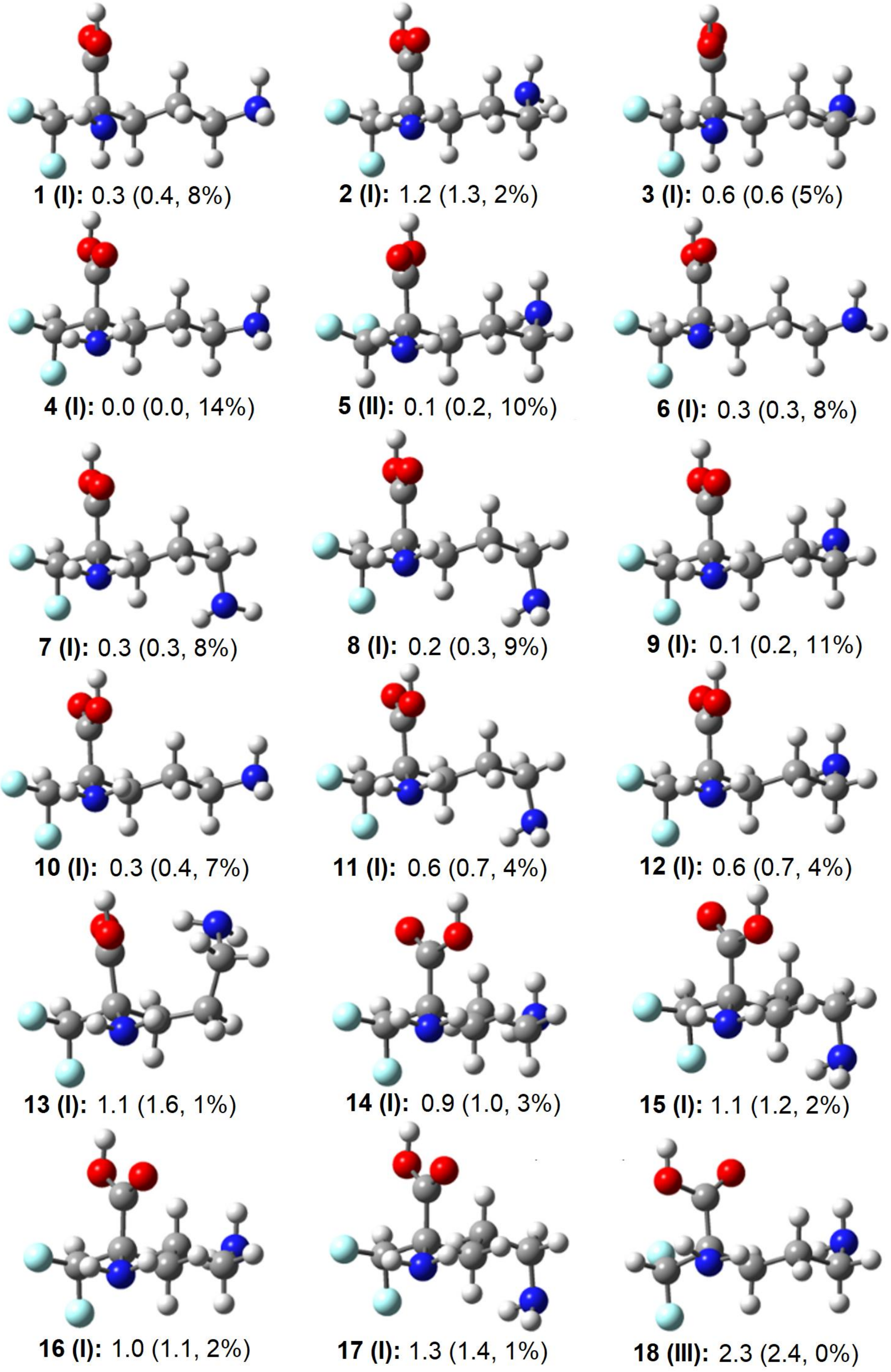

Figure S1. Representative conformations of (*S*)-DFMO with their relative electronic energies (in kcal mol^{-1}), standard Gibbs free energies (in parenthesis), and Boltzmann populations calculated at the DLPNO-CCSD(T)/CBS level of theory in implicit water (SMD). Color labels: H = white, C = grey, N = blue, O = red, F = electric blue.

Table S1. Antiperiplanar donor–acceptor interactions obtained from NBO calculations at the B3LYP-GD3BJ/6-311++G(d,p) level (kcal mol^{-1}).

Conf.	$\sigma_{CH}\rightarrow\sigma^*_{CN}$	$\sigma_{CH}\rightarrow\sigma^*_{CC(OOH)}$	$\sigma_{CH}\rightarrow\sigma^*_{CC3H6NH2}$	$\sigma_{CF}\rightarrow\sigma^*_{CN}$	$\sigma_{CF}\rightarrow\sigma^*_{CC(OOH)}$	$\sigma_{CF}\rightarrow\sigma^*_{CC3H6NH2}$	$\sigma_{CN}\rightarrow\sigma^*_{CH}$	$\sigma_{CN}\rightarrow\sigma^*_{CF}$	$\sigma_{CC(OOH)}\rightarrow\sigma^*_{CH}$	$\sigma_{CC(OOH)}\rightarrow\sigma^*_{CF}$	$\sigma_{CC3H6NH2}\rightarrow\sigma^*_{CH}$	$\sigma_{CC3H6NH2}\rightarrow\sigma^*_{CF}$	Sum
**1**	3.50				1.01	1.13	0.83			2.58		2.40	11.45
**2**	3.25				1.08	1.11	0.99			2.34		2.57	11.34
**3**	3.48				1.00	1.14	0.83			2.60		2.40	11.45
**4**	3.23				1.09	1.23	0.99			2.36		2.52	11.42
**5**		2.59		1.18		1.18		2.12	1.48			2.33	10.88
**6**	3.19				1.07	1.23	0.99			2.38		2.50	11.36
**7**	3.16				1.06	1.23	0.99			2.38		2.46	11.28
**8**	3.22				1.08	1.24	0.99			2.37		2.53	11.43
**9**	3.21				1.08	1.24	0.99			2.37		2.52	11.41
**10**	3.18				0.95	1.23	0.93			2.72		2.42	11.43
**11**	3.17				0.95	1.24	0.93			2.72		2.42	11.43
**12**	3.17				0.95	1.24	0.93			2.73		2.42	11.44
**13**	3.13				0.91	1.21	0.93			2.87		2.41	11.46
**14**	3.08				0.96	1.19	0.95			2.37		2.21	10.76
**15**	3.07				0.96	1.20	0.95			2.36		2.19	10.73
**16**	3.11				1.12	1.20	1.05			2.05		2.38	10.91
**17**	3.10				1.12	1.21	1.05			2.06		2.38	10.92
**18**			2.38	1.40	0.94			1.77		2.27	1.28		10.04

Conformer **1**

Standard orientation:

Center Atomic Atomic Coordinates (Angstroms)

Number Number Type X Y Z

----

1 6 0 -0.660461 -0.106435 0.193952

2 6 0 -0.748975 1.353889 -0.262234

3 8 0 -0.696842 1.680100 -1.422144

4 8 0 -0.911550 2.228845 0.744298

5 1 0 -0.983494 3.110808 0.347600

6 7 0 -0.706008 -0.231543 1.640495

7 1 0 -0.778773 -1.209873 1.899401

8 1 0 -1.498175 0.262616 2.034697

9 6 0 -1.845514 -0.812519 -0.502472

10 1 0 -1.838234 -0.721327 -1.588418

11 9 0 -1.837393 -2.144818 -0.174492

12 9 0 -3.031395 -0.298447 -0.035889

13 6 0 0.655137 -0.711580 -0.346922

14 1 0 0.617020 -0.712169 -1.439880

15 1 0 0.685346 -1.757128 -0.025135

16 6 0 1.910847 0.018490 0.131303

17 1 0 1.886368 0.104368 1.221120

18 1 0 1.922439 1.038566 -0.270678

19 6 0 3.195649 -0.698083 -0.305072

20 1 0 3.195537 -1.717945 0.095019

21 1 0 3.208705 -0.798242 -1.395758

22 7 0 4.449601 -0.061211 0.098303

23 1 0 4.524441 0.872608 -0.292627

24 1 0 4.499800 0.032247 1.107933

E [DLPNO-CCSD(T)/CBS] = -694.8702753

Pairwise steric exchange energies dE(i,j) (kcal/mol) and associated pre-NLMO overlaps S(i,j) for disjoint (no common atoms) interactions between NLMOs i,j:

Threshold for printing: 0.50 kcal/mol
NLMO (i) NLMO (j) S(i,j) kcal/mol
===
within unit 1
13. LP (1) O 3 25. BD (1) C 1- C 2 -0.0400 0.60
13. LP (1) O 3 31. BD (1) C 2- O 4 -0.0414 0.71
14. LP (2) O 3 15. LP (1) O 4 -0.0178 -0.62
14. LP (2) O 3 25. BD (1) C 1- C 2 0.1766 12.36
14. LP (2) O 3 27. BD (1) C 1- C 9 0.1110 0.57
14. LP (2) O 3 28. BD (1) C 1- C 13 0.1198 0.99
14. LP (2) O 3 31. BD (1) C 2- O 4 -0.2041 13.07
14. LP (2) O 3 32. BD (1) O 4- H 5 -0.1348 2.28
14. LP (2) O 3 35. BD (1) C 9- H 10 0.0840 0.95
14. LP (2) O 3 38. BD (1) C 13- H 14 0.0931 1.06
15. LP (1) O 4 17. LP (1) N 6 0.0553 0.57
15. LP (1) O 4 25. BD (1) C 1- C 2 0.0948 2.91
15. LP (1) O 4 26. BD (1) C 1- N 6 0.0715 0.61
15. LP (1) O 4 29. BD (1) C 2- O 3 -0.1308 4.65
15. LP (1) O 4 34. BD (1) N 6- H 8 0.0880 1.55
16. LP (2) O 4 27. BD (1) C 1- C 9 -0.0712 0.85
16. LP (2) O 4 28. BD (1) C 1- C 13 0.0679 0.80
16. LP (2) O 4 30. BD (2) C 2- O 3 -0.1963 14.44
17. LP (1) N 6 25. BD (1) C 1- C 2 0.0647 0.69
17. LP (1) N 6 27. BD (1) C 1- C 9 -0.2169 12.27
17. LP (1) N 6 28. BD (1) C 1- C 13 0.1134 3.05
17. LP (1) N 6 40. BD (1) C 13- C 16 0.0977 0.58
17. LP (1) N 6 41. BD (1) C 16- H 17 0.1124 2.02
18. LP (1) F 11 23. LP (3) F 12 -0.0607 -0.63
19. LP (2) F 11 27. BD (1) C 1- C 9 -0.1542 8.33
19. LP (2) F 11 33. BD (1) N 6- H 7 -0.0617 0.63
19. LP (2) F 11 35. BD (1) C 9- H 10 0.1116 7.03
20. LP (3) F 11 21. LP (1) F 12 -0.0594 -0.60
20. LP (3) F 11 23. LP (3) F 12 0.0576 1.76
20. LP (3) F 11 27. BD (1) C 1- C 9 0.0734 1.88
20. LP (3) F 11 28. BD (1) C 1- C 13 0.0785 0.81
20. LP (3) F 11 35. BD (1) C 9- H 10 0.0547 1.42
20. LP (3) F 11 37. BD (1) C 9- F 12 -0.1435 6.99
20. LP (3) F 11 39. BD (1) C 13- H 15 0.0581 0.78
22. LP (2) F 12 27. BD (1) C 1- C 9 -0.1570 8.65
22. LP (2) F 12 35. BD (1) C 9- H 10 0.1106 6.80
23. LP (3) F 12 25. BD (1) C 1- C 2 0.0815 0.97
23. LP (3) F 12 27. BD (1) C 1- C 9 0.0734 1.82
23. LP (3) F 12 35. BD (1) C 9- H 10 0.0606 1.71
23. LP (3) F 12 36. BD (1) C 9- F 11 -0.1439 6.95
24. LP (1) N 22 41. BD (1) C 16- H 17 -0.0894 0.62
24. LP (1) N 22 42. BD (1) C 16- H 18 -0.0917 0.68
24. LP (1) N 22 43. BD (1) C 16- C 19 -0.2232 12.04
24. LP (1) N 22 44. BD (1) C 19- H 20 0.0666 1.66
24. LP (1) N 22 45. BD (1) C 19- H 21 0.0632 1.49
25. BD (1) C 1- C 2 32. BD (1) O 4- H 5 -0.1003 4.23
25. BD (1) C 1- C 2 33. BD (1) N 6- H 7 -0.1242 5.34
25. BD (1) C 1- C 2 34. BD (1) N 6- H 8 0.1029 2.19
25. BD (1) C 1- C 2 35. BD (1) C 9- H 10 0.0840 1.19
25. BD (1) C 1- C 2 36. BD (1) C 9- F 11 -0.1110 3.35
25. BD (1) C 1- C 2 37. BD (1) C 9- F 12 0.0594 0.60
25. BD (1) C 1- C 2 38. BD (1) C 13- H 14 0.0718 0.76
25. BD (1) C 1- C 2 39. BD (1) C 13- H 15 -0.1317 4.85
25. BD (1) C 1- C 2 40. BD (1) C 13- C 16 0.0888 1.61
25. BD (1) C 1- C 2 42. BD (1) C 16- H 18 0.0638 0.56
26. BD (1) C 1- N 6 29. BD (1) C 2- O 3 -0.1074 2.02
26. BD (1) C 1- N 6 31. BD (1) C 2- O 4 0.0892 1.72
26. BD (1) C 1- N 6 35. BD (1) C 9- H 10 -0.1034 2.92
26. BD (1) C 1- N 6 37. BD (1) C 9- F 12 0.0531 0.52
26. BD (1) C 1- N 6 38. BD (1) C 13- H 14 -0.1334 4.47
26. BD (1) C 1- N 6 39. BD (1) C 13- H 15 0.0826 1.33
26. BD (1) C 1- N 6 40. BD (1) C 13- C 16 0.0556 0.64
27. BD (1) C 1- C 9 30. BD (2) C 2- O 3 0.1277 3.86
27. BD (1) C 1- C 9 31. BD (1) C 2- O 4 -0.0613 0.94
27. BD (1) C 1- C 9 33. BD (1) N 6- H 7 0.0773 1.37
27. BD (1) C 1- C 9 38. BD (1) C 13- H 14 0.1039 2.23
27. BD (1) C 1- C 9 39. BD (1) C 13- H 15 0.0778 1.37
27. BD (1) C 1- C 9 40. BD (1) C 13- C 16 -0.1469 4.74

28. BD (1) C 1- C 13 29. BD (1) C 2- O 3 0.0541 0.80
28. BD (1) C 1- C 13 30. BD (2) C 2- O 3 -0.1148 3.08
28. BD (1) C 1- C 13 31. BD (1) C 2- O 4 -0.0650 0.91
28. BD (1) C 1- C 13 33. BD (1) N 6- H 7 0.0700 1.25
28. BD (1) C 1- C 13 34. BD (1) N 6- H 8 -0.1279 5.25
28. BD (1) C 1- C 13 35. BD (1) C 9- H 10 0.0725 1.10
28. BD (1) C 1- C 13 36. BD (1) C 9- F 11 0.0531 0.62
28. BD (1) C 1- C 13 37. BD (1) C 9- F 12 -0.1136 3.35
28. BD (1) C 1- C 13 41. BD (1) C 16- H 17 0.0849 1.63
28. BD (1) C 1- C 13 42. BD (1) C 16- H 18 0.0643 0.91
28. BD (1) C 1- C 13 43. BD (1) C 16- C 19 -0.1446 4.22
29. BD (1) C 2- O 3 32. BD (1) O 4- H 5 0.0819 2.09
30. BD (2) C 2- O 3 35. BD (1) C 9- H 10 0.0586 0.59
30. BD (2) C 2- O 3 42. BD (1) C 16- H 18 -0.0812 1.08
33. BD (1) N 6- H 7 39. BD (1) C 13- H 15 0.0756 0.64
35. BD (1) C 9- H 10 38. BD (1) C 13- H 14 0.0792 0.96
38. BD (1) C 13- H 14 41. BD (1) C 16- H 17 -0.1264 5.38
38. BD (1) C 13- H 14 42. BD (1) C 16- H 18 0.0871 1.49
38. BD (1) C 13- H 14 43. BD (1) C 16- C 19 0.0673 0.97
38. BD (1) C 13- H 14 45. BD (1) C 19- H 21 0.0653 0.62
39. BD (1) C 13- H 15 41. BD (1) C 16- H 17 0.0667 0.68
39. BD (1) C 13- H 15 42. BD (1) C 16- H 18 -0.1245 5.30
39. BD (1) C 13- H 15 43. BD (1) C 16- C 19 0.0902 1.81
39. BD (1) C 13- H 15 44. BD (1) C 19- H 20 0.0760 0.85
40. BD (1) C 13- C 16 44. BD (1) C 19- H 20 0.0749 1.21
40. BD (1) C 13- C 16 45. BD (1) C 19- H 21 0.0751 1.25
40. BD (1) C 13- C 16 46. BD (1) C 19- N 22 -0.1287 3.41
41. BD (1) C 16- H 17 44. BD (1) C 19- H 20 0.0837 1.28
41. BD (1) C 16- H 17 45. BD (1) C 19- H 21 -0.1149 4.85
41. BD (1) C 16- H 17 46. BD (1) C 19- N 22 0.0649 0.85
42. BD (1) C 16- H 18 44. BD (1) C 19- H 20 -0.1142 4.80
42. BD (1) C 16- H 18 45. BD (1) C 19- H 21 0.0792 1.15
42. BD (1) C 16- H 18 46. BD (1) C 19- N 22 0.0657 0.88
43. BD (1) C 16- C 19 47. BD (1) N 22- H 23 0.0533 0.69
43. BD (1) C 16- C 19 48. BD (1) N 22- H 24 0.0565 0.79
44. BD (1) C 19- H 20 47. BD (1) N 22- H 23 -0.1096 5.16
44. BD (1) C 19- H 20 48. BD (1) N 22- H 24 0.0751 1.31
45. BD (1) C 19- H 21 47. BD (1) N 22- H 23 0.0789 1.47
45. BD (1) C 19- H 21 48. BD (1) N 22- H 24 -0.1092 5.16
sum within unit 1: 295.40

Total disjoint NLMO steric exchange energy from pairwise sum: 295.40

Conformer **2**

Standard orientation:

Center Atomic Atomic Coordinates (Angstroms)

Number Number Type X Y Z

----

1 6 0 -0.648903 -0.099504 0.294348

2 6 0 -0.644215 1.410053 -0.033250

3 8 0 -0.669777 2.287472 0.789009

4 8 0 -0.558544 1.651085 -1.362558

5 1 0 -0.543066 2.612486 -1.485593

6 7 0 -1.004387 -0.358886 1.677385

7 1 0 -1.972939 -0.104446 1.844681

8 1 0 -0.445309 0.224700 2.289537

9 6 0 -1.676691 -0.779188 -0.621494

10 1 0 -1.489824 -0.595696 -1.678345

11 9 0 -1.704907 -2.126493 -0.415280

12 9 0 -2.938544 -0.303534 -0.326856

13 6 0 0.750631 -0.672101 -0.030819

14 1 0 0.974224 -0.514414 -1.087727

15 1 0 0.695459 -1.748257 0.147001

16 6 0 1.881043 -0.076060 0.809935

17 1 0 1.737092 -0.325687 1.865466

18 1 0 1.877236 1.019078 0.743446

19 6 0 3.255012 -0.589670 0.373178

20 1 0 4.005378 -0.265988 1.109419

21 1 0 3.250369 -1.684239 0.385127

22 7 0 3.560911 -0.159406 -0.996869

23 1 0 4.386649 -0.627429 -1.353099

24 1 0 3.745422 0.838542 -1.027312

E [DLPNO-CCSD(T)/CBS] = -694.8698403

Pairwise steric exchange energies dE(i,j) (kcal/mol) and associated pre-NLMO overlaps S(i,j) for disjoint (no common atoms) interactions between NLMOs i,j:

Threshold for printing: 0.50 kcal/mol
NLMO (i) NLMO (j) S(i,j) kcal/mol
===
within unit 1
13. LP (1) O 3 25. BD (1) C 1- C 2 -0.0511 0.78
13. LP (1) O 3 31. BD (1) C 2- O 4 -0.0449 0.80
14. LP (2) O 3 15. LP (1) O 4 0.0160 -0.55
14. LP (2) O 3 17. LP (1) N 6 0.0636 0.57
14. LP (2) O 3 25. BD (1) C 1- C 2 -0.1658 11.62
14. LP (2) O 3 26. BD (1) C 1- N 6 -0.1266 1.55
14. LP (2) O 3 31. BD (1) C 2- O 4 0.2081 13.40
14. LP (2) O 3 32. BD (1) O 4- H 5 0.1369 2.31
14. LP (2) O 3 34. BD (1) N 6- H 8 -0.0913 1.22
15. LP (1) O 4 25. BD (1) C 1- C 2 0.1095 3.38
15. LP (1) O 4 29. BD (1) C 2- O 3 -0.1267 4.50
15. LP (1) O 4 35. BD (1) C 9- H 10 0.0953 1.73
15. LP (1) O 4 38. BD (1) C 13- H 14 0.0669 0.71
16. LP (2) O 4 27. BD (1) C 1- C 9 -0.0665 0.86
16. LP (2) O 4 28. BD (1) C 1- C 13 0.0920 1.54
16. LP (2) O 4 30. BD (2) C 2- O 3 0.1956 14.02
16. LP (2) O 4 38. BD (1) C 13- H 14 0.0610 0.65
17. LP (1) N 6 19. LP (2) F 11 -0.0542 0.59
17. LP (1) N 6 25. BD (1) C 1- C 2 -0.1972 11.01
17. LP (1) N 6 27. BD (1) C 1- C 9 0.0621 0.70
17. LP (1) N 6 28. BD (1) C 1- C 13 0.1099 2.95
17. LP (1) N 6 29. BD (1) C 2- O 3 -0.0810 0.76
17. LP (1) N 6 39. BD (1) C 13- H 15 0.1136 1.05
18. LP (1) F 11 23. LP (3) F 12 -0.0598 -0.62
19. LP (2) F 11 27. BD (1) C 1- C 9 -0.1495 7.78
19. LP (2) F 11 35. BD (1) C 9- H 10 0.1171 7.65
20. LP (3) F 11 21. LP (1) F 12 -0.0605 -0.64
20. LP (3) F 11 23. LP (3) F 12 0.0574 1.74
20. LP (3) F 11 27. BD (1) C 1- C 9 0.0714 1.81
20. LP (3) F 11 28. BD (1) C 1- C 13 0.0803 0.80
20. LP (3) F 11 35. BD (1) C 9- H 10 0.0528 1.38
20. LP (3) F 11 37. BD (1) C 9- F 12 -0.1452 7.20
20. LP (3) F 11 39. BD (1) C 13- H 15 0.0651 0.96
22. LP (2) F 12 26. BD (1) C 1- N 6 -0.0798 0.51
22. LP (2) F 12 27. BD (1) C 1- C 9 -0.1544 8.30
22. LP (2) F 12 33. BD (1) N 6- H 7 -0.0758 1.07
22. LP (2) F 12 35. BD (1) C 9- H 10 0.1101 6.63
23. LP (3) F 12 25. BD (1) C 1- C 2 0.0814 0.97
23. LP (3) F 12 27. BD (1) C 1- C 9 0.0699 1.67
23. LP (3) F 12 35. BD (1) C 9- H 10 0.0623 1.83
23. LP (3) F 12 36. BD (1) C 9- F 11 -0.1409 6.69
24. LP (1) N 22 38. BD (1) C 13- H 14 0.1132 2.12
24. LP (1) N 22 40. BD (1) C 13- C 16 0.0891 0.50
24. LP (1) N 22 43. BD (1) C 16- C 19 0.1132 2.99
24. LP (1) N 22 44. BD (1) C 19- H 20 -0.1800 11.28
24. LP (1) N 22 45. BD (1) C 19- H 21 0.0616 0.78
25. BD (1) C 1- C 2 32. BD (1) O 4- H 5 -0.0932 3.83
25. BD (1) C 1- C 2 33. BD (1) N 6- H 7 0.0567 0.50
25. BD (1) C 1- C 2 34. BD (1) N 6- H 8 0.0879 1.57
25. BD (1) C 1- C 2 35. BD (1) C 9- H 10 0.0893 1.29
25. BD (1) C 1- C 2 36. BD (1) C 9- F 11 -0.1141 3.38
25. BD (1) C 1- C 2 37. BD (1) C 9- F 12 0.0566 0.51
25. BD (1) C 1- C 2 38. BD (1) C 13- H 14 0.0930 1.27
25. BD (1) C 1- C 2 39. BD (1) C 13- H 15 -0.1313 4.86
25. BD (1) C 1- C 2 40. BD (1) C 13- C 16 0.0794 1.19
25. BD (1) C 1- C 2 42. BD (1) C 16- H 18 0.0685 0.59
26. BD (1) C 1- N 6 29. BD (1) C 2- O 3 0.0652 1.00
26. BD (1) C 1- N 6 31. BD (1) C 2- O 4 -0.1086 2.48
26. BD (1) C 1- N 6 35. BD (1) C 9- H 10 -0.1131 3.46
26. BD (1) C 1- N 6 37. BD (1) C 9- F 12 0.0569 0.66
26. BD (1) C 1- N 6 38. BD (1) C 13- H 14 -0.1347 4.59
26. BD (1) C 1- N 6 39. BD (1) C 13- H 15 0.0690 0.92
26. BD (1) C 1- N 6 40. BD (1) C 13- C 16 0.0645 0.93
27. BD (1) C 1- C 9 29. BD (1) C 2- O 3 -0.0799 1.17
27. BD (1) C 1- C 9 30. BD (2) C 2- O 3 -0.0956 2.06
27. BD (1) C 1- C 9 31. BD (1) C 2- O 4 0.0715 0.92
27. BD (1) C 1- C 9 33. BD (1) N 6- H 7 0.1005 2.48
27. BD (1) C 1- C 9 34. BD (1) N 6- H 8 -0.1380 6.10

27. BD (1) C 1- C 9 38. BD (1) C 13- H 14 0.0895 1.70
27. BD (1) C 1- C 9 39. BD (1) C 13- H 15 0.0918 1.95
27. BD (1) C 1- C 9 40. BD (1) C 13- C 16 -0.1509 4.80
28. BD (1) C 1- C 13 30. BD (2) C 2- O 3 0.1283 3.66
28. BD (1) C 1- C 13 33. BD (1) N 6- H 7 -0.1297 5.38
28. BD (1) C 1- C 13 34. BD (1) N 6- H 8 0.0663 1.02
28. BD (1) C 1- C 13 35. BD (1) C 9- H 10 0.0666 0.98
28. BD (1) C 1- C 13 36. BD (1) C 9- F 11 0.0501 0.56
28. BD (1) C 1- C 13 37. BD (1) C 9- F 12 -0.1163 3.54
28. BD (1) C 1- C 13 41. BD (1) C 16- H 17 0.0603 0.77
28. BD (1) C 1- C 13 42. BD (1) C 16- H 18 0.0889 1.72
28. BD (1) C 1- C 13 43. BD (1) C 16- C 19 -0.1436 4.17
29. BD (1) C 2- O 3 32. BD (1) O 4- H 5 0.0798 1.91
30. BD (2) C 2- O 3 42. BD (1) C 16- H 18 0.0841 1.25
34. BD (1) N 6- H 8 41. BD (1) C 16- H 17 0.0635 0.97
35. BD (1) C 9- H 10 38. BD (1) C 13- H 14 0.0769 0.81
38. BD (1) C 13- H 14 41. BD (1) C 16- H 17 -0.1248 5.32
38. BD (1) C 13- H 14 42. BD (1) C 16- H 18 0.0657 0.64
38. BD (1) C 13- H 14 43. BD (1) C 16- C 19 0.0939 1.99
39. BD (1) C 13- H 15 41. BD (1) C 16- H 17 0.0921 1.69
39. BD (1) C 13- H 15 42. BD (1) C 16- H 18 -0.1234 5.27
39. BD (1) C 13- H 15 43. BD (1) C 16- C 19 0.0664 1.01
39. BD (1) C 13- H 15 45. BD (1) C 19- H 21 0.0728 0.72
40. BD (1) C 13- C 16 44. BD (1) C 19- H 20 -0.1339 4.70
40. BD (1) C 13- C 16 45. BD (1) C 19- H 21 0.0845 1.56
40. BD (1) C 13- C 16 46. BD (1) C 19- N 22 0.0545 0.61
41. BD (1) C 16- H 17 44. BD (1) C 19- H 20 0.0933 2.12
41. BD (1) C 16- H 17 45. BD (1) C 19- H 21 0.0622 0.71
41. BD (1) C 16- H 17 46. BD (1) C 19- N 22 -0.1314 4.52
42. BD (1) C 16- H 18 44. BD (1) C 19- H 20 0.0707 1.02
42. BD (1) C 16- H 18 45. BD (1) C 19- H 21 -0.1155 4.86
42. BD (1) C 16- H 18 46. BD (1) C 19- N 22 0.0767 1.21
43. BD (1) C 16- C 19 47. BD (1) N 22- H 23 -0.1210 4.52
43. BD (1) C 16- C 19 48. BD (1) N 22- H 24 0.0537 0.75
44. BD (1) C 19- H 20 48. BD (1) N 22- H 24 0.0760 1.34
45. BD (1) C 19- H 21 47. BD (1) N 22- H 23 0.0958 2.58
45. BD (1) C 19- H 21 48. BD (1) N 22- H 24 -0.1065 4.98
sum within unit 1: 296.31

Total disjoint NLMO steric exchange energy from pairwise sum: 296.31

Conformer **3**

Standard orientation:

Center Atomic Atomic Coordinates (Angstroms)

Number Number Type X Y Z

----

1 6 0 -0.621429 -0.083360 0.260911

2 6 0 -0.750277 1.334207 -0.307613

3 8 0 -0.499750 1.602034 -1.456710

4 8 0 -1.188631 2.239054 0.582997

5 1 0 -1.261486 3.086816 0.117838

6 7 0 -0.930760 -0.134713 1.678910

7 1 0 -1.826325 0.293439 1.883302

8 1 0 -0.958811 -1.099661 1.991160

9 6 0 -1.585220 -0.940294 -0.590793

10 1 0 -1.379385 -0.905512 -1.660468

11 9 0 -1.521715 -2.246347 -0.174744

12 9 0 -2.878904 -0.524067 -0.388686

13 6 0 0.820924 -0.580878 0.013942

14 1 0 0.985264 -0.629944 -1.065599

15 1 0 0.882577 -1.603621 0.399596

16 6 0 1.891855 0.287865 0.673490

17 1 0 1.662662 0.390645 1.737596

18 1 0 1.865986 1.296707 0.243661

19 6 0 3.309869 -0.280208 0.509290

20 1 0 4.003900 0.340201 1.084206

21 1 0 3.355562 -1.280592 0.953556

22 7 0 3.830054 -0.378528 -0.855702

23 1 0 3.297250 -1.034834 -1.415346

24 1 0 3.796034 0.520066 -1.326747

E [DLPNO-CCSD(T)/CBS] = -694.8698046

Pairwise steric exchange energies dE(i,j) (kcal/mol) and associated pre-NLMO overlaps S(i,j) for disjoint (no common atoms) interactions between NLMOs i,j:

Threshold for printing: 0.50 kcal/mol
NLMO (i) NLMO (j) S(i,j) kcal/mol
===
within unit 1
13. LP (1) O 3 25. BD (1) C 1- C 2 -0.0399 0.60
13. LP (1) O 3 31. BD (1) C 2- O 4 -0.0413 0.71
14. LP (2) O 3 15. LP (1) O 4 -0.0177 -0.62
14. LP (2) O 3 25. BD (1) C 1- C 2 0.1765 12.35
14. LP (2) O 3 27. BD (1) C 1- C 9 0.1097 0.54
14. LP (2) O 3 28. BD (1) C 1- C 13 0.1219 1.03
14. LP (2) O 3 31. BD (1) C 2- O 4 -0.2041 13.06
14. LP (2) O 3 32. BD (1) O 4- H 5 -0.1346 2.27
14. LP (2) O 3 35. BD (1) C 9- H 10 0.0838 0.94
14. LP (2) O 3 38. BD (1) C 13- H 14 0.0960 1.13
15. LP (1) O 4 17. LP (1) N 6 0.0548 0.56
15. LP (1) O 4 25. BD (1) C 1- C 2 0.0947 2.92
15. LP (1) O 4 26. BD (1) C 1- N 6 0.0714 0.60
15. LP (1) O 4 29. BD (1) C 2- O 3 -0.1311 4.65
15. LP (1) O 4 33. BD (1) N 6- H 7 0.0879 1.55
16. LP (2) O 4 27. BD (1) C 1- C 9 -0.0721 0.87
16. LP (2) O 4 28. BD (1) C 1- C 13 0.0670 0.78
16. LP (2) O 4 30. BD (2) C 2- O 3 -0.1962 14.43
17. LP (1) N 6 25. BD (1) C 1- C 2 0.0654 0.72
17. LP (1) N 6 27. BD (1) C 1- C 9 -0.2185 12.41
17. LP (1) N 6 28. BD (1) C 1- C 13 0.1107 2.91
17. LP (1) N 6 40. BD (1) C 13- C 16 0.0952 0.56
17. LP (1) N 6 41. BD (1) C 16- H 17 0.1110 1.99
18. LP (1) F 11 23. LP (3) F 12 -0.0607 -0.63
19. LP (2) F 11 27. BD (1) C 1- C 9 -0.1539 8.31
19. LP (2) F 11 34. BD (1) N 6- H 8 -0.0613 0.62
19. LP (2) F 11 35. BD (1) C 9- H 10 0.1116 7.02
20. LP (3) F 11 21. LP (1) F 12 -0.0593 -0.60
20. LP (3) F 11 23. LP (3) F 12 0.0577 1.76
20. LP (3) F 11 27. BD (1) C 1- C 9 0.0729 1.86
20. LP (3) F 11 28. BD (1) C 1- C 13 0.0791 0.83
20. LP (3) F 11 35. BD (1) C 9- H 10 0.0551 1.44
20. LP (3) F 11 37. BD (1) C 9- F 12 -0.1432 6.98
20. LP (3) F 11 39. BD (1) C 13- H 15 0.0591 0.80
22. LP (2) F 12 27. BD (1) C 1- C 9 -0.1572 8.69
22. LP (2) F 12 35. BD (1) C 9- H 10 0.1107 6.81
23. LP (3) F 12 25. BD (1) C 1- C 2 0.0814 0.97
23. LP (3) F 12 27. BD (1) C 1- C 9 0.0736 1.82
23. LP (3) F 12 35. BD (1) C 9- H 10 0.0608 1.72
23. LP (3) F 12 36. BD (1) C 9- F 11 -0.1439 6.95
24. LP (1) N 22 38. BD (1) C 13- H 14 -0.0821 0.70
24. LP (1) N 22 42. BD (1) C 16- H 18 -0.0960 0.55
24. LP (1) N 22 43. BD (1) C 16- C 19 -0.2265 12.00
24. LP (1) N 22 44. BD (1) C 19- H 20 0.0766 2.21
24. LP (1) N 22 45. BD (1) C 19- H 21 0.0480 1.01
25. BD (1) C 1- C 2 32. BD (1) O 4- H 5 -0.0998 4.20
25. BD (1) C 1- C 2 33. BD (1) N 6- H 7 0.1026 2.17
25. BD (1) C 1- C 2 34. BD (1) N 6- H 8 -0.1243 5.35
25. BD (1) C 1- C 2 35. BD (1) C 9- H 10 0.0844 1.20
25. BD (1) C 1- C 2 36. BD (1) C 9- F 11 -0.1111 3.35
25. BD (1) C 1- C 2 37. BD (1) C 9- F 12 0.0597 0.60
25. BD (1) C 1- C 2 38. BD (1) C 13- H 14 0.0743 0.83
25. BD (1) C 1- C 2 39. BD (1) C 13- H 15 -0.1314 4.86
25. BD (1) C 1- C 2 40. BD (1) C 13- C 16 0.0874 1.56
25. BD (1) C 1- C 2 42. BD (1) C 16- H 18 0.0677 0.62
26. BD (1) C 1- N 6 29. BD (1) C 2- O 3 -0.1073 2.01
26. BD (1) C 1- N 6 31. BD (1) C 2- O 4 0.0891 1.71
26. BD (1) C 1- N 6 35. BD (1) C 9- H 10 -0.1034 2.91
26. BD (1) C 1- N 6 37. BD (1) C 9- F 12 0.0531 0.52
26. BD (1) C 1- N 6 38. BD (1) C 13- H 14 -0.1333 4.49
26. BD (1) C 1- N 6 39. BD (1) C 13- H 15 0.0815 1.28
26. BD (1) C 1- N 6 40. BD (1) C 13- C 16 0.0562 0.66
27. BD (1) C 1- C 9 30. BD (2) C 2- O 3 0.1295 3.96
27. BD (1) C 1- C 9 31. BD (1) C 2- O 4 -0.0599 0.90
27. BD (1) C 1- C 9 34. BD (1) N 6- H 8 0.0760 1.32
27. BD (1) C 1- C 9 38. BD (1) C 13- H 14 0.1029 2.14
27. BD (1) C 1- C 9 39. BD (1) C 13- H 15 0.0782 1.35
27. BD (1) C 1- C 9 40. BD (1) C 13- C 16 -0.1508 4.98

28. BD (1) C 1- C 13 29. BD (1) C 2- O 3 0.0565 0.87
28. BD (1) C 1- C 13 30. BD (2) C 2- O 3 -0.1124 2.95
28. BD (1) C 1- C 13 31. BD (1) C 2- O 4 -0.0654 0.92
28. BD (1) C 1- C 13 33. BD (1) N 6- H 7 -0.1271 5.20
28. BD (1) C 1- C 13 34. BD (1) N 6- H 8 0.0718 1.31
28. BD (1) C 1- C 13 35. BD (1) C 9- H 10 0.0745 1.17
28. BD (1) C 1- C 13 36. BD (1) C 9- F 11 0.0545 0.67
28. BD (1) C 1- C 13 37. BD (1) C 9- F 12 -0.1119 3.21
28. BD (1) C 1- C 13 41. BD (1) C 16- H 17 0.0849 1.58
28. BD (1) C 1- C 13 42. BD (1) C 16- H 18 0.0706 1.07
28. BD (1) C 1- C 13 43. BD (1) C 16- C 19 -0.1470 4.32
29. BD (1) C 2- O 3 32. BD (1) O 4- H 5 0.0819 2.08
30. BD (2) C 2- O 3 35. BD (1) C 9- H 10 0.0597 0.61
30. BD (2) C 2- O 3 42. BD (1) C 16- H 18 -0.0832 1.11
34. BD (1) N 6- H 8 39. BD (1) C 13- H 15 0.0757 0.64
35. BD (1) C 9- H 10 38. BD (1) C 13- H 14 0.0794 0.97
38. BD (1) C 13- H 14 41. BD (1) C 16- H 17 -0.1272 5.45
38. BD (1) C 13- H 14 42. BD (1) C 16- H 18 0.0876 1.41
38. BD (1) C 13- H 14 43. BD (1) C 16- C 19 0.0681 1.05
38. BD (1) C 13- H 14 47. BD (1) N 22- H 23 0.0468 0.60
39. BD (1) C 13- H 15 41. BD (1) C 16- H 17 0.0737 0.87
39. BD (1) C 13- H 15 42. BD (1) C 16- H 18 -0.1205 5.14
39. BD (1) C 13- H 15 43. BD (1) C 16- C 19 0.0838 1.64
39. BD (1) C 13- H 15 45. BD (1) C 19- H 21 0.0778 0.79
40. BD (1) C 13- C 16 44. BD (1) C 19- H 20 -0.1331 4.65
40. BD (1) C 13- C 16 45. BD (1) C 19- H 21 0.0805 1.32
40. BD (1) C 13- C 16 46. BD (1) C 19- N 22 0.0521 0.55
41. BD (1) C 16- H 17 44. BD (1) C 19- H 20 0.0910 2.00
41. BD (1) C 16- H 17 45. BD (1) C 19- H 21 0.0764 1.07
41. BD (1) C 16- H 17 46. BD (1) C 19- N 22 -0.1229 3.98
42. BD (1) C 16- H 18 44. BD (1) C 19- H 20 0.0797 1.32
42. BD (1) C 16- H 18 45. BD (1) C 19- H 21 -0.1106 4.64
42. BD (1) C 16- H 18 46. BD (1) C 19- N 22 0.0646 0.76
43. BD (1) C 16- C 19 48. BD (1) N 22- H 24 0.0617 0.94
44. BD (1) C 19- H 20 47. BD (1) N 22- H 23 -0.1096 5.09
44. BD (1) C 19- H 20 48. BD (1) N 22- H 24 0.0710 1.10
45. BD (1) C 19- H 21 47. BD (1) N 22- H 23 0.0877 1.82
45. BD (1) C 19- H 21 48. BD (1) N 22- H 24 -0.1065 5.02
sum within unit 1: 296.33

Total disjoint NLMO steric exchange energy from pairwise sum: 296.33

Conformer **4**

Standard orientation:

Center Atomic Atomic Coordinates (Angstroms)

Number Number Type X Y Z

----

1 6 0 -0.679955 -0.104791 0.236841

2 6 0 -0.736325 1.411651 -0.053642

3 8 0 -0.642237 2.271909 0.780153

4 8 0 -0.859829 1.677801 -1.376513

5 1 0 -0.877119 2.641499 -1.481381

6 7 0 -0.756299 -0.395483 1.655505

7 1 0 -1.674969 -0.155046 2.014509

8 1 0 -0.091647 0.179679 2.160655

9 6 0 -1.871212 -0.764838 -0.473554

10 1 0 -1.899368 -0.551458 -1.541230

11 9 0 -1.856149 -2.117125 -0.303800

12 9 0 -3.047209 -0.303660 0.079439

13 6 0 0.627523 -0.672376 -0.366798

14 1 0 0.608475 -0.525761 -1.451154

15 1 0 0.615490 -1.747118 -0.174753

16 6 0 1.904300 -0.057611 0.209525

17 1 0 1.948382 -0.232047 1.289911

18 1 0 1.909460 1.029193 0.063103

19 6 0 3.165736 -0.651086 -0.435093

20 1 0 3.164670 -1.737448 -0.298564

21 1 0 3.134853 -0.478822 -1.516380

22 7 0 4.440925 -0.138867 0.063291

23 1 0 4.547269 -0.324193 1.055464

24 1 0 4.508481 0.865664 -0.065305

E [DLPNO-CCSD(T)/CBS] = -694.8691073

Pairwise steric exchange energies dE(i,j) (kcal/mol) and associated pre-NLMO overlaps S(i,j) for disjoint (no common atoms) interactions between NLMOs i,j:

Threshold for printing: 0.50 kcal/mol
NLMO (i) NLMO (j) S(i,j) kcal/mol
===
within unit 1
13. LP (1) O 3 25. BD (1) C 1- C 2 -0.0512 0.78
13. LP (1) O 3 31. BD (1) C 2- O 4 -0.0451 0.80
14. LP (2) O 3 15. LP (1) O 4 -0.0164 -0.56
14. LP (2) O 3 17. LP (1) N 6 -0.0648 0.60
14. LP (2) O 3 25. BD (1) C 1- C 2 0.1654 11.60
14. LP (2) O 3 26. BD (1) C 1- N 6 0.1266 1.55
14. LP (2) O 3 31. BD (1) C 2- O 4 -0.2084 13.42
14. LP (2) O 3 32. BD (1) O 4- H 5 -0.1368 2.28
14. LP (2) O 3 34. BD (1) N 6- H 8 0.0896 1.16
15. LP (1) O 4 25. BD (1) C 1- C 2 0.1106 3.41
15. LP (1) O 4 29. BD (1) C 2- O 3 -0.1265 4.48
15. LP (1) O 4 35. BD (1) C 9- H 10 0.0920 1.58
15. LP (1) O 4 38. BD (1) C 13- H 14 0.0703 0.82
16. LP (2) O 4 27. BD (1) C 1- C 9 -0.0696 0.94
16. LP (2) O 4 28. BD (1) C 1- C 13 0.0899 1.48
16. LP (2) O 4 30. BD (2) C 2- O 3 0.1952 13.98
16. LP (2) O 4 38. BD (1) C 13- H 14 0.0569 0.58
17. LP (1) N 6 19. LP (2) F 11 -0.0538 0.58
17. LP (1) N 6 25. BD (1) C 1- C 2 -0.1950 10.87
17. LP (1) N 6 27. BD (1) C 1- C 9 0.0614 0.67
17. LP (1) N 6 28. BD (1) C 1- C 13 0.1132 3.09
17. LP (1) N 6 29. BD (1) C 2- O 3 -0.0810 0.76
17. LP (1) N 6 39. BD (1) C 13- H 15 0.1178 1.16
18. LP (1) F 11 23. LP (3) F 12 -0.0599 -0.63
19. LP (2) F 11 27. BD (1) C 1- C 9 -0.1496 7.80
19. LP (2) F 11 35. BD (1) C 9- H 10 0.1169 7.63
20. LP (3) F 11 21. LP (1) F 12 -0.0604 -0.65
20. LP (3) F 11 23. LP (3) F 12 0.0572 1.73
20. LP (3) F 11 27. BD (1) C 1- C 9 0.0718 1.82
20. LP (3) F 11 28. BD (1) C 1- C 13 0.0800 0.80
20. LP (3) F 11 35. BD (1) C 9- H 10 0.0532 1.39
20. LP (3) F 11 37. BD (1) C 9- F 12 -0.1447 7.17
20. LP (3) F 11 39. BD (1) C 13- H 15 0.0633 0.90
22. LP (2) F 12 26. BD (1) C 1- N 6 -0.0801 0.51
22. LP (2) F 12 27. BD (1) C 1- C 9 -0.1545 8.31
22. LP (2) F 12 33. BD (1) N 6- H 7 -0.0764 1.09
22. LP (2) F 12 35. BD (1) C 9- H 10 0.1103 6.67
23. LP (3) F 12 25. BD (1) C 1- C 2 0.0812 0.96
23. LP (3) F 12 27. BD (1) C 1- C 9 0.0702 1.69
23. LP (3) F 12 35. BD (1) C 9- H 10 0.0622 1.83
23. LP (3) F 12 36. BD (1) C 9- F 11 -0.1410 6.70
24. LP (1) N 22 41. BD (1) C 16- H 17 -0.0933 0.69
24. LP (1) N 22 42. BD (1) C 16- H 18 -0.0916 0.63
24. LP (1) N 22 43. BD (1) C 16- C 19 -0.2235 12.06
24. LP (1) N 22 44. BD (1) C 19- H 20 0.0619 1.47
24. LP (1) N 22 45. BD (1) C 19- H 21 0.0662 1.66
25. BD (1) C 1- C 2 32. BD (1) O 4- H 5 -0.0925 3.79
25. BD (1) C 1- C 2 34. BD (1) N 6- H 8 0.0889 1.63
25. BD (1) C 1- C 2 35. BD (1) C 9- H 10 0.0908 1.34
25. BD (1) C 1- C 2 36. BD (1) C 9- F 11 -0.1139 3.38
25. BD (1) C 1- C 2 38. BD (1) C 13- H 14 0.0870 1.06
25. BD (1) C 1- C 2 39. BD (1) C 13- H 15 -0.1314 4.87
25. BD (1) C 1- C 2 40. BD (1) C 13- C 16 0.0839 1.34
25. BD (1) C 1- C 2 42. BD (1) C 16- H 18 0.0660 0.56
26. BD (1) C 1- N 6 29. BD (1) C 2- O 3 0.0652 1.01
26. BD (1) C 1- N 6 31. BD (1) C 2- O 4 -0.1097 2.54
26. BD (1) C 1- N 6 35. BD (1) C 9- H 10 -0.1127 3.45
26. BD (1) C 1- N 6 37. BD (1) C 9- F 12 0.0575 0.69
26. BD (1) C 1- N 6 38. BD (1) C 13- H 14 -0.1345 4.56
26. BD (1) C 1- N 6 39. BD (1) C 13- H 15 0.0725 1.05
26. BD (1) C 1- N 6 40. BD (1) C 13- C 16 0.0598 0.80
27. BD (1) C 1- C 9 29. BD (1) C 2- O 3 -0.0764 1.07
27. BD (1) C 1- C 9 30. BD (2) C 2- O 3 -0.0995 2.23
27. BD (1) C 1- C 9 31. BD (1) C 2- O 4 0.0679 0.82
27. BD (1) C 1- C 9 33. BD (1) N 6- H 7 0.1018 2.55
27. BD (1) C 1- C 9 34. BD (1) N 6- H 8 -0.1381 6.12
27. BD (1) C 1- C 9 38. BD (1) C 13- H 14 0.0938 1.90
27. BD (1) C 1- C 9 39. BD (1) C 13- H 15 0.0872 1.75

27. BD (1) C 1- C 9 40. BD (1) C 13- C 16 -0.1484 4.72
28. BD (1) C 1- C 13 30. BD (2) C 2- O 3 0.1255 3.50
28. BD (1) C 1- C 13 33. BD (1) N 6- H 7 -0.1306 5.41
28. BD (1) C 1- C 13 34. BD (1) N 6- H 8 0.0646 0.97
28. BD (1) C 1- C 13 35. BD (1) C 9- H 10 0.0658 0.95
28. BD (1) C 1- C 13 36. BD (1) C 9- F 11 0.0512 0.59
28. BD (1) C 1- C 13 37. BD (1) C 9- F 12 -0.1161 3.52
28. BD (1) C 1- C 13 41. BD (1) C 16- H 17 0.0683 1.05
28. BD (1) C 1- C 13 42. BD (1) C 16- H 18 0.0794 1.38
28. BD (1) C 1- C 13 43. BD (1) C 16- C 19 -0.1461 4.31
29. BD (1) C 2- O 3 32. BD (1) O 4- H 5 0.0791 1.88
30. BD (2) C 2- O 3 42. BD (1) C 16- H 18 0.0805 1.13
34. BD (1) N 6- H 8 41. BD (1) C 16- H 17 0.0658 1.05
35. BD (1) C 9- H 10 38. BD (1) C 13- H 14 0.0777 0.84
38. BD (1) C 13- H 14 41. BD (1) C 16- H 17 -0.1238 5.28
38. BD (1) C 13- H 14 42. BD (1) C 16- H 18 0.0722 0.89
38. BD (1) C 13- H 14 43. BD (1) C 16- C 19 0.0851 1.60
38. BD (1) C 13- H 14 45. BD (1) C 19- H 21 0.0739 0.80
39. BD (1) C 13- H 15 41. BD (1) C 16- H 17 0.0805 1.22
39. BD (1) C 13- H 15 42. BD (1) C 16- H 18 -0.1268 5.46
39. BD (1) C 13- H 15 43. BD (1) C 16- C 19 0.0756 1.30
39. BD (1) C 13- H 15 44. BD (1) C 19- H 20 0.0711 0.74
40. BD (1) C 13- C 16 44. BD (1) C 19- H 20 0.0761 1.25
40. BD (1) C 13- C 16 45. BD (1) C 19- H 21 0.0742 1.20
40. BD (1) C 13- C 16 46. BD (1) C 19- N 22 -0.1288 3.42
41. BD (1) C 16- H 17 44. BD (1) C 19- H 20 0.0803 1.16
41. BD (1) C 16- H 17 45. BD (1) C 19- H 21 -0.1142 4.80
41. BD (1) C 16- H 17 46. BD (1) C 19- N 22 0.0667 0.90
42. BD (1) C 16- H 18 44. BD (1) C 19- H 20 -0.1143 4.79
42. BD (1) C 16- H 18 45. BD (1) C 19- H 21 0.0829 1.26
42. BD (1) C 16- H 18 46. BD (1) C 19- N 22 0.0645 0.84
43. BD (1) C 16- C 19 47. BD (1) N 22- H 23 0.0527 0.67
43. BD (1) C 16- C 19 48. BD (1) N 22- H 24 0.0556 0.76
44. BD (1) C 19- H 20 47. BD (1) N 22- H 23 0.0786 1.47
44. BD (1) C 19- H 20 48. BD (1) N 22- H 24 -0.1097 5.19
45. BD (1) C 19- H 21 47. BD (1) N 22- H 23 -0.1089 5.13
45. BD (1) C 19- H 21 48. BD (1) N 22- H 24 0.0755 1.32
sum within unit 1: 293.15

Total disjoint NLMO steric exchange energy from pairwise sum: 293.15

Conformer **5**

Standard orientation:

Center Atomic Atomic Coordinates (Angstroms)

Number Number Type X Y Z

----

1 6 0 0.630105 0.055054 -0.508174

2 6 0 0.817909 1.059720 0.652007

3 8 0 1.255545 2.172254 0.515181

4 8 0 0.420504 0.566141 1.842431

5 1 0 0.601805 1.244647 2.510934

6 7 0 0.934441 0.657096 -1.797395

7 1 0 0.236580 1.353180 -2.032711

8 1 0 1.821814 1.148662 -1.749480

9 6 0 1.631080 -1.104583 -0.363064

10 1 0 1.597275 -1.749359 -1.241875

11 9 0 2.907281 -0.605662 -0.249323

12 9 0 1.400374 -1.862490 0.749142

13 6 0 -0.799041 -0.522954 -0.498788

14 1 0 -0.937422 -1.051974 0.444382

15 1 0 -0.861090 -1.258770 -1.307177

16 6 0 -1.903527 0.520716 -0.675248

17 1 0 -1.829118 0.991460 -1.662089

18 1 0 -1.790464 1.320465 0.066855

19 6 0 -3.315139 -0.079150 -0.541714

20 1 0 -4.047741 0.691749 -0.797994

21 1 0 -3.440441 -0.878249 -1.280095

22 7 0 -3.679182 -0.620252 0.766157

23 1 0 -3.579573 0.078111 1.495709

24 1 0 -3.104057 -1.414445 1.022825

E [DLPNO-CCSD(T)/CBS] = -694.869088

Pairwise steric exchange energies dE(i,j) (kcal/mol) and associated pre-NLMO overlaps S(i,j) for disjoint (no common atoms) interactions between NLMOs i,j:

Threshold for printing: 0.50 kcal/mol
NLMO (i) NLMO (j) S(i,j) kcal/mol
===
within unit 1
13. LP (1) O 3 25. BD (1) C 1- C 2 -0.0502 0.75
13. LP (1) O 3 31. BD (1) C 2- O 4 -0.0459 0.83
14. LP (2) O 3 15. LP (1) O 4 0.0161 -0.56
14. LP (2) O 3 17. LP (1) N 6 0.0660 0.68
14. LP (2) O 3 25. BD (1) C 1- C 2 -0.1691 11.90
14. LP (2) O 3 26. BD (1) C 1- N 6 -0.1317 1.72
14. LP (2) O 3 31. BD (1) C 2- O 4 0.2057 13.10
14. LP (2) O 3 32. BD (1) O 4- H 5 0.1368 2.38
14. LP (2) O 3 34. BD (1) N 6- H 8 -0.0892 1.18
15. LP (1) O 4 22. LP (2) F 12 -0.0517 0.59
15. LP (1) O 4 25. BD (1) C 1- C 2 0.1063 3.23
15. LP (1) O 4 29. BD (1) C 2- O 3 -0.1275 4.56
15. LP (1) O 4 38. BD (1) C 13- H 14 0.0917 1.57
16. LP (2) O 4 27. BD (1) C 1- C 9 -0.0836 1.30
16. LP (2) O 4 28. BD (1) C 1- C 13 0.0713 0.91
16. LP (2) O 4 30. BD (2) C 2- O 3 0.1950 14.18
16. LP (2) O 4 42. BD (1) C 16- H 18 0.0633 0.56
17. LP (1) N 6 25. BD (1) C 1- C 2 -0.1875 10.39
17. LP (1) N 6 27. BD (1) C 1- C 9 0.1254 3.24
17. LP (1) N 6 28. BD (1) C 1- C 13 0.0609 0.80
17. LP (1) N 6 29. BD (1) C 2- O 3 -0.0824 0.78
17. LP (1) N 6 35. BD (1) C 9- H 10 0.1250 1.67
17. LP (1) N 6 39. BD (1) C 13- H 15 0.0976 0.91
18. LP (1) F 11 23. LP (3) F 12 -0.0595 -0.66
19. LP (2) F 11 25. BD (1) C 1- C 2 -0.0924 0.97
19. LP (2) F 11 27. BD (1) C 1- C 9 -0.1560 8.49
19. LP (2) F 11 35. BD (1) C 9- H 10 0.1101 6.78
20. LP (3) F 11 21. LP (1) F 12 -0.0613 -0.67
20. LP (3) F 11 23. LP (3) F 12 0.0604 1.84
20. LP (3) F 11 26. BD (1) C 1- N 6 0.0772 0.73
20. LP (3) F 11 27. BD (1) C 1- C 9 0.0713 1.77
20. LP (3) F 11 34. BD (1) N 6- H 8 0.0559 0.64
20. LP (3) F 11 35. BD (1) C 9- H 10 0.0611 1.71
20. LP (3) F 11 37. BD (1) C 9- F 12 -0.1431 6.82
22. LP (2) F 12 27. BD (1) C 1- C 9 -0.1464 7.69
22. LP (2) F 12 35. BD (1) C 9- H 10 0.1167 7.61
23. LP (3) F 12 27. BD (1) C 1- C 9 0.0725 1.86
23. LP (3) F 12 28. BD (1) C 1- C 13 0.0819 0.83
23. LP (3) F 12 35. BD (1) C 9- H 10 0.0565 1.51
23. LP (3) F 12 36. BD (1) C 9- F 11 -0.1431 7.01
23. LP (3) F 12 38. BD (1) C 13- H 14 0.0644 0.94
24. LP (1) N 22 38. BD (1) C 13- H 14 -0.0874 0.77
24. LP (1) N 22 42. BD (1) C 16- H 18 -0.1025 0.63
24. LP (1) N 22 43. BD (1) C 16- C 19 -0.2258 11.94
24. LP (1) N 22 44. BD (1) C 19- H 20 0.0744 2.11
24. LP (1) N 22 45. BD (1) C 19- H 21 0.0498 1.09
25. BD (1) C 1- C 2 32. BD (1) O 4- H 5 -0.0931 3.85
25. BD (1) C 1- C 2 34. BD (1) N 6- H 8 0.0919 1.75
25. BD (1) C 1- C 2 35. BD (1) C 9- H 10 -0.0988 3.21
25. BD (1) C 1- C 2 36. BD (1) C 9- F 11 0.0766 0.98
25. BD (1) C 1- C 2 38. BD (1) C 13- H 14 0.0873 1.14
25. BD (1) C 1- C 2 39. BD (1) C 13- H 15 -0.1282 4.70
25. BD (1) C 1- C 2 40. BD (1) C 13- C 16 0.0763 1.11
25. BD (1) C 1- C 2 42. BD (1) C 16- H 18 0.0729 0.67
26. BD (1) C 1- N 6 29. BD (1) C 2- O 3 0.0700 1.14
26. BD (1) C 1- N 6 31. BD (1) C 2- O 4 -0.1096 2.49
26. BD (1) C 1- N 6 35. BD (1) C 9- H 10 0.0813 1.44
26. BD (1) C 1- N 6 37. BD (1) C 9- F 12 -0.1112 2.81
26. BD (1) C 1- N 6 38. BD (1) C 13- H 14 -0.1413 4.91
26. BD (1) C 1- N 6 39. BD (1) C 13- H 15 0.0703 0.94
26. BD (1) C 1- N 6 40. BD (1) C 13- C 16 0.0633 0.89
27. BD (1) C 1- C 9 30. BD (2) C 2- O 3 -0.1203 3.21
27. BD (1) C 1- C 9 33. BD (1) N 6- H 7 -0.1336 5.88
27. BD (1) C 1- C 9 34. BD (1) N 6- H 8 0.0681 1.03
27. BD (1) C 1- C 9 38. BD (1) C 13- H 14 0.0885 1.74
27. BD (1) C 1- C 9 39. BD (1) C 13- H 15 0.0927 1.84
27. BD (1) C 1- C 9 40. BD (1) C 13- C 16 -0.1526 5.06
28. BD (1) C 1- C 13 29. BD (1) C 2- O 3 -0.0681 0.72

28. BD (1) C 1- C 13 30. BD (2) C 2- O 3 0.1017 2.27
28. BD (1) C 1- C 13 31. BD (1) C 2- O 4 0.0700 1.06
28. BD (1) C 1- C 13 33. BD (1) N 6- H 7 0.1013 2.58
28. BD (1) C 1- C 13 34. BD (1) N 6- H 8 -0.1327 5.57
28. BD (1) C 1- C 13 35. BD (1) C 9- H 10 0.0665 0.98
28. BD (1) C 1- C 13 36. BD (1) C 9- F 11 -0.1127 3.26
28. BD (1) C 1- C 13 37. BD (1) C 9- F 12 0.0543 0.68
28. BD (1) C 1- C 13 41. BD (1) C 16- H 17 0.0607 0.79
28. BD (1) C 1- C 13 42. BD (1) C 16- H 18 0.0897 1.74
28. BD (1) C 1- C 13 43. BD (1) C 16- C 19 -0.1481 4.39
29. BD (1) C 2- O 3 32. BD (1) O 4- H 5 0.0817 2.01
30. BD (2) C 2- O 3 42. BD (1) C 16- H 18 0.0674 0.70
33. BD (1) N 6- H 7 41. BD (1) C 16- H 17 0.0792 1.47
35. BD (1) C 9- H 10 39. BD (1) C 13- H 15 0.0747 0.73
38. BD (1) C 13- H 14 41. BD (1) C 16- H 17 -0.1248 5.33
38. BD (1) C 13- H 14 42. BD (1) C 16- H 18 0.0677 0.68
38. BD (1) C 13- H 14 43. BD (1) C 16- C 19 0.0912 1.96
38. BD (1) C 13- H 14 48. BD (1) N 22- H 24 0.0555 0.81
39. BD (1) C 13- H 15 41. BD (1) C 16- H 17 0.0914 1.61
39. BD (1) C 13- H 15 42. BD (1) C 16- H 18 -0.1210 5.17
39. BD (1) C 13- H 15 43. BD (1) C 16- C 19 0.0636 0.98
39. BD (1) C 13- H 15 45. BD (1) C 19- H 21 0.0716 0.65
40. BD (1) C 13- C 16 44. BD (1) C 19- H 20 -0.1319 4.59
40. BD (1) C 13- C 16 45. BD (1) C 19- H 21 0.0827 1.39
41. BD (1) C 16- H 17 44. BD (1) C 19- H 20 0.0956 2.19
41. BD (1) C 16- H 17 45. BD (1) C 19- H 21 0.0731 0.95
41. BD (1) C 16- H 17 46. BD (1) C 19- N 22 -0.1219 3.91
42. BD (1) C 16- H 18 44. BD (1) C 19- H 20 0.0781 1.22
42. BD (1) C 16- H 18 45. BD (1) C 19- H 21 -0.1095 4.56
42. BD (1) C 16- H 18 46. BD (1) C 19- N 22 0.0674 0.84
43. BD (1) C 16- C 19 47. BD (1) N 22- H 23 0.0596 0.88
44. BD (1) C 19- H 20 47. BD (1) N 22- H 23 0.0733 1.19
44. BD (1) C 19- H 20 48. BD (1) N 22- H 24 -0.1093 5.09
45. BD (1) C 19- H 21 47. BD (1) N 22- H 23 -0.1066 5.03
45. BD (1) C 19- H 21 48. BD (1) N 22- H 24 0.0864 1.76
sum within unit 1: 293.96

Total disjoint NLMO steric exchange energy from pairwise sum: 293.96

Conformer **6**

Standard orientation:

Center Atomic Atomic Coordinates (Angstroms)

Number Number Type X Y Z

----

1 6 0 -0.682080 -0.106261 0.241609

2 6 0 -0.725636 1.408928 -0.061300

3 8 0 -0.665170 2.276508 0.767406

4 8 0 -0.803268 1.664536 -1.390928

5 1 0 -0.821292 2.627431 -1.502465

6 7 0 -0.779660 -0.389194 1.658876

7 1 0 -0.063729 0.118363 2.165752

8 1 0 -1.672999 -0.073066 2.022274

9 6 0 -1.872248 -0.758810 -0.478845

10 1 0 -1.889655 -0.545430 -1.546962

11 9 0 -1.868443 -2.111069 -0.309944

12 9 0 -3.049100 -0.288094 0.063565

13 6 0 0.625364 -0.691344 -0.343075

14 1 0 0.608401 -0.571791 -1.430513

15 1 0 0.609837 -1.760859 -0.123944

16 6 0 1.899689 -0.063452 0.223095

17 1 0 1.954066 -0.203882 1.305815

18 1 0 1.903715 1.018759 0.043208

19 6 0 3.156730 -0.672629 -0.395487

20 1 0 3.174618 -1.746764 -0.186026

21 1 0 3.107553 -0.562919 -1.491170

22 7 0 4.357926 -0.080942 0.199824

23 1 0 5.197272 -0.543998 -0.130829

24 1 0 4.438825 0.900623 -0.046440

E [DLPNO-CCSD(T)/CBS] = -694.868972

Pairwise steric exchange energies dE(i,j) (kcal/mol) and associated pre-NLMO overlaps S(i,j) for disjoint (no common atoms) interactions between NLMOs i,j:

Threshold for printing: 0.50 kcal/mol
NLMO (i) NLMO (j) S(i,j) kcal/mol
==
within unit 1
13. LP (1) O 3 25. BD (1) C 1- C 2 -0.0518 0.79
13. LP (1) O 3 31. BD (1) C 2- O 4 -0.0453 0.80
14. LP (2) O 3 15. LP (1) O 4 0.0164 -0.56
14. LP (2) O 3 17. LP (1) N 6 0.0676 0.63
14. LP (2) O 3 25. BD (1) C 1- C 2 -0.1651 11.57
14. LP (2) O 3 26. BD (1) C 1- N 6 -0.1258 1.54
14. LP (2) O 3 31. BD (1) C 2- O 4 0.2087 13.45
14. LP (2) O 3 32. BD (1) O 4- H 5 0.1372 2.29
14. LP (2) O 3 33. BD (1) N 6- H 7 -0.0808 0.90
15. LP (1) O 4 25. BD (1) C 1- C 2 0.1104 3.39
15. LP (1) O 4 29. BD (1) C 2- O 3 -0.1261 4.45
15. LP (1) O 4 35. BD (1) C 9- H 10 0.0906 1.52
15. LP (1) O 4 38. BD (1) C 13- H 14 0.0728 0.89
16. LP (2) O 4 27. BD (1) C 1- C 9 -0.0717 0.99
16. LP (2) O 4 28. BD (1) C 1- C 13 0.0884 1.42
16. LP (2) O 4 30. BD (2) C 2- O 3 0.1951 13.96
16. LP (2) O 4 35. BD (1) C 9- H 10 -0.0493 0.53
16. LP (2) O 4 38. BD (1) C 13- H 14 0.0547 0.53
17. LP (1) N 6 19. LP (2) F 11 -0.0555 0.60
17. LP (1) N 6 25. BD (1) C 1- C 2 -0.1983 11.14
17. LP (1) N 6 27. BD (1) C 1- C 9 0.0773 1.16
17. LP (1) N 6 28. BD (1) C 1- C 13 0.0995 2.40
17. LP (1) N 6 29. BD (1) C 2- O 3 -0.0820 0.77
17. LP (1) N 6 39. BD (1) C 13- H 15 0.1146 1.17
18. LP (1) F 11 23. LP (3) F 12 -0.0596 -0.63
19. LP (2) F 11 27. BD (1) C 1- C 9 -0.1490 7.74
19. LP (2) F 11 35. BD (1) C 9- H 10 0.1175 7.69
20. LP (3) F 11 21. LP (1) F 12 -0.0603 -0.65
20. LP (3) F 11 23. LP (3) F 12 0.0570 1.72
20. LP (3) F 11 27. BD (1) C 1- C 9 0.0724 1.86
20. LP (3) F 11 28. BD (1) C 1- C 13 0.0807 0.82
20. LP (3) F 11 35. BD (1) C 9- H 10 0.0531 1.38
20. LP (3) F 11 37. BD (1) C 9- F 12 -0.1446 7.15
20. LP (3) F 11 39. BD (1) C 13- H 15 0.0628 0.89
22. LP (2) F 12 26. BD (1) C 1- N 6 -0.0802 0.53
22. LP (2) F 12 27. BD (1) C 1- C 9 -0.1546 8.33
22. LP (2) F 12 34. BD (1) N 6- H 8 -0.0738 1.02
22. LP (2) F 12 35. BD (1) C 9- H 10 0.1105 6.68
23. LP (3) F 12 25. BD (1) C 1- C 2 0.0814 0.96
23. LP (3) F 12 27. BD (1) C 1- C 9 0.0708 1.71
23. LP (3) F 12 35. BD (1) C 9- H 10 0.0625 1.84
23. LP (3) F 12 36. BD (1) C 9- F 11 -0.1408 6.68
24. LP (1) N 22 41. BD (1) C 16- H 17 0.1014 1.15
24. LP (1) N 22 43. BD (1) C 16- C 19 0.0992 2.41
24. LP (1) N 22 44. BD (1) C 19- H 20 0.0704 1.20
24. LP (1) N 22 45. BD (1) C 19- H 21 -0.1836 11.57
25. BD (1) C 1- C 2 32. BD (1) O 4- H 5 -0.0923 3.77
25. BD (1) C 1- C 2 33. BD (1) N 6- H 7 0.0785 1.19
25. BD (1) C 1- C 2 34. BD (1) N 6- H 8 0.0638 0.71
25. BD (1) C 1- C 2 35. BD (1) C 9- H 10 0.0923 1.39
25. BD (1) C 1- C 2 36. BD (1) C 9- F 11 -0.1138 3.38
25. BD (1) C 1- C 2 38. BD (1) C 13- H 14 0.0836 0.95
25. BD (1) C 1- C 2 39. BD (1) C 13- H 15 -0.1321 4.89
25. BD (1) C 1- C 2 40. BD (1) C 13- C 16 0.0862 1.42
25. BD (1) C 1- C 2 42. BD (1) C 16- H 18 0.0688 0.61
26. BD (1) C 1- N 6 29. BD (1) C 2- O 3 0.0650 1.00
26. BD (1) C 1- N 6 31. BD (1) C 2- O 4 -0.1099 2.54
26. BD (1) C 1- N 6 35. BD (1) C 9- H 10 -0.1125 3.44
26. BD (1) C 1- N 6 37. BD (1) C 9- F 12 0.0583 0.70
26. BD (1) C 1- N 6 38. BD (1) C 13- H 14 -0.1338 4.54
26. BD (1) C 1- N 6 39. BD (1) C 13- H 15 0.0756 1.14
26. BD (1) C 1- N 6 40. BD (1) C 13- C 16 0.0572 0.74
27. BD (1) C 1- C 9 29. BD (1) C 2- O 3 -0.0731 0.97
27. BD (1) C 1- C 9 30. BD (2) C 2- O 3 -0.1024 2.36
27. BD (1) C 1- C 9 31. BD (1) C 2- O 4 0.0663 0.78
27. BD (1) C 1- C 9 33. BD (1) N 6- H 7 -0.1385 6.16
27. BD (1) C 1- C 9 34. BD (1) N 6- H 8 0.0930 2.08
27. BD (1) C 1- C 9 38. BD (1) C 13- H 14 0.0971 2.06

27. BD (1) C 1- C 9 39. BD (1) C 13- H 15 0.0838 1.61
27. BD (1) C 1- C 9 40. BD (1) C 13- C 16 -0.1487 4.75
28. BD (1) C 1- C 13 30. BD (2) C 2- O 3 0.1232 3.37
28. BD (1) C 1- C 13 31. BD (1) C 2- O 4 0.0500 0.52
28. BD (1) C 1- C 13 33. BD (1) N 6- H 7 0.0757 1.37
28. BD (1) C 1- C 13 34. BD (1) N 6- H 8 -0.1321 5.55
28. BD (1) C 1- C 13 35. BD (1) C 9- H 10 0.0656 0.94
28. BD (1) C 1- C 13 36. BD (1) C 9- F 11 0.0518 0.61
28. BD (1) C 1- C 13 37. BD (1) C 9- F 12 -0.1157 3.50
28. BD (1) C 1- C 13 41. BD (1) C 16- H 17 0.0705 1.07
28. BD (1) C 1- C 13 42. BD (1) C 16- H 18 0.0809 1.42
28. BD (1) C 1- C 13 43. BD (1) C 16- C 19 -0.1444 4.28
29. BD (1) C 2- O 3 32. BD (1) O 4- H 5 0.0793 1.89
30. BD (2) C 2- O 3 42. BD (1) C 16- H 18 0.0810 1.13
33. BD (1) N 6- H 7 41. BD (1) C 16- H 17 0.0695 1.15
35. BD (1) C 9- H 10 38. BD (1) C 13- H 14 0.0791 0.86
38. BD (1) C 13- H 14 41. BD (1) C 16- H 17 -0.1215 5.21
38. BD (1) C 13- H 14 42. BD (1) C 16- H 18 0.0735 0.91
38. BD (1) C 13- H 14 43. BD (1) C 16- C 19 0.0855 1.60
38. BD (1) C 13- H 14 45. BD (1) C 19- H 21 0.0735 0.82
39. BD (1) C 13- H 15 41. BD (1) C 16- H 17 0.0812 1.21
39. BD (1) C 13- H 15 42. BD (1) C 16- H 18 -0.1259 5.45
39. BD (1) C 13- H 15 43. BD (1) C 16- C 19 0.0763 1.32
39. BD (1) C 13- H 15 44. BD (1) C 19- H 20 0.0692 0.71
40. BD (1) C 13- C 16 44. BD (1) C 19- H 20 0.0687 1.08
40. BD (1) C 13- C 16 45. BD (1) C 19- H 21 0.0747 1.22
40. BD (1) C 13- C 16 46. BD (1) C 19- N 22 -0.1337 3.76
41. BD (1) C 16- H 17 44. BD (1) C 19- H 20 0.0791 1.23
41. BD (1) C 16- H 17 45. BD (1) C 19- H 21 -0.1175 4.94
41. BD (1) C 16- H 17 46. BD (1) C 19- N 22 0.0711 1.07
42. BD (1) C 16- H 18 44. BD (1) C 19- H 20 -0.1195 5.06
42. BD (1) C 16- H 18 45. BD (1) C 19- H 21 0.0778 1.11
42. BD (1) C 16- H 18 46. BD (1) C 19- N 22 0.0732 1.16
43. BD (1) C 16- C 19 47. BD (1) N 22- H 23 -0.1256 4.88
43. BD (1) C 16- C 19 48. BD (1) N 22- H 24 0.0617 1.01
44. BD (1) C 19- H 20 47. BD (1) N 22- H 23 0.0864 2.09
44. BD (1) C 19- H 20 48. BD (1) N 22- H 24 -0.1109 5.24
45. BD (1) C 19- H 21 47. BD (1) N 22- H 23 0.0490 0.52
45. BD (1) C 19- H 21 48. BD (1) N 22- H 24 0.0664 0.96
sum within unit 1: 294.28

Total disjoint NLMO steric exchange energy from pairwise sum: 294.28

Conformer **7**

Standard orientation:

Center Atomic Atomic Coordinates (Angstroms)

Number Number Type X Y Z

----

1 6 0 -0.600847 -0.084642 0.256154

2 6 0 -1.006104 1.372861 -0.061491

3 8 0 -1.211041 2.224048 0.761017

4 8 0 -1.075143 1.606174 -1.395851

5 1 0 -1.328063 2.534219 -1.517135

6 7 0 -0.686312 -0.389034 1.668684

7 1 0 -1.631573 -0.243845 2.007738

8 1 0 -0.085019 0.230101 2.199292

9 6 0 -1.562601 -1.008954 -0.508345

10 1 0 -1.593852 -0.797541 -1.576670

11 9 0 -1.225312 -2.318294 -0.339091

12 9 0 -2.838419 -0.852216 -0.011121

13 6 0 0.834056 -0.329290 -0.267900

14 1 0 0.828248 -0.244980 -1.359457

15 1 0 1.076147 -1.360527 -0.006500

16 6 0 1.885441 0.624056 0.306245

17 1 0 1.958119 0.499254 1.390347

18 1 0 1.597287 1.663093 0.112077

19 6 0 3.273915 0.391217 -0.292165

20 1 0 3.224870 0.554623 -1.381575

21 1 0 3.956903 1.144274 0.109751

22 7 0 3.802792 -0.925373 0.078018

23 1 0 4.784259 -1.003804 -0.163187

24 1 0 3.317209 -1.672698 -0.405996

E [DLPNO-CCSD(T)/CBS] = -694.8686125

Pairwise steric exchange energies dE(i,j) (kcal/mol) and associated pre-NLMO overlaps S(i,j) for disjoint (no common atoms) interactions between NLMOs i,j:

Threshold for printing: 0.50 kcal/mol
NLMO (i) NLMO (j) S(i,j) kcal/mol
===
within unit 1
13. LP (1) O 3 25. BD (1) C 1- C 2 -0.0522 0.80
13. LP (1) O 3 31. BD (1) C 2- O 4 -0.0456 0.81
14. LP (2) O 3 15. LP (1) O 4 0.0165 -0.57
14. LP (2) O 3 17. LP (1) N 6 0.0690 0.64
14. LP (2) O 3 25. BD (1) C 1- C 2 -0.1647 11.54
14. LP (2) O 3 26. BD (1) C 1- N 6 -0.1253 1.55
14. LP (2) O 3 31. BD (1) C 2- O 4 0.2085 13.45
14. LP (2) O 3 32. BD (1) O 4- H 5 0.1371 2.29
14. LP (2) O 3 34. BD (1) N 6- H 8 -0.0741 0.73
15. LP (1) O 4 25. BD (1) C 1- C 2 0.1109 3.40
15. LP (1) O 4 29. BD (1) C 2- O 3 -0.1259 4.45
15. LP (1) O 4 35. BD (1) C 9- H 10 0.0904 1.51
15. LP (1) O 4 38. BD (1) C 13- H 14 0.0731 0.90
16. LP (2) O 4 27. BD (1) C 1- C 9 -0.0724 1.01
16. LP (2) O 4 28. BD (1) C 1- C 13 0.0880 1.41
16. LP (2) O 4 30. BD (2) C 2- O 3 0.1953 13.97
16. LP (2) O 4 35. BD (1) C 9- H 10 -0.0508 0.56
17. LP (1) N 6 19. LP (2) F 11 -0.0568 0.62
17. LP (1) N 6 25. BD (1) C 1- C 2 -0.1992 11.21
17. LP (1) N 6 27. BD (1) C 1- C 9 0.0908 1.68
17. LP (1) N 6 28. BD (1) C 1- C 13 0.0888 1.90
17. LP (1) N 6 29. BD (1) C 2- O 3 -0.0827 0.77
17. LP (1) N 6 39. BD (1) C 13- H 15 0.1115 1.18
18. LP (1) F 11 23. LP (3) F 12 -0.0595 -0.63
19. LP (2) F 11 27. BD (1) C 1- C 9 -0.1486 7.71
19. LP (2) F 11 35. BD (1) C 9- H 10 0.1178 7.72
20. LP (3) F 11 21. LP (1) F 12 -0.0602 -0.65
20. LP (3) F 11 23. LP (3) F 12 0.0567 1.70
20. LP (3) F 11 27. BD (1) C 1- C 9 0.0732 1.90
20. LP (3) F 11 28. BD (1) C 1- C 13 0.0815 0.82
20. LP (3) F 11 35. BD (1) C 9- H 10 0.0527 1.36
20. LP (3) F 11 37. BD (1) C 9- F 12 -0.1445 7.14
20. LP (3) F 11 39. BD (1) C 13- H 15 0.0621 0.86
22. LP (2) F 12 26. BD (1) C 1- N 6 -0.0805 0.55
22. LP (2) F 12 27. BD (1) C 1- C 9 -0.1545 8.32
22. LP (2) F 12 33. BD (1) N 6- H 7 -0.0716 0.96
22. LP (2) F 12 35. BD (1) C 9- H 10 0.1106 6.70
23. LP (3) F 12 25. BD (1) C 1- C 2 0.0813 0.95
23. LP (3) F 12 27. BD (1) C 1- C 9 0.0712 1.73
23. LP (3) F 12 35. BD (1) C 9- H 10 0.0626 1.85
23. LP (3) F 12 36. BD (1) C 9- F 11 -0.1406 6.67
24. LP (1) N 22 41. BD (1) C 16- H 17 0.1150 1.16
24. LP (1) N 22 43. BD (1) C 16- C 19 0.1159 3.18
24. LP (1) N 22 44. BD (1) C 19- H 20 -0.1732 10.85
24. LP (1) N 22 45. BD (1) C 19- H 21 0.0613 0.81
25. BD (1) C 1- C 2 32. BD (1) O 4- H 5 -0.0921 3.76
25. BD (1) C 1- C 2 33. BD (1) N 6- H 7 0.0712 0.97
25. BD (1) C 1- C 2 34. BD (1) N 6- H 8 0.0695 0.86
25. BD (1) C 1- C 2 35. BD (1) C 9- H 10 0.0937 1.43
25. BD (1) C 1- C 2 36. BD (1) C 9- F 11 -0.1135 3.36
25. BD (1) C 1- C 2 38. BD (1) C 13- H 14 0.0797 0.83
25. BD (1) C 1- C 2 39. BD (1) C 13- H 15 -0.1313 4.87
25. BD (1) C 1- C 2 40. BD (1) C 13- C 16 0.0886 1.53
25. BD (1) C 1- C 2 42. BD (1) C 16- H 18 0.0713 0.66
26. BD (1) C 1- N 6 29. BD (1) C 2- O 3 0.0647 0.98
26. BD (1) C 1- N 6 31. BD (1) C 2- O 4 -0.1103 2.56
26. BD (1) C 1- N 6 35. BD (1) C 9- H 10 -0.1122 3.42
26. BD (1) C 1- N 6 37. BD (1) C 9- F 12 0.0592 0.73
26. BD (1) C 1- N 6 38. BD (1) C 13- H 14 -0.1324 4.47
26. BD (1) C 1- N 6 39. BD (1) C 13- H 15 0.0808 1.32
26. BD (1) C 1- N 6 40. BD (1) C 13- C 16 0.0519 0.61
27. BD (1) C 1- C 9 29. BD (1) C 2- O 3 -0.0722 0.95
27. BD (1) C 1- C 9 30. BD (2) C 2- O 3 -0.1033 2.40
27. BD (1) C 1- C 9 31. BD (1) C 2- O 4 0.0655 0.75
27. BD (1) C 1- C 9 33. BD (1) N 6- H 7 0.0843 1.68
27. BD (1) C 1- C 9 34. BD (1) N 6- H 8 -0.1386 6.17
27. BD (1) C 1- C 9 38. BD (1) C 13- H 14 0.1015 2.26
27. BD (1) C 1- C 9 39. BD (1) C 13- H 15 0.0799 1.45

27. BD (1) C 1- C 9 40. BD (1) C 13- C 16 -0.1505 4.83
28. BD (1) C 1- C 13 30. BD (2) C 2- O 3 0.1220 3.30
28. BD (1) C 1- C 13 31. BD (1) C 2- O 4 0.0509 0.55
28. BD (1) C 1- C 13 33. BD (1) N 6- H 7 -0.1327 5.60
28. BD (1) C 1- C 13 34. BD (1) N 6- H 8 0.0852 1.77
28. BD (1) C 1- C 13 35. BD (1) C 9- H 10 0.0649 0.91
28. BD (1) C 1- C 13 36. BD (1) C 9- F 11 0.0530 0.64
28. BD (1) C 1- C 13 37. BD (1) C 9- F 12 -0.1152 3.46
28. BD (1) C 1- C 13 41. BD (1) C 16- H 17 0.0648 0.92
28. BD (1) C 1- C 13 42. BD (1) C 16- H 18 0.0881 1.65
28. BD (1) C 1- C 13 43. BD (1) C 16- C 19 -0.1457 4.27
29. BD (1) C 2- O 3 32. BD (1) O 4- H 5 0.0792 1.88
30. BD (2) C 2- O 3 42. BD (1) C 16- H 18 0.0848 1.23
34. BD (1) N 6- H 8 41. BD (1) C 16- H 17 0.0686 1.12
35. BD (1) C 9- H 10 38. BD (1) C 13- H 14 0.0799 0.89
38. BD (1) C 13- H 14 41. BD (1) C 16- H 17 -0.1202 5.11
38. BD (1) C 13- H 14 42. BD (1) C 16- H 18 0.0720 0.82
38. BD (1) C 13- H 14 43. BD (1) C 16- C 19 0.0890 1.81
38. BD (1) C 13- H 14 44. BD (1) C 19- H 20 0.0797 0.83
39. BD (1) C 13- H 15 41. BD (1) C 16- H 17 0.0866 1.40
39. BD (1) C 13- H 15 42. BD (1) C 16- H 18 -0.1248 5.37
39. BD (1) C 13- H 15 43. BD (1) C 16- C 19 0.0690 1.08
39. BD (1) C 13- H 15 48. BD (1) N 22- H 24 0.0565 0.87
40. BD (1) C 13- C 16 44. BD (1) C 19- H 20 0.0794 1.31
40. BD (1) C 13- C 16 45. BD (1) C 19- H 21 -0.1394 4.97
40. BD (1) C 13- C 16 46. BD (1) C 19- N 22 0.0585 0.73
41. BD (1) C 16- H 17 44. BD (1) C 19- H 20 -0.1125 4.71
41. BD (1) C 16- H 17 45. BD (1) C 19- H 21 0.0765 1.31
41. BD (1) C 16- H 17 46. BD (1) C 19- N 22 0.0681 0.90
42. BD (1) C 16- H 18 44. BD (1) C 19- H 20 0.0781 1.08
42. BD (1) C 16- H 18 45. BD (1) C 19- H 21 0.0842 1.83
42. BD (1) C 16- H 18 46. BD (1) C 19- N 22 -0.1300 4.47
43. BD (1) C 16- C 19 47. BD (1) N 22- H 23 -0.1225 4.57
43. BD (1) C 16- C 19 48. BD (1) N 22- H 24 0.0521 0.67
44. BD (1) C 19- H 20 48. BD (1) N 22- H 24 0.0760 1.29
45. BD (1) C 19- H 21 47. BD (1) N 22- H 23 0.0955 2.64
45. BD (1) C 19- H 21 48. BD (1) N 22- H 24 -0.1123 5.26
sum within unit 1: 294.96

Total disjoint NLMO steric exchange energy from pairwise sum: 294.96

Conformer **8**

Standard orientation:

Center Atomic Atomic Coordinates (Angstroms)

Number Number Type X Y Z

----

1 6 0 -0.592068 -0.081419 0.241771

2 6 0 -1.035057 1.370446 -0.045926

3 8 0 -1.194200 2.216191 0.792661

4 8 0 -1.186260 1.608037 -1.371346

5 1 0 -1.450671 2.535064 -1.474977

6 7 0 -0.623175 -0.395449 1.657331

7 1 0 -0.141527 0.327298 2.180228

8 1 0 -1.580893 -0.402551 1.994131

9 6 0 -1.553383 -1.022218 -0.500024

10 1 0 -1.613562 -0.811400 -1.566906

11 9 0 -1.187049 -2.325594 -0.337861

12 9 0 -2.820382 -0.892541 0.027453

13 6 0 0.831663 -0.286319 -0.330041

14 1 0 0.799896 -0.156573 -1.416664

15 1 0 1.091200 -1.325517 -0.121727

16 6 0 1.887881 0.648265 0.260315

17 1 0 1.929716 0.526123 1.348319

18 1 0 1.618722 1.693933 0.069323

19 6 0 3.292812 0.396029 -0.314553

20 1 0 3.267528 0.523589 -1.402296

21 1 0 3.970061 1.165396 0.067903

22 7 0 3.896261 -0.905689 -0.034044

23 1 0 3.947502 -1.081467 0.964241

24 1 0 3.369892 -1.665246 -0.450674

E [DLPNO-CCSD(T)/CBS] = -694.8685829

Pairwise steric exchange energies dE(i,j) (kcal/mol) and associated pre-NLMO overlaps S(i,j) for disjoint (no common atoms) interactions between NLMOs i,j:

Threshold for printing: 0.50 kcal/mol
NLMO (i) NLMO (j) S(i,j) kcal/mol
===
within unit 1
13. LP (1) O 3 25. BD (1) C 1- C 2 -0.0512 0.78
13. LP (1) O 3 31. BD (1) C 2- O 4 -0.0451 0.80
14. LP (2) O 3 15. LP (1) O 4 -0.0163 -0.56
14. LP (2) O 3 17. LP (1) N 6 -0.0647 0.60
14. LP (2) O 3 25. BD (1) C 1- C 2 0.1655 11.60
14. LP (2) O 3 26. BD (1) C 1- N 6 0.1265 1.55
14. LP (2) O 3 31. BD (1) C 2- O 4 -0.2082 13.41
14. LP (2) O 3 32. BD (1) O 4- H 5 -0.1367 2.27
14. LP (2) O 3 33. BD (1) N 6- H 7 0.0891 1.14
15. LP (1) O 4 25. BD (1) C 1- C 2 0.1106 3.41
15. LP (1) O 4 29. BD (1) C 2- O 3 -0.1264 4.47
15. LP (1) O 4 35. BD (1) C 9- H 10 0.0919 1.57
15. LP (1) O 4 38. BD (1) C 13- H 14 0.0704 0.82
16. LP (2) O 4 27. BD (1) C 1- C 9 -0.0697 0.94
16. LP (2) O 4 28. BD (1) C 1- C 13 0.0902 1.48
16. LP (2) O 4 30. BD (2) C 2- O 3 0.1952 13.98
16. LP (2) O 4 38. BD (1) C 13- H 14 0.0560 0.55
17. LP (1) N 6 19. LP (2) F 11 -0.0539 0.58
17. LP (1) N 6 25. BD (1) C 1- C 2 -0.1949 10.85
17. LP (1) N 6 27. BD (1) C 1- C 9 0.0622 0.69
17. LP (1) N 6 28. BD (1) C 1- C 13 0.1132 3.07
17. LP (1) N 6 29. BD (1) C 2- O 3 -0.0810 0.76
17. LP (1) N 6 39. BD (1) C 13- H 15 0.1197 1.22
18. LP (1) F 11 23. LP (3) F 12 -0.0600 -0.63
19. LP (2) F 11 27. BD (1) C 1- C 9 -0.1495 7.79
19. LP (2) F 11 35. BD (1) C 9- H 10 0.1170 7.63
20. LP (3) F 11 21. LP (1) F 12 -0.0603 -0.65
20. LP (3) F 11 23. LP (3) F 12 0.0573 1.73
20. LP (3) F 11 27. BD (1) C 1- C 9 0.0721 1.84
20. LP (3) F 11 28. BD (1) C 1- C 13 0.0809 0.81
20. LP (3) F 11 35. BD (1) C 9- H 10 0.0531 1.39
20. LP (3) F 11 37. BD (1) C 9- F 12 -0.1446 7.15
20. LP (3) F 11 39. BD (1) C 13- H 15 0.0634 0.91
22. LP (2) F 12 26. BD (1) C 1- N 6 -0.0802 0.52
22. LP (2) F 12 27. BD (1) C 1- C 9 -0.1542 8.30
22. LP (2) F 12 34. BD (1) N 6- H 8 -0.0765 1.10
22. LP (2) F 12 35. BD (1) C 9- H 10 0.1103 6.68
23. LP (3) F 12 25. BD (1) C 1- C 2 0.0811 0.96
23. LP (3) F 12 27. BD (1) C 1- C 9 0.0702 1.70
23. LP (3) F 12 35. BD (1) C 9- H 10 0.0623 1.83
23. LP (3) F 12 36. BD (1) C 9- F 11 -0.1411 6.71
24. LP (1) N 22 39. BD (1) C 13- H 15 -0.0827 0.71
24. LP (1) N 22 41. BD (1) C 16- H 17 -0.0975 0.58
24. LP (1) N 22 43. BD (1) C 16- C 19 -0.2268 12.02
24. LP (1) N 22 44. BD (1) C 19- H 20 0.0510 1.12
24. LP (1) N 22 45. BD (1) C 19- H 21 0.0744 2.09
25. BD (1) C 1- C 2 32. BD (1) O 4- H 5 -0.0925 3.79
25. BD (1) C 1- C 2 33. BD (1) N 6- H 7 0.0886 1.61
25. BD (1) C 1- C 2 35. BD (1) C 9- H 10 0.0913 1.36
25. BD (1) C 1- C 2 36. BD (1) C 9- F 11 -0.1142 3.39
25. BD (1) C 1- C 2 38. BD (1) C 13- H 14 0.0847 0.98
25. BD (1) C 1- C 2 39. BD (1) C 13- H 15 -0.1312 4.86
25. BD (1) C 1- C 2 40. BD (1) C 13- C 16 0.0849 1.39
25. BD (1) C 1- C 2 42. BD (1) C 16- H 18 0.0652 0.54
26. BD (1) C 1- N 6 29. BD (1) C 2- O 3 0.0653 1.01
26. BD (1) C 1- N 6 31. BD (1) C 2- O 4 -0.1098 2.54
26. BD (1) C 1- N 6 35. BD (1) C 9- H 10 -0.1126 3.44
26. BD (1) C 1- N 6 37. BD (1) C 9- F 12 0.0579 0.69
26. BD (1) C 1- N 6 38. BD (1) C 13- H 14 -0.1334 4.53
26. BD (1) C 1- N 6 39. BD (1) C 13- H 15 0.0760 1.15
26. BD (1) C 1- N 6 40. BD (1) C 13- C 16 0.0576 0.74
27. BD (1) C 1- C 9 29. BD (1) C 2- O 3 -0.0759 1.06
27. BD (1) C 1- C 9 30. BD (2) C 2- O 3 -0.1000 2.25
27. BD (1) C 1- C 9 31. BD (1) C 2- O 4 0.0675 0.81
27. BD (1) C 1- C 9 33. BD (1) N 6- H 7 -0.1381 6.12
27. BD (1) C 1- C 9 34. BD (1) N 6- H 8 0.1018 2.54
27. BD (1) C 1- C 9 38. BD (1) C 13- H 14 0.0962 2.00
27. BD (1) C 1- C 9 39. BD (1) C 13- H 15 0.0864 1.71

27. BD (1) C 1- C 9 40. BD (1) C 13- C 16 -0.1501 4.79
28. BD (1) C 1- C 13 30. BD (2) C 2- O 3 0.1252 3.48
28. BD (1) C 1- C 13 33. BD (1) N 6- H 7 0.0653 0.99
28. BD (1) C 1- C 13 34. BD (1) N 6- H 8 -0.1299 5.37
28. BD (1) C 1- C 13 35. BD (1) C 9- H 10 0.0666 0.96
28. BD (1) C 1- C 13 36. BD (1) C 9- F 11 0.0522 0.61
28. BD (1) C 1- C 13 37. BD (1) C 9- F 12 -0.1154 3.48
28. BD (1) C 1- C 13 41. BD (1) C 16- H 17 0.0745 1.21
28. BD (1) C 1- C 13 42. BD (1) C 16- H 18 0.0790 1.32
28. BD (1) C 1- C 13 43. BD (1) C 16- C 19 -0.1491 4.45
29. BD (1) C 2- O 3 32. BD (1) O 4- H 5 0.0790 1.88
30. BD (2) C 2- O 3 42. BD (1) C 16- H 18 0.0804 1.11
33. BD (1) N 6- H 7 41. BD (1) C 16- H 17 0.0680 1.10
35. BD (1) C 9- H 10 38. BD (1) C 13- H 14 0.0788 0.86
38. BD (1) C 13- H 14 41. BD (1) C 16- H 17 -0.1206 5.15
38. BD (1) C 13- H 14 42. BD (1) C 16- H 18 0.0793 1.10
38. BD (1) C 13- H 14 43. BD (1) C 16- C 19 0.0787 1.44
38. BD (1) C 13- H 14 44. BD (1) C 19- H 20 0.0768 0.76
39. BD (1) C 13- H 15 41. BD (1) C 16- H 17 0.0808 1.13
39. BD (1) C 13- H 15 42. BD (1) C 16- H 18 -0.1266 5.48
39. BD (1) C 13- H 15 43. BD (1) C 16- C 19 0.0763 1.37
39. BD (1) C 13- H 15 48. BD (1) N 22- H 24 0.0513 0.70
40. BD (1) C 13- C 16 44. BD (1) C 19- H 20 0.0817 1.38
40. BD (1) C 13- C 16 45. BD (1) C 19- H 21 -0.1325 4.62
40. BD (1) C 13- C 16 46. BD (1) C 19- N 22 0.0503 0.50
41. BD (1) C 16- H 17 44. BD (1) C 19- H 20 -0.1109 4.65
41. BD (1) C 16- H 17 45. BD (1) C 19- H 21 0.0794 1.27
41. BD (1) C 16- H 17 46. BD (1) C 19- N 22 0.0665 0.80
42. BD (1) C 16- H 18 44. BD (1) C 19- H 20 0.0747 1.01
42. BD (1) C 16- H 18 45. BD (1) C 19- H 21 0.0924 2.08
42. BD (1) C 16- H 18 46. BD (1) C 19- N 22 -0.1213 3.90
43. BD (1) C 16- C 19 47. BD (1) N 22- H 23 0.0594 0.87
43. BD (1) C 16- C 19 48. BD (1) N 22- H 24 0.0488 0.51
44. BD (1) C 19- H 20 47. BD (1) N 22- H 23 -0.1065 5.02
44. BD (1) C 19- H 20 48. BD (1) N 22- H 24 0.0863 1.74
45. BD (1) C 19- H 21 47. BD (1) N 22- H 23 0.0734 1.20
45. BD (1) C 19- H 21 48. BD (1) N 22- H 24 -0.1098 5.13
sum within unit 1: 294.02

Total disjoint NLMO steric exchange energy from pairwise sum: 294.02

Conformer **9**

Standard orientation:

Center Atomic Atomic Coordinates (Angstroms)

Number Number Type X Y Z

----

1 6 0 -0.650356 -0.087244 0.298889

2 6 0 -0.692904 1.410583 -0.078969

3 8 0 -0.804015 2.312442 0.707065

4 8 0 -0.546269 1.608926 -1.411977

5 1 0 -0.575972 2.564983 -1.570369

6 7 0 -0.998684 -0.314702 1.687504

7 1 0 -0.452873 0.295892 2.284874

8 1 0 -1.973015 -0.079986 1.849171

9 6 0 -1.658158 -0.824672 -0.595939

10 1 0 -1.481907 -0.659162 -1.657995

11 9 0 -1.635479 -2.166699 -0.359022

12 9 0 -2.933240 -0.386899 -0.307498

13 6 0 0.766019 -0.631521 -0.006143

14 1 0 0.948701 -0.534775 -1.080194

15 1 0 0.749714 -1.695685 0.239148

16 6 0 1.885156 0.059387 0.773543

17 1 0 1.720459 -0.071658 1.848373

18 1 0 1.878165 1.139327 0.581197

19 6 0 3.279332 -0.494645 0.432232

20 1 0 4.010392 -0.040841 1.107693

21 1 0 3.301243 -1.570504 0.634684

22 7 0 3.753761 -0.290430 -0.935980

23 1 0 3.750779 0.692855 -1.186992

24 1 0 3.184965 -0.784406 -1.613967

E [DLPNO-CCSD(T)/CBS] = -694.868559

Pairwise steric exchange energies dE(i,j) (kcal/mol) and associated pre-NLMO overlaps S(i,j) for disjoint (no common atoms) interactions between NLMOs i,j:

Threshold for printing: 0.50 kcal/mol
NLMO (i) NLMO (j) S(i,j) kcal/mol
===
within unit 1
13. LP (1) O 3 25. BD (1) C 1- C 2 -0.0513 0.79
13. LP (1) O 3 31. BD (1) C 2- O 4 -0.0454 0.80
14. LP (2) O 3 15. LP (1) O 4 -0.0165 -0.57
14. LP (2) O 3 17. LP (1) N 6 -0.0656 0.60
14. LP (2) O 3 25. BD (1) C 1- C 2 0.1648 11.55
14. LP (2) O 3 26. BD (1) C 1- N 6 0.1261 1.55
14. LP (2) O 3 31. BD (1) C 2- O 4 -0.2083 13.42
14. LP (2) O 3 32. BD (1) O 4- H 5 -0.1367 2.28
14. LP (2) O 3 33. BD (1) N 6- H 7 0.0871 1.09
15. LP (1) O 4 25. BD (1) C 1- C 2 0.1108 3.41
15. LP (1) O 4 29. BD (1) C 2- O 3 -0.1263 4.47
15. LP (1) O 4 35. BD (1) C 9- H 10 0.0916 1.55
15. LP (1) O 4 38. BD (1) C 13- H 14 0.0724 0.88
16. LP (2) O 4 27. BD (1) C 1- C 9 -0.0704 0.96
16. LP (2) O 4 28. BD (1) C 1- C 13 0.0890 1.45
16. LP (2) O 4 30. BD (2) C 2- O 3 0.1952 13.97
16. LP (2) O 4 38. BD (1) C 13- H 14 0.0582 0.59
17. LP (1) N 6 19. LP (2) F 11 -0.0542 0.59
17. LP (1) N 6 25. BD (1) C 1- C 2 -0.1952 10.90
17. LP (1) N 6 27. BD (1) C 1- C 9 0.0651 0.77
17. LP (1) N 6 28. BD (1) C 1- C 13 0.1109 2.96
17. LP (1) N 6 29. BD (1) C 2- O 3 -0.0812 0.77
17. LP (1) N 6 39. BD (1) C 13- H 15 0.1174 1.16
18. LP (1) F 11 23. LP (3) F 12 -0.0599 -0.63
19. LP (2) F 11 27. BD (1) C 1- C 9 -0.1494 7.78
19. LP (2) F 11 35. BD (1) C 9- H 10 0.1170 7.64
20. LP (3) F 11 21. LP (1) F 12 -0.0603 -0.65
20. LP (3) F 11 23. LP (3) F 12 0.0573 1.73
20. LP (3) F 11 27. BD (1) C 1- C 9 0.0718 1.83
20. LP (3) F 11 28. BD (1) C 1- C 13 0.0802 0.80
20. LP (3) F 11 35. BD (1) C 9- H 10 0.0535 1.40
20. LP (3) F 11 37. BD (1) C 9- F 12 -0.1445 7.15
20. LP (3) F 11 39. BD (1) C 13- H 15 0.0636 0.91
22. LP (2) F 12 26. BD (1) C 1- N 6 -0.0804 0.52
22. LP (2) F 12 27. BD (1) C 1- C 9 -0.1545 8.32
22. LP (2) F 12 34. BD (1) N 6- H 8 -0.0760 1.08
22. LP (2) F 12 35. BD (1) C 9- H 10 0.1103 6.67
23. LP (3) F 12 25. BD (1) C 1- C 2 0.0810 0.96
23. LP (3) F 12 27. BD (1) C 1- C 9 0.0703 1.69
23. LP (3) F 12 35. BD (1) C 9- H 10 0.0623 1.84
23. LP (3) F 12 36. BD (1) C 9- F 11 -0.1409 6.70
24. LP (1) N 22 38. BD (1) C 13- H 14 -0.0864 0.76
24. LP (1) N 22 42. BD (1) C 16- H 18 -0.0996 0.60
24. LP (1) N 22 43. BD (1) C 16- C 19 -0.2265 12.02
24. LP (1) N 22 44. BD (1) C 19- H 20 0.0749 2.16
24. LP (1) N 22 45. BD (1) C 19- H 21 0.0485 1.05
25. BD (1) C 1- C 2 32. BD (1) O 4- H 5 -0.0921 3.77
25. BD (1) C 1- C 2 33. BD (1) N 6- H 7 0.0869 1.53
25. BD (1) C 1- C 2 34. BD (1) N 6- H 8 0.0568 0.51
25. BD (1) C 1- C 2 35. BD (1) C 9- H 10 0.0916 1.37
25. BD (1) C 1- C 2 36. BD (1) C 9- F 11 -0.1138 3.38
25. BD (1) C 1- C 2 38. BD (1) C 13- H 14 0.0879 1.07
25. BD (1) C 1- C 2 39. BD (1) C 13- H 15 -0.1303 4.82
25. BD (1) C 1- C 2 40. BD (1) C 13- C 16 0.0844 1.35
25. BD (1) C 1- C 2 42. BD (1) C 16- H 18 0.0693 0.61
26. BD (1) C 1- N 6 29. BD (1) C 2- O 3 0.0651 1.00
26. BD (1) C 1- N 6 31. BD (1) C 2- O 4 -0.1098 2.54
26. BD (1) C 1- N 6 35. BD (1) C 9- H 10 -0.1125 3.44
26. BD (1) C 1- N 6 37. BD (1) C 9- F 12 0.0578 0.69
26. BD (1) C 1- N 6 38. BD (1) C 13- H 14 -0.1341 4.55
26. BD (1) C 1- N 6 39. BD (1) C 13- H 15 0.0737 1.07
26. BD (1) C 1- N 6 40. BD (1) C 13- C 16 0.0594 0.79
27. BD (1) C 1- C 9 29. BD (1) C 2- O 3 -0.0755 1.04
27. BD (1) C 1- C 9 30. BD (2) C 2- O 3 -0.1005 2.28
27. BD (1) C 1- C 9 31. BD (1) C 2- O 4 0.0671 0.80
27. BD (1) C 1- C 9 33. BD (1) N 6- H 7 -0.1383 6.14
27. BD (1) C 1- C 9 34. BD (1) N 6- H 8 0.1003 2.46
27. BD (1) C 1- C 9 38. BD (1) C 13- H 14 0.0953 1.96

27. BD (1) C 1- C 9 39. BD (1) C 13- H 15 0.0870 1.73
27. BD (1) C 1- C 9 40. BD (1) C 13- C 16 -0.1505 4.81
28. BD (1) C 1- C 13 30. BD (2) C 2- O 3 0.1246 3.45
28. BD (1) C 1- C 13 33. BD (1) N 6- H 7 0.0672 1.05
28. BD (1) C 1- C 13 34. BD (1) N 6- H 8 -0.1305 5.41
28. BD (1) C 1- C 13 35. BD (1) C 9- H 10 0.0666 0.96
28. BD (1) C 1- C 13 36. BD (1) C 9- F 11 0.0522 0.61
28. BD (1) C 1- C 13 37. BD (1) C 9- F 12 -0.1155 3.49
28. BD (1) C 1- C 13 41. BD (1) C 16- H 17 0.0708 1.08
28. BD (1) C 1- C 13 42. BD (1) C 16- H 18 0.0821 1.45
28. BD (1) C 1- C 13 43. BD (1) C 16- C 19 -0.1490 4.44
29. BD (1) C 2- O 3 32. BD (1) O 4- H 5 0.0793 1.89
30. BD (2) C 2- O 3 42. BD (1) C 16- H 18 0.0817 1.15
33. BD (1) N 6- H 7 41. BD (1) C 16- H 17 0.0673 1.07
35. BD (1) C 9- H 10 38. BD (1) C 13- H 14 0.0782 0.85
38. BD (1) C 13- H 14 41. BD (1) C 16- H 17 -0.1248 5.36
38. BD (1) C 13- H 14 42. BD (1) C 16- H 18 0.0749 0.91
38. BD (1) C 13- H 14 43. BD (1) C 16- C 19 0.0826 1.58
38. BD (1) C 13- H 14 48. BD (1) N 22- H 24 0.0520 0.72
39. BD (1) C 13- H 15 41. BD (1) C 16- H 17 0.0848 1.34
39. BD (1) C 13- H 15 42. BD (1) C 16- H 18 -0.1239 5.34
39. BD (1) C 13- H 15 43. BD (1) C 16- C 19 0.0723 1.25
39. BD (1) C 13- H 15 45. BD (1) C 19- H 21 0.0744 0.71
40. BD (1) C 13- C 16 44. BD (1) C 19- H 20 -0.1324 4.61
40. BD (1) C 13- C 16 45. BD (1) C 19- H 21 0.0818 1.36
41. BD (1) C 16- H 17 44. BD (1) C 19- H 20 0.0936 2.12
41. BD (1) C 16- H 17 45. BD (1) C 19- H 21 0.0743 1.00
41. BD (1) C 16- H 17 46. BD (1) C 19- N 22 -0.1227 3.95
42. BD (1) C 16- H 18 44. BD (1) C 19- H 20 0.0789 1.26
42. BD (1) C 16- H 18 45. BD (1) C 19- H 21 -0.1095 4.56
42. BD (1) C 16- H 18 46. BD (1) C 19- N 22 0.0659 0.79
43. BD (1) C 16- C 19 47. BD (1) N 22- H 23 0.0601 0.89
44. BD (1) C 19- H 20 47. BD (1) N 22- H 23 0.0720 1.15
44. BD (1) C 19- H 20 48. BD (1) N 22- H 24 -0.1088 5.05
45. BD (1) C 19- H 21 47. BD (1) N 22- H 23 -0.1063 5.00
45. BD (1) C 19- H 21 48. BD (1) N 22- H 24 0.0874 1.80
sum within unit 1: 293.99

Total disjoint NLMO steric exchange energy from pairwise sum: 293.99

Conformer **10**

Standard orientation:

Center Atomic Atomic Coordinates (Angstroms)

Number Number Type X Y Z

----

1 6 0 -0.685056 -0.091367 0.210232

2 6 0 -0.746746 1.362176 -0.312999

3 8 0 -0.830741 1.655813 -1.479456

4 8 0 -0.666365 2.281224 0.670892

5 1 0 -0.698030 3.153708 0.248721

6 7 0 -0.787954 -0.256581 1.647291

7 1 0 -1.680645 0.082882 1.988101

8 1 0 -0.063095 0.265860 2.122815

9 6 0 -1.871412 -0.809081 -0.455069

10 1 0 -1.905587 -0.647211 -1.531778

11 9 0 -1.837825 -2.148656 -0.212234

12 9 0 -3.048451 -0.331865 0.083078

13 6 0 0.626579 -0.717064 -0.318331

14 1 0 0.613730 -0.672344 -1.411404

15 1 0 0.612867 -1.768330 -0.024347

16 6 0 1.899770 -0.046444 0.201580

17 1 0 1.944695 -0.112997 1.294401

18 1 0 1.903728 1.019997 -0.054185

19 6 0 3.164844 -0.697195 -0.378149

20 1 0 3.165579 -1.764610 -0.134335

21 1 0 3.134969 -0.632586 -1.470868

22 7 0 4.437551 -0.134649 0.068925

23 1 0 4.541239 -0.212945 1.075425

24 1 0 4.508829 0.849414 -0.168727

E [DLPNO-CCSD(T)/CBS] = -694.8683005

Pairwise steric exchange energies dE(i,j) (kcal/mol) and associated pre-NLMO overlaps S(i,j) for disjoint (no common atoms) interactions between NLMOs i,j:

Threshold for printing: 0.50 kcal/mol
PNLMO dE(i,j)
NLMO (i) NLMO (j) S(i,j) kcal/mol
===
within unit 1
13. LP (1) O 3 25. BD (1) C 1- C 2 -0.0434 0.68
13. LP (1) O 3 31. BD (1) C 2- O 4 -0.0422 0.73
14. LP (2) O 3 15. LP (1) O 4 -0.0171 -0.60
14. LP (2) O 3 25. BD (1) C 1- C 2 0.1734 11.91
14. LP (2) O 3 27. BD (1) C 1- C 9 0.1207 0.87
14. LP (2) O 3 31. BD (1) C 2- O 4 -0.2057 13.27
14. LP (2) O 3 32. BD (1) O 4- H 5 -0.1372 2.32
14. LP (2) O 3 35. BD (1) C 9- H 10 0.1015 1.55
14. LP (2) O 3 38. BD (1) C 13- H 14 0.0807 0.79
15. LP (1) O 4 17. LP (1) N 6 -0.0760 1.34
15. LP (1) O 4 25. BD (1) C 1- C 2 0.0947 2.96
15. LP (1) O 4 29. BD (1) C 2- O 3 -0.1296 4.59
15. LP (1) O 4 34. BD (1) N 6- H 8 0.0719 0.92
16. LP (2) O 4 27. BD (1) C 1- C 9 -0.0611 0.64
16. LP (2) O 4 28. BD (1) C 1- C 13 0.0782 1.02
16. LP (2) O 4 30. BD (2) C 2- O 3 0.1967 14.33
16. LP (2) O 4 42. BD (1) C 16- H 18 0.0562 0.53
17. LP (1) N 6 19. LP (2) F 11 -0.0535 0.55
17. LP (1) N 6 25. BD (1) C 1- C 2 -0.2089 11.52
17. LP (1) N 6 27. BD (1) C 1- C 9 0.0785 1.32
17. LP (1) N 6 28. BD (1) C 1- C 13 0.0949 2.28
17. LP (1) N 6 39. BD (1) C 13- H 15 0.1123 1.15
18. LP (1) F 11 23. LP (3) F 12 -0.0596 -0.62
19. LP (2) F 11 27. BD (1) C 1- C 9 -0.1496 7.83
19. LP (2) F 11 35. BD (1) C 9- H 10 0.1167 7.63
20. LP (3) F 11 21. LP (1) F 12 -0.0604 -0.65
20. LP (3) F 11 23. LP (3) F 12 0.0573 1.73
20. LP (3) F 11 27. BD (1) C 1- C 9 0.0728 1.88
20. LP (3) F 11 28. BD (1) C 1- C 13 0.0814 0.85
20. LP (3) F 11 35. BD (1) C 9- H 10 0.0529 1.38
20. LP (3) F 11 37. BD (1) C 9- F 12 -0.1449 7.18
20. LP (3) F 11 39. BD (1) C 13- H 15 0.0633 0.92
22. LP (2) F 12 26. BD (1) C 1- N 6 -0.0828 0.60
22. LP (2) F 12 27. BD (1) C 1- C 9 -0.1538 8.25
22. LP (2) F 12 33. BD (1) N 6- H 7 -0.0770 1.14
22. LP (2) F 12 35. BD (1) C 9- H 10 0.1100 6.66
23. LP (3) F 12 25. BD (1) C 1- C 2 0.0806 0.95
23. LP (3) F 12 27. BD (1) C 1- C 9 0.0719 1.76
23. LP (3) F 12 35. BD (1) C 9- H 10 0.0622 1.80
23. LP (3) F 12 36. BD (1) C 9- F 11 -0.1400 6.63
24. LP (1) N 22 41. BD (1) C 16- H 17 -0.0931 0.68
24. LP (1) N 22 42. BD (1) C 16- H 18 -0.0920 0.64
24. LP (1) N 22 43. BD (1) C 16- C 19 -0.2239 12.08
24. LP (1) N 22 44. BD (1) C 19- H 20 0.0633 1.54
24. LP (1) N 22 45. BD (1) C 19- H 21 0.0646 1.60
25. BD (1) C 1- C 2 32. BD (1) O 4- H 5 -0.1010 4.30
25. BD (1) C 1- C 2 33. BD (1) N 6- H 7 0.0617 0.63
25. BD (1) C 1- C 2 34. BD (1) N 6- H 8 0.0710 0.88
25. BD (1) C 1- C 2 35. BD (1) C 9- H 10 0.0992 1.75
25. BD (1) C 1- C 2 36. BD (1) C 9- F 11 -0.1111 3.29
25. BD (1) C 1- C 2 38. BD (1) C 13- H 14 0.0879 1.16
25. BD (1) C 1- C 2 39. BD (1) C 13- H 15 -0.1304 4.85
25. BD (1) C 1- C 2 40. BD (1) C 13- C 16 0.0825 1.33
25. BD (1) C 1- C 2 42. BD (1) C 16- H 18 0.0664 0.57
26. BD (1) C 1- N 6 29. BD (1) C 2- O 3 -0.1010 1.76
26. BD (1) C 1- N 6 31. BD (1) C 2- O 4 0.0819 1.38
26. BD (1) C 1- N 6 35. BD (1) C 9- H 10 -0.1108 3.33
26. BD (1) C 1- N 6 37. BD (1) C 9- F 12 0.0629 0.83
26. BD (1) C 1- N 6 38. BD (1) C 13- H 14 -0.1338 4.51
26. BD (1) C 1- N 6 39. BD (1) C 13- H 15 0.0731 1.03
26. BD (1) C 1- N 6 40. BD (1) C 13- C 16 0.0601 0.81
27. BD (1) C 1- C 9 29. BD (1) C 2- O 3 0.0576 0.74
27. BD (1) C 1- C 9 30. BD (2) C 2- O 3 -0.1008 2.42
27. BD (1) C 1- C 9 31. BD (1) C 2- O 4 -0.0783 1.51
27. BD (1) C 1- C 9 33. BD (1) N 6- H 7 0.0894 1.95
27. BD (1) C 1- C 9 34. BD (1) N 6- H 8 -0.1387 6.16
27. BD (1) C 1- C 9 38. BD (1) C 13- H 14 0.0959 1.98

27. BD (1) C 1- C 9 39. BD (1) C 13- H 15 0.0857 1.71
27. BD (1) C 1- C 9 40. BD (1) C 13- C 16 -0.1490 4.76
28. BD (1) C 1- C 13 30. BD (2) C 2- O 3 0.1344 4.16
28. BD (1) C 1- C 13 33. BD (1) N 6- H 7 -0.1314 5.50
28. BD (1) C 1- C 13 34. BD (1) N 6- H 8 0.0775 1.45
28. BD (1) C 1- C 13 35. BD (1) C 9- H 10 0.0609 0.78
28. BD (1) C 1- C 13 36. BD (1) C 9- F 11 0.0559 0.71
28. BD (1) C 1- C 13 37. BD (1) C 9- F 12 -0.1159 3.49
28. BD (1) C 1- C 13 41. BD (1) C 16- H 17 0.0675 1.02
28. BD (1) C 1- C 13 42. BD (1) C 16- H 18 0.0797 1.39
28. BD (1) C 1- C 13 43. BD (1) C 16- C 19 -0.1459 4.30
29. BD (1) C 2- O 3 32. BD (1) O 4- H 5 0.0813 2.05
30. BD (2) C 2- O 3 38. BD (1) C 13- H 14 0.0642 0.62
30. BD (2) C 2- O 3 42. BD (1) C 16- H 18 0.0726 0.75
34. BD (1) N 6- H 8 41. BD (1) C 16- H 17 0.0703 1.19
35. BD (1) C 9- H 10 38. BD (1) C 13- H 14 0.0756 0.77
38. BD (1) C 13- H 14 41. BD (1) C 16- H 17 -0.1239 5.29
38. BD (1) C 13- H 14 42. BD (1) C 16- H 18 0.0723 0.90
38. BD (1) C 13- H 14 43. BD (1) C 16- C 19 0.0857 1.63
38. BD (1) C 13- H 14 45. BD (1) C 19- H 21 0.0744 0.81
39. BD (1) C 13- H 15 41. BD (1) C 16- H 17 0.0804 1.20
39. BD (1) C 13- H 15 42. BD (1) C 16- H 18 -0.1265 5.45
39. BD (1) C 13- H 15 43. BD (1) C 16- C 19 0.0752 1.29
39. BD (1) C 13- H 15 44. BD (1) C 19- H 20 0.0712 0.74
40. BD (1) C 13- C 16 44. BD (1) C 19- H 20 0.0762 1.25
40. BD (1) C 13- C 16 45. BD (1) C 19- H 21 0.0745 1.21
40. BD (1) C 13- C 16 46. BD (1) C 19- N 22 -0.1288 3.43
41. BD (1) C 16- H 17 44. BD (1) C 19- H 20 0.0807 1.17
41. BD (1) C 16- H 17 45. BD (1) C 19- H 21 -0.1142 4.80
41. BD (1) C 16- H 17 46. BD (1) C 19- N 22 0.0666 0.90
42. BD (1) C 16- H 18 44. BD (1) C 19- H 20 -0.1146 4.80
42. BD (1) C 16- H 18 45. BD (1) C 19- H 21 0.0825 1.25
42. BD (1) C 16- H 18 46. BD (1) C 19- N 22 0.0642 0.84
43. BD (1) C 16- C 19 47. BD (1) N 22- H 23 0.0534 0.69
43. BD (1) C 16- C 19 48. BD (1) N 22- H 24 0.0543 0.72
44. BD (1) C 19- H 20 47. BD (1) N 22- H 23 0.0776 1.42
44. BD (1) C 19- H 20 48. BD (1) N 22- H 24 -0.1093 5.16
45. BD (1) C 19- H 21 47. BD (1) N 22- H 23 -0.1088 5.13
45. BD (1) C 19- H 21 48. BD (1) N 22- H 24 0.0767 1.37
sum within unit 1: 293.84

Total disjoint NLMO steric exchange energy from pairwise sum: 293.84

Conformer **11**

Standard orientation:

Center Atomic Atomic Coordinates (Angstroms)

Number Number Type X Y Z

----

1 6 0 -0.596239 -0.074929 0.208436

2 6 0 -1.043148 1.320003 -0.285649

3 8 0 -1.205548 1.603410 -1.446327

4 8 0 -1.206424 2.207404 0.716584

5 1 0 -1.467011 3.049446 0.312163

6 7 0 -0.656726 -0.294348 1.640918

7 1 0 -1.609440 -0.215092 1.979187

8 1 0 -0.101992 0.393764 2.134138

9 6 0 -1.543495 -1.069723 -0.483087

10 1 0 -1.617427 -0.897521 -1.556156

11 9 0 -1.149575 -2.356450 -0.270152

12 9 0 -2.806633 -0.941472 0.055220

13 6 0 0.837527 -0.315884 -0.318713

14 1 0 0.818817 -0.260375 -1.411675

15 1 0 1.098200 -1.336535 -0.034682

16 6 0 1.880802 0.665494 0.216894

17 1 0 1.917862 0.619461 1.311408

18 1 0 1.605078 1.692672 -0.050244

19 6 0 3.292788 0.388240 -0.329299

20 1 0 3.274625 0.447365 -1.422744

21 1 0 3.961437 1.185229 0.009377

22 7 0 3.902348 -0.888797 0.037002

23 1 0 3.388028 -1.676811 -0.339793

24 1 0 3.944697 -1.004016 1.044434

E [DLPNO-CCSD(T)/CBS] = -694.8679437

Pairwise steric exchange energies dE(i,j) (kcal/mol) and associated pre-NLMO overlaps S(i,j) for disjoint (no common atoms) interactions between NLMOs i,j:

Threshold for printing: 0.50 kcal/mol
NLMO (i) NLMO (j) S(i,j) kcal/mol
===
within unit 1
13. LP (1) O 3 25. BD (1) C 1- C 2 -0.0434 0.68
13. LP (1) O 3 31. BD (1) C 2- O 4 -0.0422 0.73
14. LP (2) O 3 15. LP (1) O 4 -0.0171 -0.59
14. LP (2) O 3 25. BD (1) C 1- C 2 0.1736 11.93
14. LP (2) O 3 27. BD (1) C 1- C 9 0.1213 0.89
14. LP (2) O 3 31. BD (1) C 2- O 4 -0.2055 13.24
14. LP (2) O 3 32. BD (1) O 4- H 5 -0.1370 2.31
14. LP (2) O 3 35. BD (1) C 9- H 10 0.1023 1.59
14. LP (2) O 3 38. BD (1) C 13- H 14 0.0800 0.76
15. LP (1) O 4 17. LP (1) N 6 -0.0757 1.33
15. LP (1) O 4 25. BD (1) C 1- C 2 0.0950 2.96
15. LP (1) O 4 29. BD (1) C 2- O 3 -0.1296 4.59
15. LP (1) O 4 34. BD (1) N 6- H 8 0.0723 0.93
16. LP (2) O 4 27. BD (1) C 1- C 9 -0.0606 0.63
16. LP (2) O 4 28. BD (1) C 1- C 13 0.0786 1.03
16. LP (2) O 4 30. BD (2) C 2- O 3 0.1967 14.32
16. LP (2) O 4 42. BD (1) C 16- H 18 0.0568 0.53
17. LP (1) N 6 19. LP (2) F 11 -0.0536 0.55
17. LP (1) N 6 25. BD (1) C 1- C 2 -0.2085 11.47
17. LP (1) N 6 27. BD (1) C 1- C 9 0.0787 1.32
17. LP (1) N 6 28. BD (1) C 1- C 13 0.0957 2.32
17. LP (1) N 6 39. BD (1) C 13- H 15 0.1137 1.19
18. LP (1) F 11 23. LP (3) F 12 -0.0597 -0.62
19. LP (2) F 11 27. BD (1) C 1- C 9 -0.1495 7.81
19. LP (2) F 11 35. BD (1) C 9- H 10 0.1168 7.63
20. LP (3) F 11 21. LP (1) F 12 -0.0603 -0.65
20. LP (3) F 11 23. LP (3) F 12 0.0573 1.73
20. LP (3) F 11 27. BD (1) C 1- C 9 0.0730 1.89
20. LP (3) F 11 28. BD (1) C 1- C 13 0.0821 0.85
20. LP (3) F 11 35. BD (1) C 9- H 10 0.0529 1.38
20. LP (3) F 11 37. BD (1) C 9- F 12 -0.1448 7.16
20. LP (3) F 11 39. BD (1) C 13- H 15 0.0638 0.93
22. LP (2) F 12 26. BD (1) C 1- N 6 -0.0828 0.61
22. LP (2) F 12 27. BD (1) C 1- C 9 -0.1535 8.24
22. LP (2) F 12 33. BD (1) N 6- H 7 -0.0772 1.15
22. LP (2) F 12 35. BD (1) C 9- H 10 0.1101 6.67
23. LP (3) F 12 25. BD (1) C 1- C 2 0.0804 0.94
23. LP (3) F 12 27. BD (1) C 1- C 9 0.0718 1.76
23. LP (3) F 12 35. BD (1) C 9- H 10 0.0621 1.79
23. LP (3) F 12 36. BD (1) C 9- F 11 -0.1402 6.65
24. LP (1) N 22 39. BD (1) C 13- H 15 -0.0831 0.72
24. LP (1) N 22 41. BD (1) C 16- H 17 -0.0971 0.56
24. LP (1) N 22 43. BD (1) C 16- C 19 -0.2269 12.03
24. LP (1) N 22 44. BD (1) C 19- H 20 0.0501 1.10
24. LP (1) N 22 45. BD (1) C 19- H 21 0.0750 2.13
25. BD (1) C 1- C 2 32. BD (1) O 4- H 5 -0.1010 4.31
25. BD (1) C 1- C 2 33. BD (1) N 6- H 7 0.0617 0.63
25. BD (1) C 1- C 2 34. BD (1) N 6- H 8 0.0711 0.89
25. BD (1) C 1- C 2 35. BD (1) C 9- H 10 0.0993 1.76
25. BD (1) C 1- C 2 36. BD (1) C 9- F 11 -0.1113 3.29
25. BD (1) C 1- C 2 38. BD (1) C 13- H 14 0.0868 1.12
25. BD (1) C 1- C 2 39. BD (1) C 13- H 15 -0.1302 4.85
25. BD (1) C 1- C 2 40. BD (1) C 13- C 16 0.0826 1.35
25. BD (1) C 1- C 2 42. BD (1) C 16- H 18 0.0656 0.54
26. BD (1) C 1- N 6 29. BD (1) C 2- O 3 -0.1010 1.76
26. BD (1) C 1- N 6 31. BD (1) C 2- O 4 0.0818 1.36
26. BD (1) C 1- N 6 35. BD (1) C 9- H 10 -0.1107 3.32
26. BD (1) C 1- N 6 37. BD (1) C 9- F 12 0.0633 0.84
26. BD (1) C 1- N 6 38. BD (1) C 13- H 14 -0.1328 4.49
26. BD (1) C 1- N 6 39. BD (1) C 13- H 15 0.0756 1.10
26. BD (1) C 1- N 6 40. BD (1) C 13- C 16 0.0589 0.78
27. BD (1) C 1- C 9 29. BD (1) C 2- O 3 0.0585 0.77
27. BD (1) C 1- C 9 30. BD (2) C 2- O 3 -0.0999 2.38
27. BD (1) C 1- C 9 31. BD (1) C 2- O 4 -0.0790 1.53
27. BD (1) C 1- C 9 33. BD (1) N 6- H 7 0.0898 1.96
27. BD (1) C 1- C 9 34. BD (1) N 6- H 8 -0.1387 6.16
27. BD (1) C 1- C 9 38. BD (1) C 13- H 14 0.0971 2.03
27. BD (1) C 1- C 9 39. BD (1) C 13- H 15 0.0859 1.72

27. BD (1) C 1- C 9 40. BD (1) C 13- C 16 -0.1508 4.83
28. BD (1) C 1- C 13 30. BD (2) C 2- O 3 0.1350 4.19
28. BD (1) C 1- C 13 33. BD (1) N 6- H 7 -0.1306 5.46
28. BD (1) C 1- C 13 34. BD (1) N 6- H 8 0.0777 1.46
28. BD (1) C 1- C 13 35. BD (1) C 9- H 10 0.0616 0.78
28. BD (1) C 1- C 13 36. BD (1) C 9- F 11 0.0568 0.73
28. BD (1) C 1- C 13 37. BD (1) C 9- F 12 -0.1152 3.46
28. BD (1) C 1- C 13 41. BD (1) C 16- H 17 0.0728 1.15
28. BD (1) C 1- C 13 42. BD (1) C 16- H 18 0.0800 1.35
28. BD (1) C 1- C 13 43. BD (1) C 16- C 19 -0.1489 4.44
29. BD (1) C 2- O 3 32. BD (1) O 4- H 5 0.0812 2.04
30. BD (2) C 2- O 3 38. BD (1) C 13- H 14 0.0644 0.61
30. BD (2) C 2- O 3 42. BD (1) C 16- H 18 0.0730 0.76
34. BD (1) N 6- H 8 41. BD (1) C 16- H 17 0.0726 1.25
35. BD (1) C 9- H 10 38. BD (1) C 13- H 14 0.0761 0.79
38. BD (1) C 13- H 14 41. BD (1) C 16- H 17 -0.1207 5.14
38. BD (1) C 13- H 14 42. BD (1) C 16- H 18 0.0786 1.08
38. BD (1) C 13- H 14 43. BD (1) C 16- C 19 0.0801 1.50
38. BD (1) C 13- H 14 44. BD (1) C 19- H 20 0.0773 0.77
39. BD (1) C 13- H 15 41. BD (1) C 16- H 17 0.0815 1.15
39. BD (1) C 13- H 15 42. BD (1) C 16- H 18 -0.1264 5.47
39. BD (1) C 13- H 15 43. BD (1) C 16- C 19 0.0751 1.33
39. BD (1) C 13- H 15 47. BD (1) N 22- H 23 0.0507 0.69
40. BD (1) C 13- C 16 44. BD (1) C 19- H 20 0.0811 1.36
40. BD (1) C 13- C 16 45. BD (1) C 19- H 21 -0.1325 4.62
40. BD (1) C 13- C 16 46. BD (1) C 19- N 22 0.0508 0.52
41. BD (1) C 16- H 17 44. BD (1) C 19- H 20 -0.1110 4.64
41. BD (1) C 16- H 17 45. BD (1) C 19- H 21 0.0803 1.30
41. BD (1) C 16- H 17 46. BD (1) C 19- N 22 0.0659 0.78
42. BD (1) C 16- H 18 44. BD (1) C 19- H 20 0.0754 1.04
42. BD (1) C 16- H 18 45. BD (1) C 19- H 21 0.0915 2.04
42. BD (1) C 16- H 18 46. BD (1) C 19- N 22 -0.1216 3.91
43. BD (1) C 16- C 19 48. BD (1) N 22- H 24 0.0597 0.88
44. BD (1) C 19- H 20 47. BD (1) N 22- H 23 0.0868 1.77
44. BD (1) C 19- H 20 48. BD (1) N 22- H 24 -0.1062 5.01
45. BD (1) C 19- H 21 47. BD (1) N 22- H 23 -0.1097 5.12
45. BD (1) C 19- H 21 48. BD (1) N 22- H 24 0.0729 1.18
sum within unit 1: 294.75

Total disjoint NLMO steric exchange energy from pairwise sum: 294.75

Conformer **12**

Standard orientation:

Center Atomic Atomic Coordinates (Angstroms)

Number Number Type X Y Z

----

1 6 0 -0.647270 -0.072578 0.272310

2 6 0 -0.678338 1.343438 -0.348483

3 8 0 -0.529550 1.566434 -1.524335

4 8 0 -0.861727 2.317368 0.565590

5 1 0 -0.854330 3.161526 0.088257

6 7 0 -1.021017 -0.165765 1.669850

7 1 0 -0.423435 0.416195 2.243150

8 1 0 -1.975890 0.144280 1.812159

9 6 0 -1.643166 -0.897056 -0.560760

10 1 0 -1.474340 -0.794984 -1.632221

11 9 0 -1.589561 -2.217335 -0.231202

12 9 0 -2.925176 -0.466201 -0.290772

13 6 0 0.772055 -0.642524 0.045985

14 1 0 0.962639 -0.651531 -1.030653

15 1 0 0.757549 -1.676238 0.397718

16 6 0 1.881670 0.132447 0.757587

17 1 0 1.721251 0.104606 1.841145

18 1 0 1.862887 1.187981 0.459112

19 6 0 3.283606 -0.433920 0.469287

20 1 0 4.008546 0.093388 1.096525

21 1 0 3.319489 -1.484419 0.776103

22 7 0 3.753266 -0.357622 -0.912769

23 1 0 3.726154 0.594788 -1.262270

24 1 0 3.195244 -0.929304 -1.536420

E [DLPNO-CCSD(T)/CBS] = -694.8679282

Pairwise steric exchange energies dE(i,j) (kcal/mol) and associated pre-NLMO overlaps S(i,j) for disjoint (no common atoms) interactions between NLMOs i,j:

Threshold for printing: 0.50 kcal/mol
NLMO (i) NLMO (j) S(i,j) kcal/mol
===
within unit 1
13. LP (1) O 3 25. BD (1) C 1- C 2 -0.0432 0.68
13. LP (1) O 3 31. BD (1) C 2- O 4 -0.0420 0.73
14. LP (2) O 3 15. LP (1) O 4 -0.0172 -0.60
14. LP (2) O 3 25. BD (1) C 1- C 2 0.1734 11.91
14. LP (2) O 3 27. BD (1) C 1- C 9 0.1195 0.83
14. LP (2) O 3 31. BD (1) C 2- O 4 -0.2056 13.25
14. LP (2) O 3 32. BD (1) O 4- H 5 -0.1371 2.32
14. LP (2) O 3 35. BD (1) C 9- H 10 0.1004 1.51
14. LP (2) O 3 38. BD (1) C 13- H 14 0.0842 0.87
15. LP (1) O 4 17. LP (1) N 6 -0.0763 1.35
15. LP (1) O 4 25. BD (1) C 1- C 2 0.0946 2.96
15. LP (1) O 4 29. BD (1) C 2- O 3 -0.1300 4.61
15. LP (1) O 4 33. BD (1) N 6- H 7 0.0698 0.86
16. LP (2) O 4 27. BD (1) C 1- C 9 -0.0622 0.66
16. LP (2) O 4 28. BD (1) C 1- C 13 0.0771 1.00
16. LP (2) O 4 30. BD (2) C 2- O 3 0.1970 14.36
16. LP (2) O 4 42. BD (1) C 16- H 18 0.0572 0.54
17. LP (1) N 6 19. LP (2) F 11 -0.0537 0.55
17. LP (1) N 6 25. BD (1) C 1- C 2 -0.2090 11.53
17. LP (1) N 6 27. BD (1) C 1- C 9 0.0810 1.41
17. LP (1) N 6 28. BD (1) C 1- C 13 0.0933 2.22
17. LP (1) N 6 39. BD (1) C 13- H 15 0.1117 1.14
18. LP (1) F 11 23. LP (3) F 12 -0.0595 -0.62
19. LP (2) F 11 27. BD (1) C 1- C 9 -0.1494 7.82
19. LP (2) F 11 35. BD (1) C 9- H 10 0.1168 7.63
20. LP (3) F 11 21. LP (1) F 12 -0.0603 -0.65
20. LP (3) F 11 23. LP (3) F 12 0.0572 1.73
20. LP (3) F 11 27. BD (1) C 1- C 9 0.0727 1.88
20. LP (3) F 11 28. BD (1) C 1- C 13 0.0816 0.85
20. LP (3) F 11 35. BD (1) C 9- H 10 0.0531 1.39
20. LP (3) F 11 37. BD (1) C 9- F 12 -0.1447 7.17
20. LP (3) F 11 39. BD (1) C 13- H 15 0.0638 0.93
22. LP (2) F 12 26. BD (1) C 1- N 6 -0.0829 0.61
22. LP (2) F 12 27. BD (1) C 1- C 9 -0.1539 8.27
22. LP (2) F 12 34. BD (1) N 6- H 8 -0.0766 1.12
22. LP (2) F 12 35. BD (1) C 9- H 10 0.1100 6.65
23. LP (3) F 12 25. BD (1) C 1- C 2 0.0803 0.94
23. LP (3) F 12 27. BD (1) C 1- C 9 0.0718 1.75
23. LP (3) F 12 35. BD (1) C 9- H 10 0.0624 1.81
23. LP (3) F 12 36. BD (1) C 9- F 11 -0.1400 6.63
24. LP (1) N 22 38. BD (1) C 13- H 14 -0.0878 0.78
24. LP (1) N 22 42. BD (1) C 16- H 18 -0.1001 0.59
24. LP (1) N 22 43. BD (1) C 16- C 19 -0.2266 12.00
24. LP (1) N 22 44. BD (1) C 19- H 20 0.0764 2.23
24. LP (1) N 22 45. BD (1) C 19- H 21 0.0469 0.99
25. BD (1) C 1- C 2 32. BD (1) O 4- H 5 -0.1010 4.31
25. BD (1) C 1- C 2 33. BD (1) N 6- H 7 0.0695 0.83
25. BD (1) C 1- C 2 34. BD (1) N 6- H 8 0.0630 0.66
25. BD (1) C 1- C 2 35. BD (1) C 9- H 10 0.0997 1.77
25. BD (1) C 1- C 2 36. BD (1) C 9- F 11 -0.1112 3.29
25. BD (1) C 1- C 2 38. BD (1) C 13- H 14 0.0897 1.21
25. BD (1) C 1- C 2 39. BD (1) C 13- H 15 -0.1292 4.80
25. BD (1) C 1- C 2 40. BD (1) C 13- C 16 0.0828 1.34
25. BD (1) C 1- C 2 42. BD (1) C 16- H 18 0.0703 0.63
26. BD (1) C 1- N 6 29. BD (1) C 2- O 3 -0.1015 1.78
26. BD (1) C 1- N 6 31. BD (1) C 2- O 4 0.0819 1.38
26. BD (1) C 1- N 6 35. BD (1) C 9- H 10 -0.1106 3.31
26. BD (1) C 1- N 6 37. BD (1) C 9- F 12 0.0629 0.83
26. BD (1) C 1- N 6 38. BD (1) C 13- H 14 -0.1332 4.50
26. BD (1) C 1- N 6 39. BD (1) C 13- H 15 0.0738 1.02
26. BD (1) C 1- N 6 40. BD (1) C 13- C 16 0.0601 0.81
27. BD (1) C 1- C 9 29. BD (1) C 2- O 3 0.0549 0.67
27. BD (1) C 1- C 9 30. BD (2) C 2- O 3 -0.1036 2.56
27. BD (1) C 1- C 9 31. BD (1) C 2- O 4 -0.0764 1.44
27. BD (1) C 1- C 9 33. BD (1) N 6- H 7 -0.1385 6.15
27. BD (1) C 1- C 9 34. BD (1) N 6- H 8 0.0886 1.90
27. BD (1) C 1- C 9 38. BD (1) C 13- H 14 0.0967 2.02
27. BD (1) C 1- C 9 39. BD (1) C 13- H 15 0.0860 1.72

27. BD (1) C 1- C 9 40. BD (1) C 13- C 16 -0.1511 4.85
28. BD (1) C 1- C 13 30. BD (2) C 2- O 3 0.1328 4.05
28. BD (1) C 1- C 13 33. BD (1) N 6- H 7 0.0795 1.52
28. BD (1) C 1- C 13 34. BD (1) N 6- H 8 -0.1312 5.50
28. BD (1) C 1- C 13 35. BD (1) C 9- H 10 0.0619 0.79
28. BD (1) C 1- C 13 36. BD (1) C 9- F 11 0.0566 0.73
28. BD (1) C 1- C 13 37. BD (1) C 9- F 12 -0.1153 3.46
28. BD (1) C 1- C 13 41. BD (1) C 16- H 17 0.0686 1.00
28. BD (1) C 1- C 13 42. BD (1) C 16- H 18 0.0850 1.54
28. BD (1) C 1- C 13 43. BD (1) C 16- C 19 -0.1489 4.44
29. BD (1) C 2- O 3 32. BD (1) O 4- H 5 0.0812 2.05
30. BD (2) C 2- O 3 35. BD (1) C 9- H 10 -0.0509 0.51
30. BD (2) C 2- O 3 38. BD (1) C 13- H 14 0.0644 0.61
30. BD (2) C 2- O 3 42. BD (1) C 16- H 18 0.0760 0.81
33. BD (1) N 6- H 7 41. BD (1) C 16- H 17 0.0711 1.19
35. BD (1) C 9- H 10 38. BD (1) C 13- H 14 0.0760 0.79
38. BD (1) C 13- H 14 41. BD (1) C 16- H 17 -0.1246 5.35
38. BD (1) C 13- H 14 42. BD (1) C 16- H 18 0.0741 0.87
38. BD (1) C 13- H 14 43. BD (1) C 16- C 19 0.0848 1.68
38. BD (1) C 13- H 14 48. BD (1) N 22- H 24 0.0525 0.73
39. BD (1) C 13- H 15 41. BD (1) C 16- H 17 0.0863 1.39
39. BD (1) C 13- H 15 42. BD (1) C 16- H 18 -0.1222 5.26
39. BD (1) C 13- H 15 43. BD (1) C 16- C 19 0.0695 1.17
39. BD (1) C 13- H 15 45. BD (1) C 19- H 21 0.0733 0.69
40. BD (1) C 13- C 16 44. BD (1) C 19- H 20 -0.1322 4.61
40. BD (1) C 13- C 16 45. BD (1) C 19- H 21 0.0815 1.34
41. BD (1) C 16- H 17 44. BD (1) C 19- H 20 0.0940 2.13
41. BD (1) C 16- H 17 45. BD (1) C 19- H 21 0.0745 1.01
41. BD (1) C 16- H 17 46. BD (1) C 19- N 22 -0.1223 3.93
42. BD (1) C 16- H 18 44. BD (1) C 19- H 20 0.0793 1.28
42. BD (1) C 16- H 18 45. BD (1) C 19- H 21 -0.1100 4.58
42. BD (1) C 16- H 18 46. BD (1) C 19- N 22 0.0662 0.80
43. BD (1) C 16- C 19 47. BD (1) N 22- H 23 0.0614 0.93
44. BD (1) C 19- H 20 47. BD (1) N 22- H 23 0.0713 1.10
44. BD (1) C 19- H 20 48. BD (1) N 22- H 24 -0.1089 5.06
45. BD (1) C 19- H 21 47. BD (1) N 22- H 23 -0.1065 5.02
45. BD (1) C 19- H 21 48. BD (1) N 22- H 24 0.0882 1.85
sum within unit 1: 294.94

Total disjoint NLMO steric exchange energy from pairwise sum: 294.94

Conformer **13**

Standard orientation:

Center Atomic Atomic Coordinates (Angstroms)

Number Number Type X Y Z

----

1 6 0 -0.559151 -0.203915 0.205596

2 6 0 -0.000443 1.205903 -0.102031

3 8 0 0.351950 1.559998 -1.201761

4 8 0 0.036937 2.014535 0.972827

5 1 0 0.380155 2.871425 0.674852

6 7 0 -0.884087 -0.469665 1.591771

7 1 0 -0.060527 -0.409633 2.177042

8 1 0 -1.558386 0.200905 1.943134

9 6 0 -1.859030 -0.268695 -0.623389

10 1 0 -1.693029 -0.029814 -1.674069

11 9 0 -2.443278 -1.494097 -0.539867

12 9 0 -2.763174 0.642110 -0.118766

13 6 0 0.408823 -1.274732 -0.354749

14 1 0 0.642010 -1.016454 -1.390705

15 1 0 -0.146277 -2.213534 -0.367722

16 6 0 1.705848 -1.488110 0.434698

17 1 0 2.213778 -2.350800 -0.011401

18 1 0 1.469545 -1.788234 1.462149

19 6 0 2.696766 -0.310368 0.467785

20 1 0 2.257098 0.542624 0.994031

21 1 0 3.556322 -0.611159 1.075298

22 7 0 3.201668 0.171415 -0.814872

23 1 0 2.466889 0.577669 -1.383756

24 1 0 3.639448 -0.574129 -1.345425

E [DLPNO-CCSD(T)/CBS] = -694.8679292

Pairwise steric exchange energies dE(i,j) (kcal/mol) and associated pre-NLMO overlaps S(i,j) for disjoint (no common atoms) interactions between NLMOs i,j:

Threshold for printing: 0.50 kcal/mol
NLMO (i) NLMO (j) S(i,j) kcal/mol
===
within unit 1
13. LP (1) O 3 25. BD (1) C 1- C 2 -0.0427 0.69
13. LP (1) O 3 31. BD (1) C 2- O 4 -0.0412 0.72
13. LP (1) O 3 47. BD (1) N 22- H 23 0.0541 0.61
14. LP (2) O 3 15. LP (1) O 4 -0.0152 -0.53
14. LP (2) O 3 25. BD (1) C 1- C 2 0.1741 11.94
14. LP (2) O 3 28. BD (1) C 1- C 13 0.1236 1.00
14. LP (2) O 3 31. BD (1) C 2- O 4 -0.2049 13.14
14. LP (2) O 3 32. BD (1) O 4- H 5 -0.1366 2.33
14. LP (2) O 3 35. BD (1) C 9- H 10 0.0830 0.90
14. LP (2) O 3 38. BD (1) C 13- H 14 0.1059 1.53
15. LP (1) O 4 17. LP (1) N 6 -0.0788 1.40
15. LP (1) O 4 25. BD (1) C 1- C 2 0.0921 2.84
15. LP (1) O 4 29. BD (1) C 2- O 3 -0.1308 4.61
15. LP (1) O 4 34. BD (1) N 6- H 8 0.0651 0.73
16. LP (2) O 4 27. BD (1) C 1- C 9 -0.0757 1.00
16. LP (2) O 4 28. BD (1) C 1- C 13 0.0635 0.71
16. LP (2) O 4 30. BD (2) C 2- O 3 -0.1934 13.96
16. LP (2) O 4 44. BD (1) C 19- H 20 0.0786 1.23
17. LP (1) N 6 19. LP (2) F 11 -0.0547 0.60
17. LP (1) N 6 25. BD (1) C 1- C 2 -0.2120 11.73
17. LP (1) N 6 27. BD (1) C 1- C 9 0.0857 1.62
17. LP (1) N 6 28. BD (1) C 1- C 13 0.0856 1.88
17. LP (1) N 6 31. BD (1) C 2- O 4 -0.0859 0.50
17. LP (1) N 6 39. BD (1) C 13- H 15 0.1007 0.85
18. LP (1) F 11 23. LP (3) F 12 -0.0594 -0.62
19. LP (2) F 11 27. BD (1) C 1- C 9 -0.1469 7.63
19. LP (2) F 11 35. BD (1) C 9- H 10 0.1183 7.79
20. LP (3) F 11 21. LP (1) F 12 -0.0606 -0.65
20. LP (3) F 11 23. LP (3) F 12 0.0575 1.75
20. LP (3) F 11 27. BD (1) C 1- C 9 0.0728 1.93
20. LP (3) F 11 28. BD (1) C 1- C 13 0.0826 0.83
20. LP (3) F 11 35. BD (1) C 9- H 10 0.0507 1.27
20. LP (3) F 11 37. BD (1) C 9- F 12 -0.1454 7.23
20. LP (3) F 11 39. BD (1) C 13- H 15 0.0757 1.32
22. LP (2) F 12 26. BD (1) C 1- N 6 -0.0811 0.55
22. LP (2) F 12 27. BD (1) C 1- C 9 -0.1548 8.42
22. LP (2) F 12 34. BD (1) N 6- H 8 -0.0718 0.95
22. LP (2) F 12 35. BD (1) C 9- H 10 0.1096 6.57
23. LP (3) F 12 25. BD (1) C 1- C 2 0.0818 1.00
23. LP (3) F 12 27. BD (1) C 1- C 9 0.0705 1.69
23. LP (3) F 12 35. BD (1) C 9- H 10 0.0635 1.90
23. LP (3) F 12 36. BD (1) C 9- F 11 -0.1400 6.62
24. LP (1) N 22 38. BD (1) C 13- H 14 -0.0848 0.68
24. LP (1) N 22 43. BD (1) C 16- C 19 -0.2271 12.06
24. LP (1) N 22 44. BD (1) C 19- H 20 0.0528 1.16
24. LP (1) N 22 45. BD (1) C 19- H 21 0.0782 2.29
25. BD (1) C 1- C 2 32. BD (1) O 4- H 5 -0.1034 4.46
25. BD (1) C 1- C 2 33. BD (1) N 6- H 7 0.0634 0.70
25. BD (1) C 1- C 2 34. BD (1) N 6- H 8 0.0684 0.79
25. BD (1) C 1- C 2 35. BD (1) C 9- H 10 0.0967 1.64
25. BD (1) C 1- C 2 36. BD (1) C 9- F 11 -0.1096 3.20
25. BD (1) C 1- C 2 37. BD (1) C 9- F 12 0.0595 0.59
25. BD (1) C 1- C 2 38. BD (1) C 13- H 14 0.1045 1.84
25. BD (1) C 1- C 2 39. BD (1) C 13- H 15 -0.1314 4.80
25. BD (1) C 1- C 2 40. BD (1) C 13- C 16 0.0544 0.55
26. BD (1) C 1- N 6 29. BD (1) C 2- O 3 -0.1024 1.78
26. BD (1) C 1- N 6 31. BD (1) C 2- O 4 0.0826 1.38
26. BD (1) C 1- N 6 35. BD (1) C 9- H 10 -0.1097 3.28
26. BD (1) C 1- N 6 37. BD (1) C 9- F 12 0.0587 0.71
26. BD (1) C 1- N 6 38. BD (1) C 13- H 14 -0.1324 4.39
26. BD (1) C 1- N 6 40. BD (1) C 13- C 16 0.0743 1.23
27. BD (1) C 1- C 9 30. BD (2) C 2- O 3 0.1355 4.26
27. BD (1) C 1- C 9 31. BD (1) C 2- O 4 -0.0510 0.68
27. BD (1) C 1- C 9 33. BD (1) N 6- H 7 -0.1388 6.22
27. BD (1) C 1- C 9 34. BD (1) N 6- H 8 0.0853 1.76
27. BD (1) C 1- C 9 38. BD (1) C 13- H 14 0.0774 1.21
27. BD (1) C 1- C 9 39. BD (1) C 13- H 15 0.1090 2.76
27. BD (1) C 1- C 9 40. BD (1) C 13- C 16 -0.1482 4.71

28. BD (1) C 1- C 13 29. BD (1) C 2- O 3 0.0639 1.10
28. BD (1) C 1- C 13 30. BD (2) C 2- O 3 -0.0972 2.24
28. BD (1) C 1- C 13 31. BD (1) C 2- O 4 -0.0722 1.11
28. BD (1) C 1- C 13 33. BD (1) N 6- H 7 0.0809 1.62
28. BD (1) C 1- C 13 34. BD (1) N 6- H 8 -0.1325 5.57
28. BD (1) C 1- C 13 35. BD (1) C 9- H 10 0.0664 1.00
28. BD (1) C 1- C 13 36. BD (1) C 9- F 11 0.0517 0.64
28. BD (1) C 1- C 13 37. BD (1) C 9- F 12 -0.1156 3.39
28. BD (1) C 1- C 13 41. BD (1) C 16- H 17 -0.1416 4.80
28. BD (1) C 1- C 13 42. BD (1) C 16- H 18 0.0723 1.01
28. BD (1) C 1- C 13 43. BD (1) C 16- C 19 0.0531 0.57
29. BD (1) C 2- O 3 32. BD (1) O 4- H 5 0.0807 2.00
30. BD (2) C 2- O 3 35. BD (1) C 9- H 10 0.0741 0.96
30. BD (2) C 2- O 3 44. BD (1) C 19- H 20 -0.0805 0.95
30. BD (2) C 2- O 3 47. BD (1) N 22- H 23 -0.0795 1.40
33. BD (1) N 6- H 7 42. BD (1) C 16- H 18 0.0815 1.50
35. BD (1) C 9- H 10 38. BD (1) C 13- H 14 0.0727 0.73
38. BD (1) C 13- H 14 41. BD (1) C 16- H 17 0.0800 1.35
38. BD (1) C 13- H 14 42. BD (1) C 16- H 18 -0.1243 5.26
38. BD (1) C 13- H 14 43. BD (1) C 16- C 19 0.0702 1.03
38. BD (1) C 13- H 14 47. BD (1) N 22- H 23 0.0481 0.54
39. BD (1) C 13- H 15 41. BD (1) C 16- H 17 0.0912 2.31
39. BD (1) C 13- H 15 42. BD (1) C 16- H 18 0.0754 1.23
39. BD (1) C 13- H 15 43. BD (1) C 16- C 19 -0.1454 5.33
40. BD (1) C 13- C 16 44. BD (1) C 19- H 20 0.0724 0.93
40. BD (1) C 13- C 16 45. BD (1) C 19- H 21 -0.1312 4.49
40. BD (1) C 13- C 16 46. BD (1) C 19- N 22 0.0518 0.54
41. BD (1) C 16- H 17 44. BD (1) C 19- H 20 -0.1162 4.88
41. BD (1) C 16- H 17 45. BD (1) C 19- H 21 0.0872 1.74
41. BD (1) C 16- H 17 46. BD (1) C 19- N 22 0.0577 0.63
42. BD (1) C 16- H 18 44. BD (1) C 19- H 20 0.0825 1.29
42. BD (1) C 16- H 18 45. BD (1) C 19- H 21 0.0880 1.83
42. BD (1) C 16- H 18 46. BD (1) C 19- N 22 -0.1233 3.97
43. BD (1) C 16- C 19 48. BD (1) N 22- H 24 0.0609 0.88
44. BD (1) C 19- H 20 47. BD (1) N 22- H 23 0.0885 1.82
44. BD (1) C 19- H 20 48. BD (1) N 22- H 24 -0.1020 4.78
45. BD (1) C 19- H 21 47. BD (1) N 22- H 23 -0.1082 5.01
45. BD (1) C 19- H 21 48. BD (1) N 22- H 24 0.0724 1.18
sum within unit 1: 298.47

Total disjoint NLMO steric exchange energy from pairwise sum: 298.47

Conformer **14**

Standard orientation:

Center Atomic Atomic Coordinates (Angstroms)

Number Number Type X Y Z

----

1 6 0 -0.835233 0.026865 0.420887

2 6 0 -0.975513 1.331164 -0.385722

3 8 0 -0.901497 1.429549 -1.584714

4 8 0 -1.150232 2.399603 0.425152

5 1 0 -1.194855 3.185477 -0.140229

6 7 0 -1.724598 -0.070997 1.568952

7 1 0 -1.556487 0.691482 2.214518

8 1 0 -2.697864 -0.038897 1.285651

9 6 0 -1.151695 -1.129308 -0.539937

10 1 0 -0.660734 -1.030192 -1.505147

11 9 0 -0.797452 -2.326993 0.011933

12 9 0 -2.508662 -1.174996 -0.765713

13 6 0 0.624964 -0.066344 0.926861

14 1 0 0.676154 -0.977332 1.526757

15 1 0 0.780073 0.775416 1.609925

16 6 0 1.721313 -0.068164 -0.141082

17 1 0 1.633397 0.809222 -0.790990

18 1 0 1.619232 -0.946635 -0.787485

19 6 0 3.122244 -0.084112 0.488813

20 1 0 3.238994 0.795271 1.131625

21 1 0 3.215932 -0.956110 1.144246

22 7 0 4.244404 -0.106257 -0.447568

23 1 0 4.219242 0.695748 -1.069158

24 1 0 4.220644 -0.938581 -1.027797

E [DLPNO-CCSD(T)/CBS] = -694.8677014

Pairwise steric exchange energies dE(i,j) (kcal/mol) and associated pre-NLMO overlaps S(i,j) for disjoint (no common atoms) interactions between NLMOs i,j:

Threshold for printing: 0.50 kcal/mol
NLMO (i) NLMO (j) S(i,j) kcal/mol
===
within unit 1
13. LP (1) O 3 25. BD (1) C 1- C 2 -0.0459 0.71
13. LP (1) O 3 31. BD (1) C 2- O 4 -0.0426 0.73
14. LP (2) O 3 15. LP (1) O 4 -0.0172 -0.60
14. LP (2) O 3 25. BD (1) C 1- C 2 0.1721 11.85
14. LP (2) O 3 27. BD (1) C 1- C 9 0.1413 1.76
14. LP (2) O 3 31. BD (1) C 2- O 4 -0.2072 13.53
14. LP (2) O 3 32. BD (1) O 4- H 5 -0.1369 2.27
14. LP (2) O 3 35. BD (1) C 9- H 10 0.1186 2.36
15. LP (1) O 4 17. LP (1) N 6 -0.0571 0.76
15. LP (1) O 4 25. BD (1) C 1- C 2 0.0986 3.07
15. LP (1) O 4 29. BD (1) C 2- O 3 -0.1268 4.44
15. LP (1) O 4 33. BD (1) N 6- H 7 0.0769 1.09
16. LP (2) O 4 28. BD (1) C 1- C 13 0.0904 1.48
16. LP (2) O 4 30. BD (2) C 2- O 3 0.1924 13.76
16. LP (2) O 4 39. BD (1) C 13- H 15 0.0585 0.57
17. LP (1) N 6 25. BD (1) C 1- C 2 -0.2133 11.89
17. LP (1) N 6 27. BD (1) C 1- C 9 0.0818 1.47
17. LP (1) N 6 28. BD (1) C 1- C 13 0.0885 2.05
17. LP (1) N 6 38. BD (1) C 13- H 14 0.1133 1.40
18. LP (1) F 11 23. LP (3) F 12 -0.0599 -0.64
19. LP (2) F 11 27. BD (1) C 1- C 9 -0.1514 7.97
19. LP (2) F 11 35. BD (1) C 9- H 10 0.1163 7.51
20. LP (3) F 11 21. LP (1) F 12 -0.0589 -0.64
20. LP (3) F 11 23. LP (3) F 12 0.0559 1.69
20. LP (3) F 11 27. BD (1) C 1- C 9 0.0741 1.85
20. LP (3) F 11 28. BD (1) C 1- C 13 0.0872 0.95
20. LP (3) F 11 35. BD (1) C 9- H 10 0.0582 1.58
20. LP (3) F 11 37. BD (1) C 9- F 12 -0.1437 7.04
20. LP (3) F 11 38. BD (1) C 13- H 14 0.0633 0.78
22. LP (2) F 12 26. BD (1) C 1- N 6 -0.0855 0.74
22. LP (2) F 12 27. BD (1) C 1- C 9 -0.1525 8.08
22. LP (2) F 12 34. BD (1) N 6- H 8 -0.0805 1.31
22. LP (2) F 12 35. BD (1) C 9- H 10 0.1118 6.89
23. LP (3) F 12 25. BD (1) C 1- C 2 0.0742 0.72
23. LP (3) F 12 27. BD (1) C 1- C 9 0.0723 1.83
23. LP (3) F 12 35. BD (1) C 9- H 10 0.0578 1.57
23. LP (3) F 12 36. BD (1) C 9- F 11 -0.1418 6.74
24. LP (1) N 22 41. BD (1) C 16- H 17 -0.0912 0.63
24. LP (1) N 22 42. BD (1) C 16- H 18 -0.0933 0.70
24. LP (1) N 22 43. BD (1) C 16- C 19 -0.2234 12.06
24. LP (1) N 22 44. BD (1) C 19- H 20 0.0679 1.73
24. LP (1) N 22 45. BD (1) C 19- H 21 0.0613 1.43
25. BD (1) C 1- C 2 32. BD (1) O 4- H 5 -0.1003 4.21
25. BD (1) C 1- C 2 33. BD (1) N 6- H 7 0.0651 0.69
25. BD (1) C 1- C 2 34. BD (1) N 6- H 8 0.0688 0.84
25. BD (1) C 1- C 2 35. BD (1) C 9- H 10 0.1018 1.91
25. BD (1) C 1- C 2 36. BD (1) C 9- F 11 -0.1097 3.28
25. BD (1) C 1- C 2 38. BD (1) C 13- H 14 -0.1300 4.74
25. BD (1) C 1- C 2 39. BD (1) C 13- H 15 0.0935 1.60
25. BD (1) C 1- C 2 40. BD (1) C 13- C 16 0.0718 0.87
26. BD (1) C 1- N 6 29. BD (1) C 2- O 3 -0.0793 0.99
26. BD (1) C 1- N 6 30. BD (2) C 2- O 3 -0.0816 1.31
26. BD (1) C 1- N 6 31. BD (1) C 2- O 4 0.0670 0.93
26. BD (1) C 1- N 6 35. BD (1) C 9- H 10 -0.1133 3.36
26. BD (1) C 1- N 6 37. BD (1) C 9- F 12 0.0711 1.12
26. BD (1) C 1- N 6 38. BD (1) C 13- H 14 0.0898 1.87
26. BD (1) C 1- N 6 39. BD (1) C 13- H 15 0.0802 1.48
26. BD (1) C 1- N 6 40. BD (1) C 13- C 16 -0.1391 4.03
27. BD (1) C 1- C 9 29. BD (1) C 2- O 3 0.0804 1.43
27. BD (1) C 1- C 9 31. BD (1) C 2- O 4 -0.1118 3.02
27. BD (1) C 1- C 9 33. BD (1) N 6- H 7 -0.1362 5.97
27. BD (1) C 1- C 9 34. BD (1) N 6- H 8 0.0862 1.69
27. BD (1) C 1- C 9 38. BD (1) C 13- H 14 0.0717 1.10
27. BD (1) C 1- C 9 39. BD (1) C 13- H 15 -0.1402 5.13
27. BD (1) C 1- C 9 40. BD (1) C 13- C 16 0.0679 0.89
28. BD (1) C 1- C 13 30. BD (2) C 2- O 3 0.1370 4.19
28. BD (1) C 1- C 13 33. BD (1) N 6- H 7 0.0811 1.69
28. BD (1) C 1- C 13 34. BD (1) N 6- H 8 -0.1309 5.58

28. BD (1) C 1- C 13 36. BD (1) C 9- F 11 0.0660 1.02
28. BD (1) C 1- C 13 37. BD (1) C 9- F 12 -0.1105 3.20
28. BD (1) C 1- C 13 41. BD (1) C 16- H 17 0.0794 1.36
28. BD (1) C 1- C 13 42. BD (1) C 16- H 18 0.0654 0.91
28. BD (1) C 1- C 13 43. BD (1) C 16- C 19 -0.1439 4.21
29. BD (1) C 2- O 3 32. BD (1) O 4- H 5 0.0814 2.00
30. BD (2) C 2- O 3 41. BD (1) C 16- H 17 0.0891 1.47
33. BD (1) N 6- H 7 39. BD (1) C 13- H 15 0.0771 0.77
35. BD (1) C 9- H 10 42. BD (1) C 16- H 18 0.0633 0.94
38. BD (1) C 13- H 14 41. BD (1) C 16- H 17 -0.1283 5.53
38. BD (1) C 13- H 14 42. BD (1) C 16- H 18 0.0823 1.33
38. BD (1) C 13- H 14 43. BD (1) C 16- C 19 0.0715 1.17
38. BD (1) C 13- H 14 45. BD (1) C 19- H 21 0.0699 0.72
39. BD (1) C 13- H 15 41. BD (1) C 16- H 17 0.0668 0.73
39. BD (1) C 13- H 15 42. BD (1) C 16- H 18 -0.1262 5.42
39. BD (1) C 13- H 15 43. BD (1) C 16- C 19 0.0875 1.74
39. BD (1) C 13- H 15 44. BD (1) C 19- H 20 0.0780 0.89
40. BD (1) C 13- C 16 44. BD (1) C 19- H 20 0.0750 1.22
40. BD (1) C 13- C 16 45. BD (1) C 19- H 21 0.0761 1.26
40. BD (1) C 13- C 16 46. BD (1) C 19- N 22 -0.1280 3.38
41. BD (1) C 16- H 17 44. BD (1) C 19- H 20 0.0833 1.27
41. BD (1) C 16- H 17 45. BD (1) C 19- H 21 -0.1144 4.79
41. BD (1) C 16- H 17 46. BD (1) C 19- N 22 0.0650 0.86
42. BD (1) C 16- H 18 44. BD (1) C 19- H 20 -0.1139 4.78
42. BD (1) C 16- H 18 45. BD (1) C 19- H 21 0.0795 1.15
42. BD (1) C 16- H 18 46. BD (1) C 19- N 22 0.0661 0.90
43. BD (1) C 16- C 19 47. BD (1) N 22- H 23 0.0567 0.79
43. BD (1) C 16- C 19 48. BD (1) N 22- H 24 0.0518 0.64
44. BD (1) C 19- H 20 47. BD (1) N 22- H 23 0.0746 1.28
44. BD (1) C 19- H 20 48. BD (1) N 22- H 24 -0.1089 5.13
45. BD (1) C 19- H 21 47. BD (1) N 22- H 23 -0.1096 5.19
45. BD (1) C 19- H 21 48. BD (1) N 22- H 24 0.0796 1.51
sum within unit 1: 294.09

Total disjoint NLMO steric exchange energy from pairwise sum: 294.09

Conformer **15**

Standard orientation:

Center Atomic Atomic Coordinates (Angstroms)

Number Number Type X Y Z

----

1 6 0 0.708878 0.026928 0.415434

2 6 0 1.270619 -1.229901 -0.274388

3 8 0 1.489445 -1.349862 -1.453304

4 8 0 1.458876 -2.236296 0.609947

5 1 0 1.778737 -3.002595 0.110247

6 7 0 1.316004 0.325467 1.704997

7 1 0 2.309905 0.504673 1.611882

8 1 0 1.191749 -0.451335 2.343438

9 6 0 0.967681 1.210548 -0.529560

10 1 0 0.714402 0.995602 -1.564902

11 9 0 0.267912 2.312377 -0.126406

12 9 0 2.301987 1.543991 -0.487937

13 6 0 -0.809725 -0.180089 0.629725

14 1 0 -1.158648 0.722442 1.134278

15 1 0 -0.923216 -1.009698 1.335827

16 6 0 -1.644587 -0.460577 -0.620921

17 1 0 -1.270379 -1.350191 -1.139294

18 1 0 -1.558804 0.370687 -1.329723

19 6 0 -3.133561 -0.674389 -0.297235

20 1 0 -3.650761 -0.973533 -1.213564

21 1 0 -3.235661 -1.513365 0.399924

22 7 0 -3.856158 0.463742 0.268601

23 1 0 -3.800254 1.274924 -0.338852

24 1 0 -3.487486 0.734777 1.173171

E [DLPNO-CCSD(T)/CBS] = -694.8673379

Pairwise steric exchange energies dE(i,j) (kcal/mol) and associated pre-NLMO overlaps S(i,j) for disjoint (no common atoms) interactions between NLMOs i,j:

Threshold for printing: 0.50 kcal/mol
NLMO (i) NLMO (j) S(i,j) kcal/mol
===
within unit 1
13. LP (1) O 3 25. BD (1) C 1- C 2 -0.0463 0.72
13. LP (1) O 3 31. BD (1) C 2- O 4 -0.0428 0.74
14. LP (2) O 3 15. LP (1) O 4 -0.0172 -0.60
14. LP (2) O 3 25. BD (1) C 1- C 2 0.1723 11.86
14. LP (2) O 3 27. BD (1) C 1- C 9 0.1417 1.79
14. LP (2) O 3 31. BD (1) C 2- O 4 -0.2071 13.52
14. LP (2) O 3 32. BD (1) O 4- H 5 -0.1367 2.26
14. LP (2) O 3 35. BD (1) C 9- H 10 0.1185 2.36
15. LP (1) O 4 17. LP (1) N 6 -0.0550 0.70
15. LP (1) O 4 25. BD (1) C 1- C 2 0.0990 3.08
15. LP (1) O 4 29. BD (1) C 2- O 3 -0.1267 4.43
15. LP (1) O 4 34. BD (1) N 6- H 8 0.0769 1.08
16. LP (2) O 4 28. BD (1) C 1- C 13 0.0907 1.48
16. LP (2) O 4 30. BD (2) C 2- O 3 0.1925 13.77
16. LP (2) O 4 39. BD (1) C 13- H 15 0.0575 0.55
17. LP (1) N 6 25. BD (1) C 1- C 2 -0.2134 11.91
17. LP (1) N 6 27. BD (1) C 1- C 9 0.0798 1.39
17. LP (1) N 6 28. BD (1) C 1- C 13 0.0909 2.15
17. LP (1) N 6 38. BD (1) C 13- H 14 0.1133 1.36
18. LP (1) F 11 23. LP (3) F 12 -0.0599 -0.64
19. LP (2) F 11 27. BD (1) C 1- C 9 -0.1512 7.95
19. LP (2) F 11 35. BD (1) C 9- H 10 0.1166 7.52
20. LP (3) F 11 21. LP (1) F 12 -0.0589 -0.64
20. LP (3) F 11 23. LP (3) F 12 0.0559 1.69
20. LP (3) F 11 27. BD (1) C 1- C 9 0.0741 1.85
20. LP (3) F 11 28. BD (1) C 1- C 13 0.0874 0.95
20. LP (3) F 11 35. BD (1) C 9- H 10 0.0582 1.58
20. LP (3) F 11 37. BD (1) C 9- F 12 -0.1438 7.04
20. LP (3) F 11 38. BD (1) C 13- H 14 0.0667 0.86
22. LP (2) F 12 26. BD (1) C 1- N 6 -0.0856 0.74
22. LP (2) F 12 27. BD (1) C 1- C 9 -0.1523 8.07
22. LP (2) F 12 33. BD (1) N 6- H 7 -0.0817 1.35
22. LP (2) F 12 35. BD (1) C 9- H 10 0.1120 6.91
23. LP (3) F 12 25. BD (1) C 1- C 2 0.0737 0.70
23. LP (3) F 12 27. BD (1) C 1- C 9 0.0723 1.83
23. LP (3) F 12 35. BD (1) C 9- H 10 0.0576 1.56
23. LP (3) F 12 36. BD (1) C 9- F 11 -0.1420 6.76
24. LP (1) N 22 38. BD (1) C 13- H 14 -0.0848 0.73
24. LP (1) N 22 42. BD (1) C 16- H 18 -0.0983 0.59
24. LP (1) N 22 43. BD (1) C 16- C 19 -0.2267 12.03
24. LP (1) N 22 44. BD (1) C 19- H 20 0.0738 2.06
24. LP (1) N 22 45. BD (1) C 19- H 21 0.0513 1.14
25. BD (1) C 1- C 2 32. BD (1) O 4- H 5 -0.1003 4.20
25. BD (1) C 1- C 2 33. BD (1) N 6- H 7 0.0679 0.81
25. BD (1) C 1- C 2 34. BD (1) N 6- H 8 0.0670 0.74
25. BD (1) C 1- C 2 35. BD (1) C 9- H 10 0.1022 1.93
25. BD (1) C 1- C 2 36. BD (1) C 9- F 11 -0.1098 3.28
25. BD (1) C 1- C 2 38. BD (1) C 13- H 14 -0.1309 4.82
25. BD (1) C 1- C 2 39. BD (1) C 13- H 15 0.0903 1.46
25. BD (1) C 1- C 2 40. BD (1) C 13- C 16 0.0741 0.96
26. BD (1) C 1- N 6 29. BD (1) C 2- O 3 -0.0779 0.95
26. BD (1) C 1- N 6 30. BD (2) C 2- O 3 -0.0842 1.40
26. BD (1) C 1- N 6 31. BD (1) C 2- O 4 0.0652 0.88
26. BD (1) C 1- N 6 35. BD (1) C 9- H 10 -0.1131 3.35
26. BD (1) C 1- N 6 37. BD (1) C 9- F 12 0.0718 1.14
26. BD (1) C 1- N 6 38. BD (1) C 13- H 14 0.0877 1.74
26. BD (1) C 1- N 6 39. BD (1) C 13- H 15 0.0840 1.63
26. BD (1) C 1- N 6 40. BD (1) C 13- C 16 -0.1410 4.08
27. BD (1) C 1- C 9 29. BD (1) C 2- O 3 0.0808 1.44
27. BD (1) C 1- C 9 31. BD (1) C 2- O 4 -0.1130 3.09
27. BD (1) C 1- C 9 33. BD (1) N 6- H 7 0.0878 1.76
27. BD (1) C 1- C 9 34. BD (1) N 6- H 8 -0.1360 5.96
27. BD (1) C 1- C 9 38. BD (1) C 13- H 14 0.0760 1.22
27. BD (1) C 1- C 9 39. BD (1) C 13- H 15 -0.1393 5.09
27. BD (1) C 1- C 9 40. BD (1) C 13- C 16 0.0647 0.80
28. BD (1) C 1- C 13 30. BD (2) C 2- O 3 0.1357 4.10
28. BD (1) C 1- C 13 33. BD (1) N 6- H 7 -0.1301 5.52
28. BD (1) C 1- C 13 34. BD (1) N 6- H 8 0.0805 1.65

28. BD (1) C 1- C 13 36. BD (1) C 9- F 11 0.0672 1.05
28. BD (1) C 1- C 13 37. BD (1) C 9- F 12 -0.1098 3.16
28. BD (1) C 1- C 13 41. BD (1) C 16- H 17 0.0782 1.26
28. BD (1) C 1- C 13 42. BD (1) C 16- H 18 0.0735 1.12
28. BD (1) C 1- C 13 43. BD (1) C 16- C 19 -0.1473 4.37
29. BD (1) C 2- O 3 32. BD (1) O 4- H 5 0.0813 1.99
30. BD (2) C 2- O 3 41. BD (1) C 16- H 17 0.0884 1.41
34. BD (1) N 6- H 8 39. BD (1) C 13- H 15 0.0786 0.81
35. BD (1) C 9- H 10 42. BD (1) C 16- H 18 0.0658 1.01
38. BD (1) C 13- H 14 41. BD (1) C 16- H 17 -0.1280 5.54
38. BD (1) C 13- H 14 42. BD (1) C 16- H 18 0.0809 1.17
38. BD (1) C 13- H 14 43. BD (1) C 16- C 19 0.0738 1.29
38. BD (1) C 13- H 14 48. BD (1) N 22- H 24 0.0513 0.71
39. BD (1) C 13- H 15 41. BD (1) C 16- H 17 0.0757 0.99
39. BD (1) C 13- H 15 42. BD (1) C 16- H 18 -0.1228 5.29
39. BD (1) C 13- H 15 43. BD (1) C 16- C 19 0.0792 1.50
39. BD (1) C 13- H 15 45. BD (1) C 19- H 21 0.0797 0.82
40. BD (1) C 13- C 16 44. BD (1) C 19- H 20 -0.1322 4.59
40. BD (1) C 13- C 16 45. BD (1) C 19- H 21 0.0822 1.39
40. BD (1) C 13- C 16 46. BD (1) C 19- N 22 0.0508 0.52
41. BD (1) C 16- H 17 44. BD (1) C 19- H 20 0.0922 2.08
41. BD (1) C 16- H 17 45. BD (1) C 19- H 21 0.0748 1.02
41. BD (1) C 16- H 17 46. BD (1) C 19- N 22 -0.1212 3.89
42. BD (1) C 16- H 18 44. BD (1) C 19- H 20 0.0791 1.27
42. BD (1) C 16- H 18 45. BD (1) C 19- H 21 -0.1104 4.62
42. BD (1) C 16- H 18 46. BD (1) C 19- N 22 0.0657 0.79
43. BD (1) C 16- C 19 47. BD (1) N 22- H 23 0.0590 0.86
43. BD (1) C 16- C 19 48. BD (1) N 22- H 24 0.0494 0.52
44. BD (1) C 19- H 20 47. BD (1) N 22- H 23 0.0738 1.22
44. BD (1) C 19- H 20 48. BD (1) N 22- H 24 -0.1100 5.14
45. BD (1) C 19- H 21 47. BD (1) N 22- H 23 -0.1064 5.01
45. BD (1) C 19- H 21 48. BD (1) N 22- H 24 0.0857 1.71
sum within unit 1: 295.07

Total disjoint NLMO steric exchange energy from pairwise sum: 295.07

Conformer **16**

Standard orientation:

Center Atomic Atomic Coordinates (Angstroms)

Number Number Type X Y Z

----

1 6 0 -0.828372 0.003378 0.461656

2 6 0 -0.998549 1.414214 -0.140866

3 8 0 -1.244406 2.390503 0.518104

4 8 0 -0.805426 1.464104 -1.479121

5 1 0 -0.919805 2.386455 -1.756685

6 7 0 -1.692885 -0.193016 1.615133

7 1 0 -2.669738 -0.169883 1.340455

8 1 0 -1.546948 0.560394 2.278795

9 6 0 -1.166516 -1.063100 -0.588786

10 1 0 -0.648626 -0.923436 -1.534535

11 9 0 -0.875088 -2.312044 -0.120922

12 9 0 -2.519712 -1.034921 -0.848050

13 6 0 0.639919 -0.140618 0.934029

14 1 0 0.698442 -1.112095 1.429101

15 1 0 0.795220 0.619926 1.706367

16 6 0 1.733494 -0.018764 -0.129573

17 1 0 1.672361 0.946762 -0.642599

18 1 0 1.608292 -0.789351 -0.898450

19 6 0 3.133435 -0.166607 0.485810

20 1 0 3.272985 0.601526 1.253961

21 1 0 3.202596 -1.130158 1.000965

22 7 0 4.255574 -0.076411 -0.446501

23 1 0 4.260844 0.815381 -0.930944

24 1 0 4.206955 -0.804721 -1.151587

E [DLPNO-CCSD(T)/CBS] = -694.8672662

Pairwise steric exchange energies dE(i,j) (kcal/mol) and associated pre-NLMO overlaps S(i,j) for disjoint (no common atoms) interactions between NLMOs i,j:

Threshold for printing: 0.50 kcal/mol
NLMO (i) NLMO (j) S(i,j) kcal/mol
===
within unit 1
13. LP (1) O 3 25. BD (1) C 1- C 2 -0.0500 0.76
13. LP (1) O 3 31. BD (1) C 2- O 4 -0.0431 0.76
14. LP (2) O 3 15. LP (1) O 4 -0.0168 -0.58
14. LP (2) O 3 25. BD (1) C 1- C 2 0.1677 11.69
14. LP (2) O 3 26. BD (1) C 1- N 6 0.1211 1.32
14. LP (2) O 3 31. BD (1) C 2- O 4 -0.2082 13.47
14. LP (2) O 3 32. BD (1) O 4- H 5 -0.1368 2.35
14. LP (2) O 3 34. BD (1) N 6- H 8 0.0937 1.30
15. LP (1) O 4 25. BD (1) C 1- C 2 0.1073 3.34
15. LP (1) O 4 29. BD (1) C 2- O 3 -0.1273 4.53
15. LP (1) O 4 35. BD (1) C 9- H 10 0.1129 2.60
16. LP (2) O 4 28. BD (1) C 1- C 13 0.0923 1.43
16. LP (2) O 4 30. BD (2) C 2- O 3 0.1953 14.13
16. LP (2) O 4 41. BD (1) C 16- H 17 0.0755 1.13
17. LP (1) N 6 19. LP (2) F 11 -0.0534 0.53
17. LP (1) N 6 25. BD (1) C 1- C 2 -0.2040 11.52
17. LP (1) N 6 27. BD (1) C 1- C 9 0.0722 1.01
17. LP (1) N 6 28. BD (1) C 1- C 13 0.0986 2.45
17. LP (1) N 6 29. BD (1) C 2- O 3 -0.0779 0.66
17. LP (1) N 6 30. BD (2) C 2- O 3 0.0431 0.52
17. LP (1) N 6 38. BD (1) C 13- H 14 0.1176 1.47
18. LP (1) F 11 23. LP (3) F 12 -0.0599 -0.63
19. LP (2) F 11 27. BD (1) C 1- C 9 -0.1507 7.87
19. LP (2) F 11 35. BD (1) C 9- H 10 0.1169 7.59
20. LP (3) F 11 21. LP (1) F 12 -0.0591 -0.65
20. LP (3) F 11 23. LP (3) F 12 0.0555 1.67
20. LP (3) F 11 27. BD (1) C 1- C 9 0.0737 1.83
20. LP (3) F 11 28. BD (1) C 1- C 13 0.0863 0.92
20. LP (3) F 11 35. BD (1) C 9- H 10 0.0575 1.54
20. LP (3) F 11 37. BD (1) C 9- F 12 -0.1441 7.07
20. LP (3) F 11 38. BD (1) C 13- H 14 0.0653 0.83
22. LP (2) F 12 26. BD (1) C 1- N 6 -0.0834 0.65
22. LP (2) F 12 27. BD (1) C 1- C 9 -0.1535 8.18
22. LP (2) F 12 33. BD (1) N 6- H 7 -0.0797 1.25
22. LP (2) F 12 35. BD (1) C 9- H 10 0.1113 6.81
23. LP (3) F 12 25. BD (1) C 1- C 2 0.0766 0.77
23. LP (3) F 12 27. BD (1) C 1- C 9 0.0706 1.76
23. LP (3) F 12 35. BD (1) C 9- H 10 0.0586 1.64
23. LP (3) F 12 36. BD (1) C 9- F 11 -0.1422 6.76
24. LP (1) N 22 41. BD (1) C 16- H 17 -0.0924 0.65
24. LP (1) N 22 42. BD (1) C 16- H 18 -0.0931 0.68
24. LP (1) N 22 43. BD (1) C 16- C 19 -0.2238 12.09
24. LP (1) N 22 44. BD (1) C 19- H 20 0.0665 1.66
24. LP (1) N 22 45. BD (1) C 19- H 21 0.0627 1.50
25. BD (1) C 1- C 2 32. BD (1) O 4- H 5 -0.0947 3.92
25. BD (1) C 1- C 2 33. BD (1) N 6- H 7 0.0641 0.72
25. BD (1) C 1- C 2 34. BD (1) N 6- H 8 0.0796 1.20
25. BD (1) C 1- C 2 35. BD (1) C 9- H 10 0.0930 1.45
25. BD (1) C 1- C 2 36. BD (1) C 9- F 11 -0.1138 3.42
25. BD (1) C 1- C 2 38. BD (1) C 13- H 14 -0.1299 4.76
25. BD (1) C 1- C 2 39. BD (1) C 13- H 15 0.0923 1.59
25. BD (1) C 1- C 2 40. BD (1) C 13- C 16 0.0700 0.83
26. BD (1) C 1- N 6 29. BD (1) C 2- O 3 0.0562 0.78
26. BD (1) C 1- N 6 30. BD (2) C 2- O 3 -0.0606 0.85
26. BD (1) C 1- N 6 31. BD (1) C 2- O 4 -0.0968 1.93
26. BD (1) C 1- N 6 35. BD (1) C 9- H 10 -0.1161 3.56
26. BD (1) C 1- N 6 37. BD (1) C 9- F 12 0.0654 0.94
26. BD (1) C 1- N 6 38. BD (1) C 13- H 14 0.0877 1.80
26. BD (1) C 1- N 6 39. BD (1) C 13- H 15 0.0841 1.66
26. BD (1) C 1- N 6 40. BD (1) C 13- C 16 -0.1396 4.03
27. BD (1) C 1- C 9 29. BD (1) C 2- O 3 -0.1009 1.96
27. BD (1) C 1- C 9 30. BD (2) C 2- O 3 -0.0571 0.75
27. BD (1) C 1- C 9 31. BD (1) C 2- O 4 0.0912 1.55
27. BD (1) C 1- C 9 33. BD (1) N 6- H 7 0.0944 2.07
27. BD (1) C 1- C 9 34. BD (1) N 6- H 8 -0.1354 5.91
27. BD (1) C 1- C 9 38. BD (1) C 13- H 14 0.0751 1.18
27. BD (1) C 1- C 9 39. BD (1) C 13- H 15 -0.1388 5.00
27. BD (1) C 1- C 9 40. BD (1) C 13- C 16 0.0630 0.76

28. BD (1) C 1- C 13 30. BD (2) C 2- O 3 0.1378 4.33
28. BD (1) C 1- C 13 33. BD (1) N 6- H 7 -0.1313 5.61
28. BD (1) C 1- C 13 34. BD (1) N 6- H 8 0.0748 1.39
28. BD (1) C 1- C 13 35. BD (1) C 9- H 10 0.0575 0.66
28. BD (1) C 1- C 13 36. BD (1) C 9- F 11 0.0608 0.85
28. BD (1) C 1- C 13 37. BD (1) C 9- F 12 -0.1123 3.31
28. BD (1) C 1- C 13 41. BD (1) C 16- H 17 0.0709 1.11
28. BD (1) C 1- C 13 42. BD (1) C 16- H 18 0.0696 1.04
28. BD (1) C 1- C 13 43. BD (1) C 16- C 19 -0.1444 4.26
29. BD (1) C 2- O 3 32. BD (1) O 4- H 5 0.0793 1.95
30. BD (2) C 2- O 3 39. BD (1) C 13- H 15 0.0684 0.62
34. BD (1) N 6- H 8 39. BD (1) C 13- H 15 0.0783 0.79
35. BD (1) C 9- H 10 42. BD (1) C 16- H 18 0.0670 1.04
38. BD (1) C 13- H 14 41. BD (1) C 16- H 17 -0.1278 5.52
38. BD (1) C 13- H 14 42. BD (1) C 16- H 18 0.0769 1.10
38. BD (1) C 13- H 14 43. BD (1) C 16- C 19 0.0781 1.40
38. BD (1) C 13- H 14 45. BD (1) C 19- H 21 0.0734 0.79
39. BD (1) C 13- H 15 41. BD (1) C 16- H 17 0.0721 0.93
39. BD (1) C 13- H 15 42. BD (1) C 16- H 18 -0.1270 5.47
39. BD (1) C 13- H 15 43. BD (1) C 16- C 19 0.0821 1.54
39. BD (1) C 13- H 15 44. BD (1) C 19- H 20 0.0761 0.85
40. BD (1) C 13- C 16 44. BD (1) C 19- H 20 0.0753 1.24
40. BD (1) C 13- C 16 45. BD (1) C 19- H 21 0.0756 1.24
40. BD (1) C 13- C 16 46. BD (1) C 19- N 22 -0.1281 3.39
41. BD (1) C 16- H 17 44. BD (1) C 19- H 20 0.0822 1.23
41. BD (1) C 16- H 17 45. BD (1) C 19- H 21 -0.1140 4.77
41. BD (1) C 16- H 17 46. BD (1) C 19- N 22 0.0656 0.87
42. BD (1) C 16- H 18 44. BD (1) C 19- H 20 -0.1144 4.80
42. BD (1) C 16- H 18 45. BD (1) C 19- H 21 0.0808 1.18
42. BD (1) C 16- H 18 46. BD (1) C 19- N 22 0.0658 0.88
43. BD (1) C 16- C 19 47. BD (1) N 22- H 23 0.0553 0.75
43. BD (1) C 16- C 19 48. BD (1) N 22- H 24 0.0526 0.67
44. BD (1) C 19- H 20 47. BD (1) N 22- H 23 0.0758 1.33
44. BD (1) C 19- H 20 48. BD (1) N 22- H 24 -0.1089 5.13
45. BD (1) C 19- H 21 47. BD (1) N 22- H 23 -0.1093 5.16
45. BD (1) C 19- H 21 48. BD (1) N 22- H 24 0.0786 1.46
sum within unit 1: 293.94

Total disjoint NLMO steric exchange energy from pairwise sum: 293.94

Conformer **17**

Standard orientation:

Center Atomic Atomic Coordinates (Angstroms)

Number Number Type X Y Z

----

1 6 0 -0.696859 -0.044625 0.455849

2 6 0 -1.291671 1.288963 -0.044149

3 8 0 -1.634171 2.177623 0.691256

4 8 0 -1.356294 1.387050 -1.391686

5 1 0 -1.724788 2.258703 -1.604524

6 7 0 -1.274679 -0.443201 1.730447

7 1 0 -1.199608 0.324849 2.389218

8 1 0 -2.261469 -0.657009 1.626448

9 6 0 -0.959390 -1.157034 -0.568292

10 1 0 -0.665354 -0.895781 -1.581815

11 9 0 -0.306231 -2.303203 -0.214792

12 9 0 -2.305533 -1.450222 -0.588625

13 6 0 0.824725 0.157960 0.663570

14 1 0 1.185185 -0.781412 1.085908

15 1 0 0.936155 0.923127 1.439384

16 6 0 1.652541 0.553317 -0.560220

17 1 0 1.294388 1.501454 -0.974798

18 1 0 1.544794 -0.194336 -1.354407

19 6 0 3.148962 0.700922 -0.231162

20 1 0 3.663843 1.089574 -1.114734

21 1 0 3.274105 1.455512 0.552928

22 7 0 3.852983 -0.506346 0.197324

23 1 0 3.493099 -0.864656 1.074581

24 1 0 3.771265 -1.246783 -0.491971

E [DLPNO-CCSD(T)/CBS] = -694.8670141

Pairwise steric exchange energies dE(i,j) (kcal/mol) and associated pre-NLMO overlaps S(i,j) for disjoint (no common atoms) interactions between NLMOs i,j:

Threshold for printing: 0.50 kcal/mol
NLMO (i) NLMO (j) S(i,j) kcal/mol
===
within unit 1
13. LP (1) O 3 25. BD (1) C 1- C 2 -0.0501 0.77
13. LP (1) O 3 31. BD (1) C 2- O 4 -0.0430 0.75
14. LP (2) O 3 15. LP (1) O 4 -0.0168 -0.57
14. LP (2) O 3 25. BD (1) C 1- C 2 0.1682 11.73
14. LP (2) O 3 26. BD (1) C 1- N 6 0.1212 1.31
14. LP (2) O 3 31. BD (1) C 2- O 4 -0.2077 13.43
14. LP (2) O 3 32. BD (1) O 4- H 5 -0.1364 2.33
14. LP (2) O 3 33. BD (1) N 6- H 7 0.0946 1.32
15. LP (1) O 4 25. BD (1) C 1- C 2 0.1073 3.35
15. LP (1) O 4 29. BD (1) C 2- O 3 -0.1273 4.53
15. LP (1) O 4 35. BD (1) C 9- H 10 0.1133 2.61
16. LP (2) O 4 28. BD (1) C 1- C 13 0.0925 1.44
16. LP (2) O 4 30. BD (2) C 2- O 3 -0.1953 14.13
16. LP (2) O 4 41. BD (1) C 16- H 17 0.0751 1.09
17. LP (1) N 6 19. LP (2) F 11 -0.0533 0.53
17. LP (1) N 6 25. BD (1) C 1- C 2 -0.2041 11.52
17. LP (1) N 6 27. BD (1) C 1- C 9 0.0705 0.95
17. LP (1) N 6 28. BD (1) C 1- C 13 0.1005 2.53
17. LP (1) N 6 29. BD (1) C 2- O 3 -0.0776 0.65
17. LP (1) N 6 30. BD (2) C 2- O 3 -0.0435 0.52
17. LP (1) N 6 38. BD (1) C 13- H 14 0.1185 1.48
18. LP (1) F 11 23. LP (3) F 12 -0.0598 -0.63
19. LP (2) F 11 27. BD (1) C 1- C 9 -0.1505 7.85
19. LP (2) F 11 35. BD (1) C 9- H 10 0.1171 7.60
20. LP (3) F 11 21. LP (1) F 12 -0.0590 -0.64
20. LP (3) F 11 23. LP (3) F 12 0.0553 1.67
20. LP (3) F 11 27. BD (1) C 1- C 9 0.0737 1.83
20. LP (3) F 11 28. BD (1) C 1- C 13 0.0865 0.92
20. LP (3) F 11 35. BD (1) C 9- H 10 0.0572 1.53
20. LP (3) F 11 37. BD (1) C 9- F 12 -0.1441 7.06
20. LP (3) F 11 38. BD (1) C 13- H 14 0.0678 0.89
22. LP (2) F 12 26. BD (1) C 1- N 6 -0.0833 0.65
22. LP (2) F 12 27. BD (1) C 1- C 9 -0.1532 8.16
22. LP (2) F 12 34. BD (1) N 6- H 8 -0.0803 1.28
22. LP (2) F 12 35. BD (1) C 9- H 10 0.1115 6.83
23. LP (3) F 12 25. BD (1) C 1- C 2 0.0766 0.77
23. LP (3) F 12 27. BD (1) C 1- C 9 0.0708 1.77
23. LP (3) F 12 35. BD (1) C 9- H 10 0.0586 1.63
23. LP (3) F 12 36. BD (1) C 9- F 11 -0.1423 6.77
24. LP (1) N 22 38. BD (1) C 13- H 14 -0.0863 0.76
24. LP (1) N 22 42. BD (1) C 16- H 18 -0.0989 0.59
24. LP (1) N 22 43. BD (1) C 16- C 19 -0.2270 12.03
24. LP (1) N 22 44. BD (1) C 19- H 20 0.0749 2.12
24. LP (1) N 22 45. BD (1) C 19- H 21 0.0515 1.13
25. BD (1) C 1- C 2 32. BD (1) O 4- H 5 -0.0950 3.93
25. BD (1) C 1- C 2 33. BD (1) N 6- H 7 0.0808 1.25
25. BD (1) C 1- C 2 34. BD (1) N 6- H 8 0.0632 0.69
25. BD (1) C 1- C 2 35. BD (1) C 9- H 10 0.0935 1.46
25. BD (1) C 1- C 2 36. BD (1) C 9- F 11 -0.1141 3.43
25. BD (1) C 1- C 2 38. BD (1) C 13- H 14 -0.1301 4.78
25. BD (1) C 1- C 2 39. BD (1) C 13- H 15 0.0910 1.53
25. BD (1) C 1- C 2 40. BD (1) C 13- C 16 0.0707 0.87
26. BD (1) C 1- N 6 29. BD (1) C 2- O 3 0.0563 0.78
26. BD (1) C 1- N 6 30. BD (2) C 2- O 3 0.0607 0.85
26. BD (1) C 1- N 6 31. BD (1) C 2- O 4 -0.0965 1.93
26. BD (1) C 1- N 6 35. BD (1) C 9- H 10 -0.1154 3.54
26. BD (1) C 1- N 6 37. BD (1) C 9- F 12 0.0661 0.95
26. BD (1) C 1- N 6 38. BD (1) C 13- H 14 0.0877 1.79
26. BD (1) C 1- N 6 39. BD (1) C 13- H 15 0.0861 1.72
26. BD (1) C 1- N 6 40. BD (1) C 13- C 16 -0.1415 4.11
27. BD (1) C 1- C 9 29. BD (1) C 2- O 3 -0.1011 1.97
27. BD (1) C 1- C 9 30. BD (2) C 2- O 3 0.0569 0.74
27. BD (1) C 1- C 9 31. BD (1) C 2- O 4 0.0914 1.55
27. BD (1) C 1- C 9 33. BD (1) N 6- H 7 -0.1352 5.91
27. BD (1) C 1- C 9 34. BD (1) N 6- H 8 0.0954 2.12
27. BD (1) C 1- C 9 38. BD (1) C 13- H 14 0.0781 1.26
27. BD (1) C 1- C 9 39. BD (1) C 13- H 15 -0.1380 4.97
27. BD (1) C 1- C 9 40. BD (1) C 13- C 16 0.0619 0.73

28. BD (1) C 1- C 13 30. BD (2) C 2- O 3 -0.1381 4.35
28. BD (1) C 1- C 13 33. BD (1) N 6- H 7 0.0744 1.36
28. BD (1) C 1- C 13 34. BD (1) N 6- H 8 -0.1304 5.55
28. BD (1) C 1- C 13 35. BD (1) C 9- H 10 0.0577 0.66
28. BD (1) C 1- C 13 36. BD (1) C 9- F 11 0.0617 0.88
28. BD (1) C 1- C 13 37. BD (1) C 9- F 12 -0.1119 3.28
28. BD (1) C 1- C 13 41. BD (1) C 16- H 17 0.0702 1.04
28. BD (1) C 1- C 13 42. BD (1) C 16- H 18 0.0758 1.20
28. BD (1) C 1- C 13 43. BD (1) C 16- C 19 -0.1479 4.40
29. BD (1) C 2- O 3 32. BD (1) O 4- H 5 0.0792 1.94
30. BD (2) C 2- O 3 39. BD (1) C 13- H 15 -0.0681 0.60
33. BD (1) N 6- H 7 39. BD (1) C 13- H 15 0.0789 0.80
35. BD (1) C 9- H 10 42. BD (1) C 16- H 18 0.0693 1.11
38. BD (1) C 13- H 14 41. BD (1) C 16- H 17 -0.1276 5.53
38. BD (1) C 13- H 14 42. BD (1) C 16- H 18 0.0769 1.01
38. BD (1) C 13- H 14 43. BD (1) C 16- C 19 0.0786 1.48
38. BD (1) C 13- H 14 47. BD (1) N 22- H 23 0.0531 0.75
39. BD (1) C 13- H 15 41. BD (1) C 16- H 17 0.0800 1.16
39. BD (1) C 13- H 15 42. BD (1) C 16- H 18 -0.1235 5.32
39. BD (1) C 13- H 15 43. BD (1) C 16- C 19 0.0752 1.36
39. BD (1) C 13- H 15 45. BD (1) C 19- H 21 0.0787 0.80
40. BD (1) C 13- C 16 44. BD (1) C 19- H 20 -0.1320 4.58
40. BD (1) C 13- C 16 45. BD (1) C 19- H 21 0.0818 1.39
40. BD (1) C 13- C 16 46. BD (1) C 19- N 22 0.0496 0.50
41. BD (1) C 16- H 17 44. BD (1) C 19- H 20 0.0934 2.12
41. BD (1) C 16- H 17 45. BD (1) C 19- H 21 0.0747 1.01
41. BD (1) C 16- H 17 46. BD (1) C 19- N 22 -0.1215 3.89
42. BD (1) C 16- H 18 44. BD (1) C 19- H 20 0.0793 1.27
42. BD (1) C 16- H 18 45. BD (1) C 19- H 21 -0.1109 4.64
42. BD (1) C 16- H 18 46. BD (1) C 19- N 22 0.0661 0.80
43. BD (1) C 16- C 19 48. BD (1) N 22- H 24 0.0587 0.86
44. BD (1) C 19- H 20 47. BD (1) N 22- H 23 -0.1098 5.12
44. BD (1) C 19- H 20 48. BD (1) N 22- H 24 0.0733 1.19
45. BD (1) C 19- H 21 47. BD (1) N 22- H 23 0.0867 1.76
45. BD (1) C 19- H 21 48. BD (1) N 22- H 24 -0.1056 4.98
sum within unit 1: 295.07

Total disjoint NLMO steric exchange energy from pairwise sum: 295.07

Conformer **18**

Standard orientation:

Center Atomic Atomic Coordinates (Angstroms)

Number Number Type X Y Z

----

1 6 0 -0.680722 -0.068287 0.424026

2 6 0 -0.840743 1.363853 -0.132412

3 8 0 -0.022094 1.994787 -0.744658

4 8 0 -2.065294 1.864262 0.182904

5 1 0 -2.122600 2.756341 -0.190950

6 7 0 -0.961842 -0.130876 1.857933

7 1 0 -1.804643 0.381153 2.094608

8 1 0 -0.196304 0.257165 2.395033

9 6 0 -1.759075 -0.928916 -0.269632

10 1 0 -2.771839 -0.658515 0.032723

11 9 0 -1.667861 -0.771278 -1.633066

12 9 0 -1.576023 -2.252201 -0.006040

13 6 0 0.719308 -0.612427 0.106211

14 1 0 0.878378 -0.486951 -0.966088

15 1 0 0.712642 -1.683505 0.316794

16 6 0 1.866218 0.047189 0.877722

17 1 0 1.809655 -0.221799 1.939364

18 1 0 1.795350 1.136879 0.808666

19 6 0 3.249735 -0.386255 0.359155

20 1 0 4.013877 0.015108 1.031854

21 1 0 3.335889 -1.476747 0.412552

22 7 0 3.595166 0.022216 -1.001685

23 1 0 3.474835 1.023080 -1.122388

24 1 0 3.007227 -0.433616 -1.690343

E [DLPNO-CCSD(T)/CBS] = -694.8654368

Pairwise steric exchange energies dE(i,j) (kcal/mol) and associated pre-NLMO overlaps S(i,j) for disjoint (no common atoms) interactions between NLMOs i,j:

Threshold for printing: 0.50 kcal/mol
NLMO (i) NLMO (j) S(i,j) kcal/mol
===
within unit 1
13. LP (1) O 3 25. BD (1) C 1- C 2 -0.0508 0.82
13. LP (1) O 3 31. BD (1) C 2- O 4 -0.0402 0.69
14. LP (2) O 3 15. LP (1) O 4 -0.0159 -0.55
14. LP (2) O 3 25. BD (1) C 1- C 2 0.1645 11.09
14. LP (2) O 3 28. BD (1) C 1- C 13 0.1475 1.95
14. LP (2) O 3 31. BD (1) C 2- O 4 -0.2088 13.74
14. LP (2) O 3 32. BD (1) O 4- H 5 -0.1361 2.24
14. LP (2) O 3 38. BD (1) C 13- H 14 0.1023 1.27
14. LP (2) O 3 42. BD (1) C 16- H 18 0.0839 1.05
15. LP (1) O 4 25. BD (1) C 1- C 2 0.1073 3.45
15. LP (1) O 4 29. BD (1) C 2- O 3 -0.1258 4.35
15. LP (1) O 4 33. BD (1) N 6- H 7 0.0763 1.04
15. LP (1) O 4 35. BD (1) C 9- H 10 0.0712 0.84
16. LP (2) O 4 17. LP (1) N 6 -0.0532 0.65
16. LP (2) O 4 26. BD (1) C 1- N 6 0.0680 0.86
16. LP (2) O 4 27. BD (1) C 1- C 9 -0.0808 1.21
16. LP (2) O 4 30. BD (2) C 2- O 3 -0.1938 13.79
16. LP (2) O 4 33. BD (1) N 6- H 7 0.0487 0.52
17. LP (1) N 6 23. LP (3) F 12 -0.0580 0.63
17. LP (1) N 6 25. BD (1) C 1- C 2 -0.2086 11.63
17. LP (1) N 6 27. BD (1) C 1- C 9 0.1304 4.09
17. LP (1) N 6 28. BD (1) C 1- C 13 0.0465 0.54
17. LP (1) N 6 30. BD (2) C 2- O 3 0.0490 0.58
17. LP (1) N 6 39. BD (1) C 13- H 15 0.0848 0.62
18. LP (1) F 11 23. LP (3) F 12 0.0620 -0.68
19. LP (2) F 11 27. BD (1) C 1- C 9 -0.1557 8.38
19. LP (2) F 11 35. BD (1) C 9- H 10 0.1096 6.74
19. LP (2) F 11 38. BD (1) C 13- H 14 -0.0637 0.72
20. LP (3) F 11 21. LP (1) F 12 -0.0583 -0.64
20. LP (3) F 11 23. LP (3) F 12 -0.0579 1.76
20. LP (3) F 11 25. BD (1) C 1- C 2 0.0877 1.16
20. LP (3) F 11 27. BD (1) C 1- C 9 0.0746 1.87
20. LP (3) F 11 30. BD (2) C 2- O 3 0.0516 0.53
20. LP (3) F 11 35. BD (1) C 9- H 10 0.0597 1.71
20. LP (3) F 11 37. BD (1) C 9- F 12 -0.1409 6.73
21. LP (1) F 12 39. BD (1) C 13- H 15 0.0436 0.58
22. LP (2) F 12 27. BD (1) C 1- C 9 -0.1457 7.47
22. LP (2) F 12 28. BD (1) C 1- C 13 -0.0862 0.75
22. LP (2) F 12 35. BD (1) C 9- H 10 0.1164 7.70
22. LP (2) F 12 39. BD (1) C 13- H 15 -0.0849 1.57
23. LP (3) F 12 26. BD (1) C 1- N 6 -0.0673 0.51
23. LP (3) F 12 27. BD (1) C 1- C 9 -0.0754 2.09
23. LP (3) F 12 35. BD (1) C 9- H 10 -0.0489 1.15
23. LP (3) F 12 36. BD (1) C 9- F 11 0.1471 7.26
24. LP (1) N 22 38. BD (1) C 13- H 14 -0.0886 0.81
24. LP (1) N 22 42. BD (1) C 16- H 18 -0.1007 0.61
24. LP (1) N 22 43. BD (1) C 16- C 19 -0.2245 11.77
24. LP (1) N 22 44. BD (1) C 19- H 20 0.0816 2.47
24. LP (1) N 22 45. BD (1) C 19- H 21 0.0427 0.82
25. BD (1) C 1- C 2 32. BD (1) O 4- H 5 -0.0986 4.07
25. BD (1) C 1- C 2 33. BD (1) N 6- H 7 0.0970 1.88
25. BD (1) C 1- C 2 35. BD (1) C 9- H 10 0.0752 0.86
25. BD (1) C 1- C 2 36. BD (1) C 9- F 11 0.0712 0.89
25. BD (1) C 1- C 2 37. BD (1) C 9- F 12 -0.1083 3.16
25. BD (1) C 1- C 2 38. BD (1) C 13- H 14 0.1051 1.98
25. BD (1) C 1- C 2 39. BD (1) C 13- H 15 -0.1289 4.68
25. BD (1) C 1- C 2 40. BD (1) C 13- C 16 0.0520 0.51
26. BD (1) C 1- N 6 29. BD (1) C 2- O 3 -0.0627 0.58
26. BD (1) C 1- N 6 30. BD (2) C 2- O 3 -0.1024 2.15
26. BD (1) C 1- N 6 31. BD (1) C 2- O 4 0.0561 0.60
26. BD (1) C 1- N 6 35. BD (1) C 9- H 10 0.0815 1.46
26. BD (1) C 1- N 6 36. BD (1) C 9- F 11 -0.1109 2.89
26. BD (1) C 1- N 6 38. BD (1) C 13- H 14 -0.1388 4.75
26. BD (1) C 1- N 6 40. BD (1) C 13- C 16 0.0784 1.31
27. BD (1) C 1- C 9 29. BD (1) C 2- O 3 -0.0668 0.82
27. BD (1) C 1- C 9 30. BD (2) C 2- O 3 0.1086 2.68
27. BD (1) C 1- C 9 31. BD (1) C 2- O 4 0.0597 0.59
27. BD (1) C 1- C 9 33. BD (1) N 6- H 7 0.0613 0.73

27. BD (1) C 1- C 9 34. BD (1) N 6- H 8 -0.1296 5.49
27. BD (1) C 1- C 9 38. BD (1) C 13- H 14 0.0715 1.08
27. BD (1) C 1- C 9 39. BD (1) C 13- H 15 0.1029 2.44
27. BD (1) C 1- C 9 40. BD (1) C 13- C 16 -0.1535 5.03
28. BD (1) C 1- C 13 29. BD (1) C 2- O 3 0.0787 1.58
28. BD (1) C 1- C 13 31. BD (1) C 2- O 4 -0.1115 2.90
28. BD (1) C 1- C 13 33. BD (1) N 6- H 7 -0.1263 5.08
28. BD (1) C 1- C 13 34. BD (1) N 6- H 8 0.1078 2.95
28. BD (1) C 1- C 13 35. BD (1) C 9- H 10 -0.1061 3.36
28. BD (1) C 1- C 13 37. BD (1) C 9- F 12 0.0714 1.17
28. BD (1) C 1- C 13 41. BD (1) C 16- H 17 0.0530 0.56
28. BD (1) C 1- C 13 42. BD (1) C 16- H 18 0.0915 1.83
28. BD (1) C 1- C 13 43. BD (1) C 16- C 19 -0.1469 4.22
29. BD (1) C 2- O 3 32. BD (1) O 4- H 5 0.0778 1.81
30. BD (2) C 2- O 3 42. BD (1) C 16- H 18 -0.0626 0.80
34. BD (1) N 6- H 8 41. BD (1) C 16- H 17 0.0856 1.73
38. BD (1) C 13- H 14 41. BD (1) C 16- H 17 -0.1220 5.14
38. BD (1) C 13- H 14 43. BD (1) C 16- C 19 0.1003 2.37
38. BD (1) C 13- H 14 48. BD (1) N 22- H 24 0.0568 0.85
39. BD (1) C 13- H 15 41. BD (1) C 16- H 17 0.1009 2.06
39. BD (1) C 13- H 15 42. BD (1) C 16- H 18 -0.1197 5.10
39. BD (1) C 13- H 15 43. BD (1) C 16- C 19 0.0585 0.81
39. BD (1) C 13- H 15 45. BD (1) C 19- H 21 0.0687 0.59
40. BD (1) C 13- C 16 44. BD (1) C 19- H 20 -0.1319 4.61
40. BD (1) C 13- C 16 45. BD (1) C 19- H 21 0.0822 1.38
41. BD (1) C 16- H 17 44. BD (1) C 19- H 20 0.0961 2.20
41. BD (1) C 16- H 17 45. BD (1) C 19- H 21 0.0718 0.92
41. BD (1) C 16- H 17 46. BD (1) C 19- N 22 -0.1221 3.93
42. BD (1) C 16- H 18 44. BD (1) C 19- H 20 0.0788 1.24
42. BD (1) C 16- H 18 45. BD (1) C 19- H 21 -0.1110 4.62
42. BD (1) C 16- H 18 46. BD (1) C 19- N 22 0.0684 0.88
43. BD (1) C 16- C 19 47. BD (1) N 22- H 23 0.0664 1.10
44. BD (1) C 19- H 20 47. BD (1) N 22- H 23 0.0673 0.95
44. BD (1) C 19- H 20 48. BD (1) N 22- H 24 -0.1090 5.05
45. BD (1) C 19- H 21 47. BD (1) N 22- H 23 -0.1073 5.09
45. BD (1) C 19- H 21 48. BD (1) N 22- H 24 0.0906 2.00
sum within unit 1: 296.29

Total disjoint NLMO steric exchange energy from pairwise sum: 296.29
